# Evaluation of the interactions of hydrazide derivatives with acetic acid and molecular modeling analysis of *N*-acetylated hydrazides†

**DOI:** 10.1039/d5ra01286d

**Published:** 2025-04-28

**Authors:** Hamid Beyzaei, Sakineh Sheikh, Fereshteh Shiri, Reza Aryan

**Affiliations:** a Department of Chemistry, Faculty of Science, University of Zabol Zabol Iran hbeyzaei@yahoo.com hbeyzaei@uoz.ac.ir +98 54 31232180 +98 54-31232186

## Abstract

Acetic acid, as a weak organic acid, has a wide range of food, pharmaceutical, and industrial applications. It is also used as a green solvent, catalyst, and reagent in chemical experiments. Properties such as non-toxicity, safety, availability, and low cost have made it the preferred choice for acetylation processes. In this project, the interactions of a series of alkyl/aryl/heteroaryl hydrazides with acetic acid were investigated under reflux heating. A variety of reactions, including *C*- and *N*-acetylation, hydrolysis, and rearrangement, occurred in the presence of acetic acid. Most of the products were recrystallized in good to excellent yields under these conditions without the need for further purification. All synthesized compounds were characterized by NMR (^1^H and ^13^C), FT-IR, and CHNS analysis. In addition, a novel method was proposed for the preparation of products 2a and 2i–q. This method has the potential to be extended to similar reagents. To investigate the biological activity and drug-like properties, some *in silico* methods were employed on the synthesized compounds. Screening using the ChEMBL database revealed that out of 17 synthesized compounds, compounds 2b (ChEMBL93746), 2c (ChEMBL22425), and 2d (ChEMBL441343) exhibited significant activity against targets SIRT1, TPMT, and Tyrosinase, with measured values below 200 μM. Molecular docking demonstrated that compound 2o interacted with all three targets. These findings provide valuable insights into its potential as a promising multi-target drug candidate for future investigations.

## Abstract 2

Acetic acid as a reagent, solvent and catalyst was reacted with hydrazide derivatives under reflux heating. *C*- and *N*-acetylation, hydrolysis and rearrangement were observed in these interactions. The biological activities and pharmacological potentials of the synthesized products were assessed.

## Graphical abstract

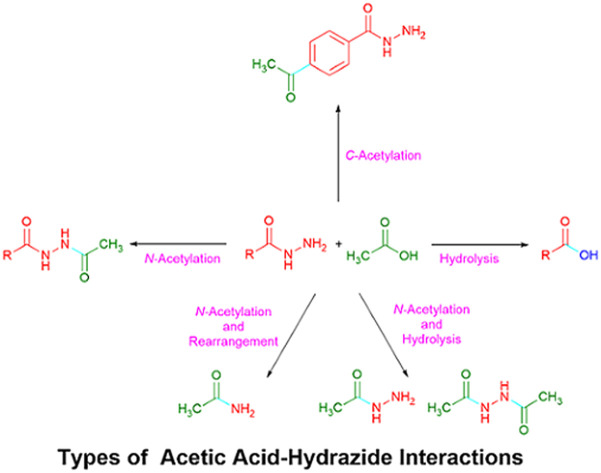

## 1 Introduction

Acetic acid is an aliphatic carboxylic acid with a p*K*_a_ of 4.76. The characteristic odor of vinegar is due to the presence of about 4–6% of this monoprotic acid, which is why it is also known as vinegar acid. Pure acetic acid, known as glacial acetic acid in laboratory grade, is a colorless and corrosive liquid with a boiling point of 117–118 °C. Acetic acid is a byproduct of carbohydrate fermentation and of the destructive distillation of wood. *Acetobacter* and *Gluconobacter*, two main groups of Gram-negative aerobic acetic acid bacteria, oxidize carbohydrates first to ethanol and then to acetic acid.^1^ In addition to gluconic acid and acetic acid, other organic acids and products, such as ketones, can also be produced as a result of the enzymatic activity of these bacteria. Acetic acid bacteria are also efficient microorganisms in the production of cellulose and sorbose.^2,3^

Acetic acid can inhibit the growth of a variety of pathogenic bacterial and fungal strains, which is why it is used as a food preservative and a local antiseptic agent.^4^ Irrigation of nosocomial and burn wounds with acetic acid is one of the most effective and common methods to prevent microbial infections.^5,6^ This medicinal agent is used to flush the urinary bladder,^7^ manage soft tissue injuries *via* iontophoresis,^8^ treat external otitis,^9^ remove ear wax,^10^ and for visual inspection of the cervix in office cervicoscopy and stationary colposcopy.^11^ In organic synthesis, it is applied as a solvent (reaction medium or recrystallization process), reagent, and catalyst in organic reactions.^12–15^

The hydrazide functional group (–C(=O)NR^1^NR^2^R^3^) is present in a wide range of biologically active compounds.^16^ Isoniazid (isonicotinic acid hydrazide), an antibiotic, treats mycobacterial infections, especially tuberculosis. This tuberculostatic agent interferes with the biosynthesis of *Mycobacterium tuberculosis* through inhibition of enoyl-acyl carrier protein reductase (InhA).^17^ Tecovirimat is a P37 protein inhibitor and blocks its interaction with Rab9 GTPase and TIP47.^18^ This antiviral medication is prescribed to treat diseases caused by orthopoxviruses such as monkeypox, smallpox, and cowpox. Cyclooxygenases, precursors of prostaglandins, metabolize arachidonic acid to cyclic endoperoxides. These isoenzymes are inhibited by bumadizone calcium, a non-steroidal antipyretic, anti-inflammatory, and analgesic drug used to treat gout and rheumatoid arthritis.^19^ Cilazapril is a commonly prescribed drug to manage hypertension. It is an inhibitor of pyridazine angiotensin I-converting enzyme (ACE/kininase II), blocks the synthesis of the vasoconstrictor angiotensin II from angiotensin I, and decreases blood pressure.^20^

Diversity in the biological properties of molecules containing the hydrazide functional group motivated us to perform *C*- or *N*-acetylation on some alkyl/aryl/heteroaryl hydrazides using acetic acid. For this purpose, 17 compounds were prepared. All synthesized compounds were assessed *in silico* for their biological activities and drug potential. These theoretical approaches underscore the pharmacological potential of the synthesized compounds and provide a foundation for further optimization.

## 2 Experimental

### 2.1 Materials and methods

All chemicals and solvents were purchased from Sigma-Aldrich and used without further purification. The progress of reactions was monitored by aluminum TLC plates pre-coated with silica gel and a fluorescent indicator (F254). The uncorrected melting points were measured using a Kruss type KSP1N melting point meter. NMR (^1^H and ^13^C) spectra were recorded using a Bruker Avance III 300 MHz spectrometer. FT-IR spectra were collected in KBr disks using a Bruker Tensor 27 FT-IR spectrometer. The elemental composition of the target products was determined using a Thermo Finnigan Flash EA CHNS-O microanalyzer.

### 2.2 General procedure for the reaction of hydrazides with glacial acetic acid

A solution containing 2 mmol of various hydrazides (1a–r) in 1 ml of glacial acetic acid was heated under reflux for 2–8 h. The reaction mixture was then cooled to room temperature. Some products crystallized under these conditions, and soluble products were precipitated by adding the reaction solution to crushed ice. The precipitates were filtered off, washed with cold diethyl ether, and oven-dried at 50 °C. If necessary, the final solids were recrystallized from ethanol or an ethanol–water mixture.

#### 2.2.1 4-Acetylbenzohydrazide (2a)

FT-IR *ν*_max_ 3413, 3129, 1673, 1609, 1517, 1398, 1174, 864, 773, 620, 547, 475 cm^−1^; ^1^H NMR (300 MHz, DMSO-*d*_6_) *δ* 2.10 (s, 3H, CH_3_), 7.71 (d, *J* = 8.6 Hz, 2H, H-3,5 Ph), 7.90 (d, *J* = 8.6 Hz, 2H, H-2,6 Ph), 10.27 (s, 2H, NH_2_), 12.69 (s, 1H, NH) ppm; ^13^C NMR (75 MHz, DMSO-*d*_6_) *δ* 169.34 (CH_3_*C*=O), 167.42 (NHCO), 143.82 (C-4 Ph), 130.86 (C-3,5 Ph), 125. 33 (C-1 Ph), 118.62 (C-2,6 Ph), 24.64 (CH_3_) ppm; anal. calcd for C_9_H_10_N_2_O_2_ (178.19): C 60.66, H 5.66, N 15.72; found: C 60.60, H 5.62, N 15.77.

#### 2.2.2 4-(*tert*-Butyl)benzoic acid (2b)

FT-IR *ν*_max_ 3512, 3239, 2963, 2358, 1640, 1539, 1499, 1368, 1277, 1122, 1022, 848, 769, 699, 561 cm^−1^; ^1^H NMR (300 MHz, DMSO-*d*_6_) *δ* 1.34 (s, 9H, 3 × CH_3_), 7.56 (d, *J* = 8.4 Hz, 2H, H-3,5 Ph), 7.89 (d, *J* = 8.4 Hz, 2H, H-2,6 Ph), 10.44 (s, 1H, OH) ppm; ^13^C NMR (75 MHz, DMSO-*d*_6_) *δ* 166.20 (C=O), 155.15 (C-4 Ph), 130.35 (C-1 Ph), 127.88 (C-2,6 Ph), 125.76 (C-3,5 Ph), 35.19 (*C*(CH_3_)_3_), 31.41 (CH_3_) ppm; anal. calcd for C_11_H_14_O_2_ (178.23): C 74.13, H 7.92; found: C 74.16, H 7.92.

#### 2.2.3 3-Methoxybenzoic acid (2c)

FT-IR *ν*_max_ 3514, 3236, 1636, 1581, 1537, 1480, 1289, 1045, 804, 740, 681 cm^−1^; ^1^H NMR (300 MHz, DMSO-*d*_6_) *δ* 3.85 (s, 3H, CH_3_), 7.18 (m, 1H, H-4 Ph), 7.43–7.55 (m, 3H, H-2,5,6 Ph), 10.50 (br s, 1H, OH) ppm; ^13^C NMR (75 MHz, DMSO-*d*_6_) *δ* 166.95 (C=O), 159.69 (C-3 Ph), 134.49 (C-1 Ph), 130.17 (C-5 Ph), 120.15 (C-6 Ph), 118.19 (C-4 Ph), 112.98 (C-2 Ph), 55.78 (CH_3_) ppm; anal. calcd for C_8_H_8_O_3_ (152.15): C 63.15, H 5.30; found: C 63.20, H 5.33.

#### 2.2.4 4-Hydroxybenzoic acid (2d)

FT-IR *ν*_max_ 3741, 3390, 2668, 2549, 2362, 1681, 1601, 1549, 1513, 1416, 1285, 1240, 1164, 1111, 1013, 931, 849, 772, 690, 651, 614, 544, 499 cm^−1^; ^1^H NMR (300 MHz, DMSO-*d*_6_) *δ* 6.83 (d, *J* = 8.4 Hz, 2H, C-3,5 Ph), 7.81 (d, *J* = 8.4 Hz, 2H, C-2,6 Ph), 11.02 (brs, 2H, 2 × OH) ppm; ^13^C NMR (75 MHz, DMSO-*d*_6_) *δ* 169.10 (C=O), 161.95 (C-4 Ph), 131.95 (C-2,6 Ph), 122.48 (C-1 Ph), 115.51 (C-3,5 Ph) ppm; anal. calcd for C_7_H_6_O_3_ (138.12): C 60.87, H 4.38; found: C 60.91, H 4.39.

#### 2.2.5 Isonicotinic acid (2e)

FT-IR *ν*_max_ 3512, 1645, 1545, 1296, 752, 701 cm^−1^; ^1^H NMR (300 MHz, DMSO-*d*_6_) *δ* 7.85 (d, *J* = 6.0 Hz, 2H, H-3,5 Py), 8.83 (d, *J* = 6.0 Hz, 2H, H-2,6 Py), 11.01 (brs, 1H, OH) ppm; ^13^C NMR (75 MHz, DMSO-*d*_6_) *δ* 164.77 (C=O), 151.03 (C-2,6 Py), 139.77 (C-4 Py), 121.80 (C-3,5 Py) ppm; anal. calcd for C_6_H_5_NO_2_ (123.11): C 58.54, H 4.09, N 11.38; found: C 58.58, H 4.11, N 11.33.

#### 2.2.6 Nicotinic acid (2f)

FT-IR *ν*_max_ 3741, 2363, 1712, 1593, 1413, 1318, 1177, 1032, 817, 751, 689, 636 cm^−1^; ^1^H NMR (300 MHz, DMSO-*d*_6_) *δ* 7.52 (m, 1H, H-5 Py), 8.27 (d, *J* = 7.8 Hz, 1H, H-4 Py), 8.77 (m, 1H, H-6 Py), 9.09 (s, 1H, H-2 Py), 13.02 (brs, 1H, OH) ppm; ^13^C NMR (75 MHz, DMSO-*d*_6_) *δ* 166.78 (C=O), 153.58 (C-6 Py), 150.67 (C-2 Py), 137.41 (C-4 Py), 127.15 (C-3 Py), 124.18 (C-5 Py); anal. calcd for C_6_H_5_NO_2_ (123.11): C 58.54, H 4.09, N 11.38; found: C 58.50, H 4.12, N 11.38.

#### 2.2.7 Acetohydrazide (2g)

FT-IR *ν*_max_ 3225, 1688, 1268, 1015, 631, 545 cm^−1^; ^1^H NMR (300 MHz, DMSO-*d*_6_) *δ* 1.83 (s, 3H, CH_3_), 7.47 (brs, 2H, NH_2_), 9.79 (s, 1H, NH) ppm; ^13^C NMR (75 MHz, DMSO-*d*_6_) *δ* 168.74 (C=O), 20.93 (CH_3_) ppm; anal. calcd for C_2_H_6_N_2_O (74.08): C 32.43, H 8.16, N 37.81; found: C 32.39, H 8.19, N 37.77.

#### 2.2.8 Acetamide (2h)

FT-IR *ν*_max_ 3052, 1697, 1420, 1315, 1203, 923, 805, 637, 578 cm^−1^; ^1^H NMR (300 MHz, DMSO-*d*_6_) *δ* 2.42 (s, 3H, CH_3_), 12.25 (brs, 2H, NH_2_) ppm; ^13^C NMR (75 MHz, DMSO-*d*_6_) *δ* 174.28 (C=O), 29.52 (CH_3_) ppm; anal. calcd for C_2_H_5_NO (59.07): C 40.67, H 8.53, N 23.71; found: C 40.70, H 8.54, N 23.73.

#### 2.2.9 *N*′-Acetyl-4-nitrobenzohydrazide (2i)

FT-IR *ν*_max_ 3515, 3216, 2360, 1693, 1588, 1514, 1471, 1351, 1270, 1107, 1003, 867, 714, 648 cm^−1^; ^1^H NMR (300 MHz, DMSO-*d*_6_) *δ* 1.97 (s, 3H, CH_3_), 8.11 (d, *J* = 7.2 Hz, 2H, H-2,6 Ph), 8.35 (d, *J* = 7.2 Hz, 2H, H-3,5 Ph), 10.08, 10.81 (brs, 2H, 2 × NH) ppm; ^13^C NMR (75 MHz, DMSO-*d*_6_) *δ* 169.97 (HC_3_*C*=O), 164.40 (Ph-*C*=O), 149.78 (C-4 Ph), 138.63 (C-1 Ph), 129.43 (C-2,6 Ph), 124.13 (C-3,5 Ph), 21.02 (CH_3_) ppm; anal. calcd for C_9_H_9_N_3_O_4_ (223.19): C 48.43, H 4.06, N 18.83; found: C 48.39, H 4.12, N 18.78.

#### 2.2.10 *N*′-Acetyl-4-fluorobenzohydrazide (2j)

FT-IR *ν*_max_ 3515, 1660, 1620, 1512, 1424, 1335, 1141, 832, 733 cm^−1^; ^1^H NMR (300 MHz, DMSO-*d*_6_) *δ* 2.03 (s, 3H, CH_3_), 7.25 (d, *J* = 9.6 Hz, 2H, H-3,5 Ph), 8.85 (d, *J* = 9.6 Hz, 2H, H-2,6 Ph), 10.07, 10.44 (brs, 2H, 2 × NH) ppm; ^13^C NMR (75 MHz, DMSO-*d*_6_) *δ* 169.46 (HC_3_*C*=O), 149.02 (Ph-*C*=O), 137.07 (C-4 Ph), 129.99 (C-1 Ph), 123.58 (C-2,6 Ph), 115.91 (C-3,5 Ph), 21.05 (CH_3_) ppm; Anal. Calcd for C_9_H_9_FN_2_O_2_ (196.18): C 55.10, H 4.62, N 14.28; found: C 55.10, H 4.67, N 14.34.

#### 2.2.11 *N*′-Acetyl-4-(trifluoromethyl)benzohydrazide (2k)

FT-IR *ν*_max_ 3513, 2363, 1685, 1549, 1410, 1285, 1018, 757, 620 cm^−1^; ^1^H NMR (300 MHz, DMSO-*d*_6_) *δ* 1.84 (s, 3H, CH_3_), 7.33–7.66 (m, 4H, H-2,3,5,6 Ph), 9.76 (s, 2H, 2 × NH) ppm; ^13^C NMR (75 MHz, DMSO-*d*_6_) *δ* 169.54 (HC_3_*C*=O), 165.51 (Ph-*C*=O), 147.39 (C-1 Ph), 136.35 (C-4 Ph), 127.19 (C-2,6 Ph), 125.41 (C-3,5 Ph), 123.75 (CF_3_), 20.95 (CH_3_) ppm; anal. calcd for C_10_H_9_F_3_N_2_O_2_ (246.19): C 48.79, H 3.68, N 11.38; found: C 48.84, H 3.61, N 11.30.

#### 2.2.12 *N*′-Acetyl-4-hydroxybenzohydrazide (2l)

FT-IR *ν*_max_ 3172, 3018, 1703, 1616, 1568, 1507, 1344, 1278, 1231, 1169, 1041, 1001, 893, 852, 819, 769, 668, 618, 567, 493 cm^−1^; ^1^H NMR (300 MHz, DMSO-*d*_6_) *δ* 1.92 (s, 3H, CH_3_), 6.84 (d, *J* = 8.7 Hz, 2H, H-3,5 Ph), 7.76 (d, *J* = 8.7 Hz, 2H, H-2,6 Ph), 9.06 (s, 1H, OH), 9.80, 10.04 (s, 2H, 2 × NH) ppm; ^13^C NMR (75 MHz, DMSO-*d*_6_) *δ* 169.12 (HC_3_*C*=O), 165.71 (Ph-*C*=O), 161.06 (C-4 Ph), 129.92 (C-2,6 Ph), 123.58 (C-1 Ph), 115.42 (C-3,5 Ph), 21.09 (CH_3_) ppm; anal. calcd for C_9_H_10_N_2_O_3_ (194.19): C 55.67, H 5.19, N 14.43; found: C 55.72, H 5.18, N 14.37.

#### 2.2.13 *N*′-Acetyl-3-hydroxybenzohydrazide (2m)

FT-IR *ν*_max_ 3331, 1650, 1584, 1538, 1491, 1365, 1253, 1168, 994, 857, 799, 747, 673, 555 cm^−1^; ^1^H NMR (300 MHz, DMSO-*d*_6_) *δ* 1.94 (s, 3H, CH_3_), 7.00 (m, 1H, H-2 Ph), 7.26–7.40 (m, 3H, H-4,5,6 Ph), 9.91–10.10 (brs, 3H, OH, 2 × NH) ppm; ^13^C NMR (75 MHz, DMSO-*d*_6_) *δ* 169.15 (HC_3_*C*=O), 166.17 (Ph-*C*=O), 157.89 (C-3 Ph), 134.41 (C-1 Ph), 129.96 (C-5 Ph), 119.21 (C-6 Ph), 118.35 (C-4 Ph), 114.91 (C-2 Ph), 21.07 (CH_3_); anal. calcd for C_9_H_10_N_2_O_3_ (194.19): C 55.67, H 5.19, N 14.43; found: C 55.67, H 5.23, N 14.46.

#### 2.2.14 *N*′-Acetyl-3-bromobenzohydrazide (2n)

FT-IR *ν*_max_ 3431, 1642, 1556, 1296, 735 cm^−1^; ^1^H NMR (300 MHz, DMSO-*d*_6_) *δ* 1.95 (s, 3H, CH_3_), 7.50 (m, 1H, H-5 Ph), 7.79–7.94 (m, 2H, H-4,6 Ph), 8.09 (m, 1H, H-2 Ph), 10.01–10.62 (brs, 2H, 2 × NH) ppm; ^13^C NMR (75 MHz, DMSO-*d*_6_) *δ* 169.01 (HC_3_*C*=O), 164.53 (Ph-*C*=O), 135.03 (C-1 Ph), 131.36 (C-5 Ph), 131.25 (C-2 Ph), 130.58 (C-4 Ph), 127.02 (C-6 Ph), 122.23 (C-3 Ph), 21.07 (CH_3_) ppm; anal. calcd for C_9_H_9_BrN_2_O_2_ (257.09): C 42.05, H 3.53, N 10.90; found: C 42.12, H 3.49, N 10.89.

#### 2.2.15 *N*′-Acetyl-3-hydroxy-2-naphthohydrazide (2o)

FT-IR *ν*_max_ 3026, 1601, 1492, 1359, 1271, 1218, 1162, 1001, 912, 874, 744, 701, 655, 592, 467 cm^−1^; ^1^H NMR (300 MHz, DMSO-*d*_6_) *δ* 2.03 (s, 3H, CH_3_), 7.37 (m, 2H, H–Np), 7.51 (m, 1H, H–Np), 7.77 (d, *J* = 8.3 Hz, 1H, H–Np), 7.92 (d, *J* = 8.3 Hz, 1H, H–Np), 8.59 (s, 1H, H–Np), 10.49, 10.82 (brs, 2H, 2 × NH), 11.57 (s, 1H, OH) ppm; ^13^C NMR (75 MHz, DMSO-*d*_6_) *δ* 169.08 (HC_3_*C*=O), 165.51 (Np–*C*=O), 154.48 (C-3 Np), 136.49 (C-4a Np), 131.18 (C-8a Np), 129.29 (C-1 Np), 128.86 (C-8 Np), 127.27 (C-6 Np), 126.29 (C-5 Np), 124.34 (C-7 Np), 118.96 (C-2 Np), 111.24 (C-4 Np), 20.96 (CH_3_) ppm; anal. calcd for C_13_H_12_N_2_O_3_ (244.25): C 63.93, H 4.95, N 11.47; found: C 70.01, H 4.97, N 11.44.

#### 2.2.16 *N*′-Acetylthiophene-2-carbohydrazide (2p)

FT-IR *ν*_max_ 3742, 3516, 3211, 3012, 2361, 1688, 1629, 1548, 1419, 1365, 1286, 1097, 1002, 846, 723, 608 cm^−1^; ^1^H NMR (300 MHz, DMSO-*d*_6_) *δ* 1.94 (s, 3H, CH_3_), 7.22 (m, 1H, H-4 Th), 7.84–7.92 (m, 2H, H-3,5 Th), 10.35, 10.59 (s, 2H, 2 × NH) ppm; ^13^C NMR (75 MHz, DMSO-*d*_6_) *δ* 169.20 (HC_3_*C*=O), 161.02 (Th–*C*=O), 137.82 (C-2 Th), 132.02 (C-3 Th), 129.32 (C-5 Th), 128.60 (C-4 Th), 21.04 (CH_3_); anal. calcd for C_7_H_8_N_2_O_2_S (184.21): C 45.64, H 4.38, N 15.21, S 17.40; found: C 45.66, H 4.35, N 15.23, S 17.33.

#### 2.2.17 *N*′-Acetylacetohydrazide (2q)

FT-IR *ν*_max_ 3224, 1600, 1506, 1432, 1364, 1258, 1017, 921, 638, 550, 473 cm^−1^; ^1^H NMR (300 MHz, DMSO-*d*_6_) *δ* 1.85 (s, 6H, 2 × CH_3_), 10.29 (brs, 2H, 2 × NH) ppm; ^13^C NMR (75 MHz, DMSO-*d*_6_) *δ* 173.76 (2 × C=O), 22.77 (2 × CH_3_) ppm; anal. calcd for C_4_H_8_N_2_O_2_ (116.12): C 41.37, H 6.94, N 24.13; found: C 41.33, H 6.92, N 24.10.

### 2.3 Molecular modeling

Computer simulation was applied to provide valuable insights into structure–activity relationships (SAR) for optimizing lead compounds. Seventeen synthesized compounds were assessed for their biological activity and drug potential through database screening on ChEMBL^21^ and ADMET profiling (absorption, distribution, metabolism, excretion, and toxicity).^22^ Molecular docking was performed to evaluate the binding affinity and predict potential protein binding sites for the synthesized compounds.^23^ The docking simulations were carried out using the Smina program on the crystal structures of the targets SIRT1 (PDB ID: 4ZZH), TPMT (PDB ID: 2BZG), and tyrosinase (PDB ID: 7RK7), obtained from the RCSB Protein Data Bank. Missing residues in the 7RK7 structure were predicted using the AlphaFold web server, employing a homology modeling approach.^24^ Prior to docking, bound water molecules and ligands were removed, and polar hydrogens were added to the proteins. The synthesized compounds were prepared by constructing their 3D structures using ChemDraw Pro 23.1.1.3, followed by energy minimization.^25^ During docking, the ligand molecules were treated as flexible, with binding poses and torsions sampled using the biased probability Monte Carlo minimization technique, which combines random conformational changes with local energy optimization.^26^ The best binding modes were selected based on the lowest energy conformations calculated by the Smina scoring function. Docking results were analyzed and visualized using BIOVIA Discovery Studio Client 2024. Forecasting ADMET properties is a critical yet complex step in the optimization of lead compounds during the drug discovery process. To evaluate these properties for the synthesized compounds, predictions were carried out using SwissADME and DataWarrior software tools.^27^

## 3 Results and discussion

### 3.1 Chemistry

**Scheme 1:**
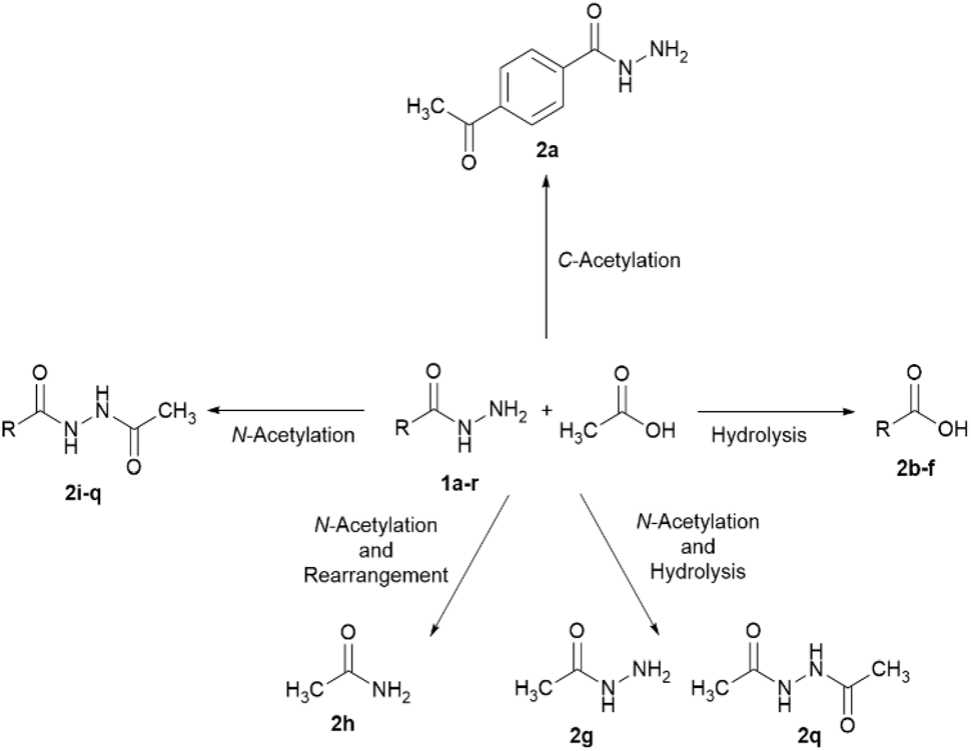
Various interactions of acetic acid with alkyl/aryl/heteroaryl hydrazides.

Acetic acid is an organic acid primarily derived from natural sources. It is considered as a green compound due to its properties, such as high solubility in water, biodegradability, biocompatibility, and non-toxic nature. The acetate anion, the conjugate base of acetic acid, serves as a building block in the biosynthesis of various macronutrients. In this study, acetic acid was used as an environmentally friendly substrate to react with hydrazides under reflux conditions (Scheme 1 and Table 1).

**Table 1:** Products formed in the reaction of hydrazides 1a-r and acetic acid

Entry	R	Product	Time, h	Yield, %	m.p. (lit.), °C
1	C_6_H_5_	2a	3	83.1	214–215 (206–208)^28^
2	4-*t*-Bu-C_6_H_4_	2b	8	72.4	168–169 (162–165) [Sigma-Aldrich]
3	3-CH_3_O-C_6_H_4_	2c	6	73.6	115–116 (105–107) [Sigma-Aldrich]
4	2,4-(HO)_2_–C_6_H_3_	2d	10	84.5	204–206 (213–217) [Sigma-Aldrich]
5	Pyridine-4-yl	2e	4	71.9	>300 (315–319) [Sigma-Aldrich]
6	Pyridine-3-yl	2f	7	98.2	228–230 (234–238) [Sigma-Aldrich]
7	4-(CH_3_)_2_N-C_6_H_4_	2g	9	77.6	71–73 (58–68) [Sigma-Aldrich]
8	H	2h	9	95.6	84–85 (78–80) [Sigma-Aldrich]
9	4-O_2_N-C_6_H_4_	2i	3	91.9	225–227 (240–241)^29^
10	4-F-C_6_H_4_	2j	10	81.1	196–198 (192–194)^30^
11	4-F_3_C-C_6_H_4_	2k	10	77.3	259–261 (Not reported)^31^
12	4-HO-C_6_H_4_	2l	2.5	74.2	252–253 (250)^32^
13	3-HO-C_6_H_4_	2m	3	75.4	198–199 (208)^32^
14	3-Br-C_6_H_4_	2n	6.5	81.3	150–151 (169)^33^
15	3-HO-naphthalen-2-yl	2o	8	90.9	246–248 (234–235)^34^
16	Thiophen-2yl	2p	10	79.7	163–165 (178–179)^30^
17	CH_3_	2q	9	81.8	135–136 (129–130)^35^
18	H_5_C_6_-CH_2_	2q	10	75.2	135–136 (129–130)^35^

As shown in Scheme 1, hydrazide derivatives 1a–r reacted differently with acetic acid. *C*-acetylation was observed only in hydrazide 1a. Benzohydrazide (1a) underwent acetylation at the para position, despite the electron-withdrawing and meta-directing nature of the –CONHNH_2_ group. Product 2a had been previously synthesized *via* the reaction of ethyl 4-acetylbenzoate with hydrazine hydrate in 73% yield.^28^ Hydrazides 1b–f were hydrolyzed to the corresponding carboxylic acids 2b–f in the presence of acetic acid.

**Scheme 2:**
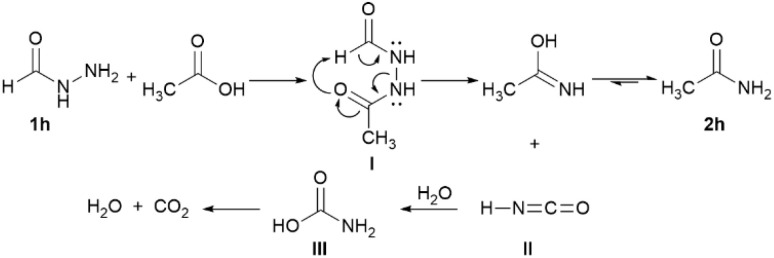
Proposed mechanism for the formation of acetamide from formic hydrazide.

4-(Dimethylamino)benzohydrazide (1g) and formic hydrazide (1h) were initially *N*-acetylated. Product 2g was obtained through the hydrolysis of its *N*-acetylated intermediate. *N*′-Formylacetohydrazide (I) underwent rearrangement to acetamide (2h) and isocyanic acid (II), which was then hydrolyzed to carbamic acid (III) and decomposed into ammonia and carbon dioxide (Scheme 2).

**Scheme 3:**
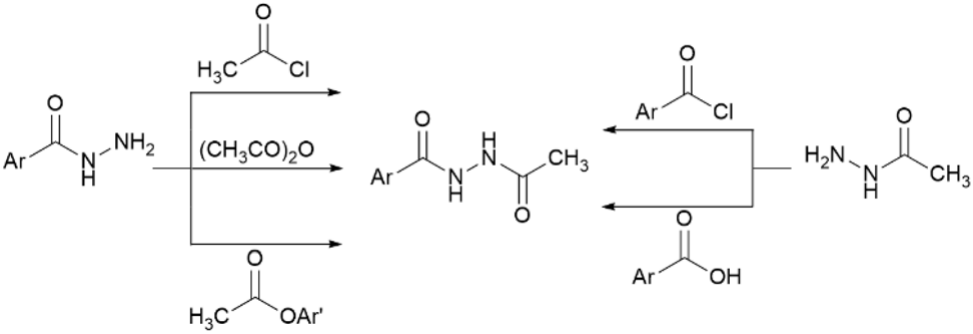
Synthetic routes of *N*′-acetyl hydrazide derivatives.

Hydrazides 1i–q were acetylated on their NH_2_ groups to yield *N*′-acetyl hydrazides 2i–q. These products have been prepared *via N*-acetylation of aryl hydrazides using acetylating reagents such as acetyl chloride, acetic anhydride, and acetyl arenoates, as well as *N*-acetylation of acetohydrazide using aroyl chlorides or aryl carboxylic acids (Scheme 3).^29–33,35–37^

**Scheme 4:**
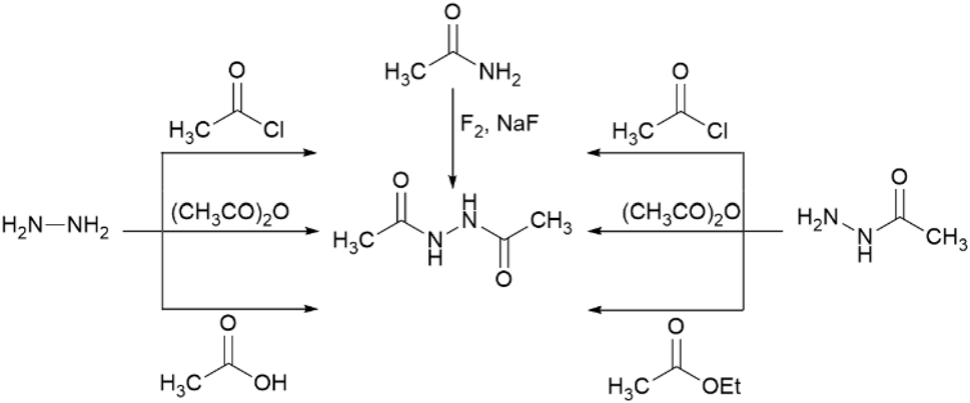
Selected synthetic routes of *N*′-acetylacetohydrazide.

*N*'-Acetylacetohydrazide (2q) was produced *via* the initial *N*-acetylation of both acetohydrazide (1q) and phenylacetic hydrazide (1r). Subsequent hydrolysis of the *N*-acetylated intermediate of hydrazide 1r and re-acetylation afforded product 2q. This product has been primarily synthesized *via* three routes (Scheme 4): (A) the reaction of hydrazine hydrate with common acetylating agents;^35^ (B) *N*-acetylation of acetohydrazide;^38^ (C) dehydrogenative N–N coupling of acetamide.^39^

The chemical structures of products 2a–q were confirmed by physical and spectral data. In ^1^H NMR spectroscopy, a doublet of doublets at *δ* 7.71 and 7.90 ppm defined the *para*-substitution pattern of product 2a. Singlet or broad peaks in the range of 10.44–13.02 ppm corresponded to the hydroxyl group of carboxylic acids 2b–f. Surprisingly, the 2-hydroxyl group of the aryl ring in hydrazide 1d was removed in the final product 2d. Two separate signals at 7.47 and 9.79 ppm were attributed to the NH_2_ and NH protons of hydrazide 2g, respectively, while in compound 2h, a peak at 12.25 ppm corresponded to the amidic NH_2_ protons. In *N*-acetylated products 2i–q, protons of the two NH groups appeared at 9.76–10.82 ppm. Additionally, peaks at 164.53–173.76 ppm were assigned to the carbons of their amidic carbonyl groups.

### 3.2 Molecular modeling analysis

#### 3.2.1 Database screening and molecular docking analysis

The synthesized compounds were screened in ChEMBL, a manually curated database of bioactive molecules with drug-like properties, to identify any known interactions with the targets. This initial analysis provided a broader chemical and pharmacological context for the compounds. Subsequently, all synthesized compounds were subjected to molecular docking against the identified targets. Among them, compounds 2b (ChEMBL93746), 2c (ChEMBL22425), and 2d (ChEMBL441343) demonstrated significant activity, with 2b exhibiting an IC_50_ of 200 μM against sirtuin 1 (SIRT1), 2c showing an IC_50_ of 199 μM against thiopurine methyltransferase (TPMT), and 2d achieving an IC_50_ of 9.3 μM against tyrosinase. SIRT1 is a nicotinamide adenine dinucleotide (NAD^+^)-dependent deacetylase that regulates critical cellular processes, including metabolism, stress response, and aging.^40^ TPMT catalyzes the methylation of thiopurine drugs, which are widely used to treat autoimmune diseases, certain cancers, and to prevent organ transplant rejection.^41^ Tyrosinase, a copper-containing enzyme, is essential for melanin biosynthesis, determining the pigment of skin, hair, and eyes, by catalyzing the oxidation of tyrosine into melanin precursors.^42^ Additionally, compounds 2e (ChEMBL1203), 2f (ChEMBL573), 2g (ChEMBL3091859), and 2h (ChEMBL16081) were found in the ChEMBL database but lack reported activity and specific targets. Notably, compound 2f (ChEMBL573), identified as niacin or vitamin B3, is an approved drug. Due to the structural similarities and the shared synthesis methodology, all synthesized compounds were docked against the three target proteins: SIRT1, TPMT, and Tyrosinase. Molecular docking aimed to predict the most favorable binding poses of the synthesized compounds within the active sites of the target proteins and identify key residues and binding modes. This approach enabled the identification of protein target candidates associated with the docking poses of the synthesized compounds, which were ranked based on their predicted affinity (see Table 2). Based on Table 2, the predicted affinities indicated that some compounds exhibited more favorable affinities compared to their corresponding compounds reported in the ChEMBL database. For instance, the predicted affinity value of compound 2b with SIRT1 was −7.16 kcal mol^−1^, while compounds 2o and 2k demonstrated more favorable energy values. Similarly, TPMT exhibited an affinity of −7.02 kcal mol^−1^ with compound 2c, whereas the nine compounds listed in the table (2o, 2k, 2m, 2a, 2n, 2j, 2b, 2l, and 2i) demonstrated even more favorable binding energy values in comparison to 2c. Additionally, tyrosinase showed better affinities with compounds 2o, 2l, and 2m compared to compound 2d (−6.47 kcal mol^−1^). Notably, compound 2o emerged as particularly interesting, as it demonstrated the most favorable affinity across all targets. This suggested its potential as a promising multi-target drug candidate for further investigation.^43–49^

**Table 2:** The docking-predicted minimized affinity of the products for three targets: SIRT1, TPMT and tyrosinase

Product	Affinity of SIRT1 (kcal mol^−1^)	Product	Affinity of TPMT (kcal mol^−1^)	Product	Affinity of tyrosinase (kcal mol^−1^)
2o	−8.59	2o	−9.62	2o	−8.33
2k	−7.76	2k	−8.95	2l	−7.29
2b	−7.16	2m	−8.4	2m	−7.28
2n	−7.04	2a	−8.35	2d	−6.47
2a	−6.97	2n	−8.15	2p	−6.32
2i	−6.82	2j	−8.12	2j	−6.16
2m	−6.77	2b	−8.07	2a	−5.96
2j	−6.7	2l	−8.02	2k	−5.79
2l	−6.44	2i	−7.66	2b	−5.79
2d	−5.91	2c	−7.02	2n	−5.6
2c	−5.67	2d	−6.78	2f	−5.51
2p	−5.48	2p	−6.72	2i	−5.38
2f	−5.26	2f	−6.36	2c	−5.12
2e	−5.13	2e	−6.1	2e	−4.68
2g	−4.08	2q	−5.19	2g	−4.27
2q	−4.06	2g	−4.23	2q	−3.73
2h	−3.15	2h	−3.52	2h	−3.2

**Fig. 1:**
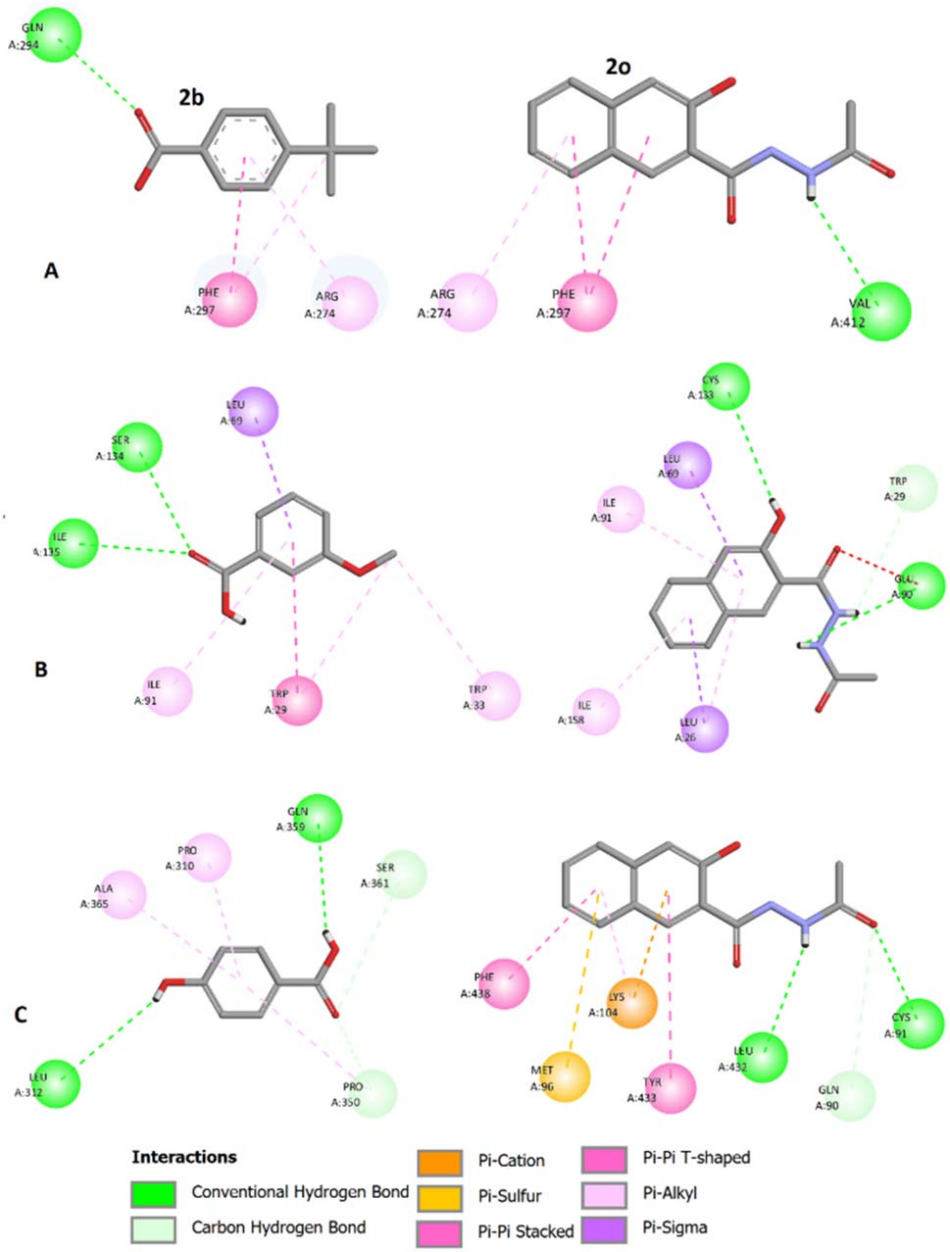
Molecular docking analysis illustrating the interactions of (A) compounds 2b and 2o with SIRT1, (B) compounds 2c and 2o with TPMT, and (C) compounds 2d and 2o with tyrosinase. The images were generated using Discovery Studio 2024 Client.

The two-dimensional (2D) ligand–protein interactions of compounds 2b and 2o with SIRT1, 2c and 2o with TPMT, and 2d and 2o with tyrosinase are shown in Fig. 1. In Fig. 1, green dots represent hydrogen bonds, while orange, pink, and purple dots represent hydrophobic interactions. Key residue interactions for each protein with the ligand have been identified and summarized in Table 3. As shown in the docking results (Fig. 1 and Table 3), the biological activity of the compounds appeared to be influenced by several key structural elements that interact with residues. The naphthalene ring system provides a rigid aromatic scaffold that has π–π stacking interactions with aromatic residues in the target. Substituted naphthol or phenolic hydroxyl groups contribute to hydrogen bonding and increase polarity, potentially improving aqueous solubility and binding affinity. Hydrazide groups are capable of acting as hydrogen bond donors and acceptors, thereby facilitating interactions with polar residues at the binding site. Generally, compound 2o exhibited more interactions with each specific target compared to its corresponding compounds reported in the ChEMBL database.^50–56^

**Table 3:** Interaction of each protein with the ligands

Complex	Hydrogen bonds	Hydrophobic interactions
SIRT1-2b	GLN294	PHE297, ARG274
SIRT1-2o	VAL412	PHE297, ARG274
TPMT-2c	SER134, ILE135	TRP29, TRP33, ILU69, ILU91
TPMT-2o	GLU90, CYS133, TRP29	LEU26, LEU69, ILU91, ILU158
Tyrosinase-2d	LEU312, PRO350, GLN359, SER361	PRO310, ALA365
Tyrosinase-2o	GLN90, CYS91, LEU432	MET96, LYS104, TYR433, PHE438

#### 3.2.2 ADMET predictions

The use of ADMET analysis facilitates the prediction of the action and behavior of newly synthesized compounds, reducing both costs and time during drug development. The bioavailability radar in SwissADME offers an effective visual tool for assessing drug-likeness by displaying six key descriptive properties at a glance. These properties include lipophilicity (LIPO), molecular size (SIZE), polarity (POLAR), solubility (INSOLU), saturation (INSATU), and molecular flexibility (FLEX). Each descriptor has an optimal range, and molecules that fall within the pink region of the radar are considered to exhibit favourable bioavailability properties in the body. The pink region represents the ideal range for each property as follows: lipophilicity (XLOGP3) between −0.7 and +5.0, molecular weight (MW) between 150 and 500 g mol^−1^, topological polar surface area (TPSA) between 20 and 130 Å^2^, solubility (log S) no higher than 6, saturation (fraction of carbons in sp^3^ hybridisation) not less than 0.25, and flexibility with no more than nine rotatable bonds. Table 4 illustrates the bioavailability radar for the synthesized compounds. An analysis of these radars highlights the unsaturation characteristics of the majority of the compounds. When the unsaturation value of a compound exceeds the boundary of the pink region in the radar, it indicates that the degree of unsaturation is beyond the acceptable range. Consequently, the INSATU parameter will require optimization. This deviation could adversely affect key properties such as solubility, stability, and bioavailability. The toxicity risk of the synthesized compounds was assessed using Data Warrior, a widely used computational tool for predicting potential adverse effects of chemical compounds. This evaluation focused on identifying the likelihood of four specific toxicological risks: mutagenicity, tumorigenicity, irritancy, and reproductive toxicity. Mutagenicity indicates the potential of a compound to induce genetic mutations, which could lead to serious health implications, including cancer. Tumorigenicity refers to the compound's potential to promote tumor formation. Irritancy evaluates the potential of the compound to cause irritation upon contact with biological tissues, such as skin or mucous membranes. Lastly, reproductive toxicity assesses the potential of the compound to negatively impact reproductive health, including effects on fertility or embryonic development. The results in Table 5 indicate that only compounds 2h and 2g exhibit a high risk for some of the evaluated toxicity parameters, which will be further investigated.

**Table 4:** Bioavailability radar for the synthesized compounds: a visual overview of drug-likeness with optimal ranges highlighted in pink

Product	Radar graph	Product	Radar graph
2a	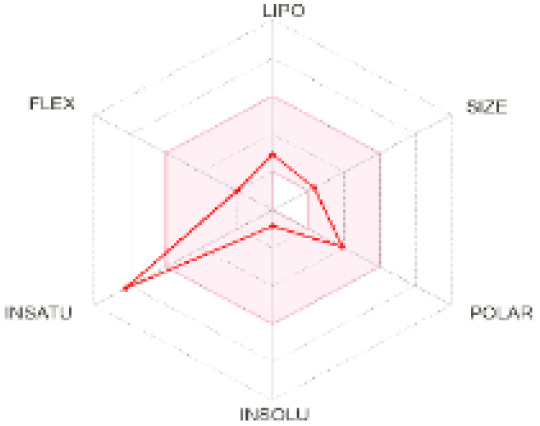	2j	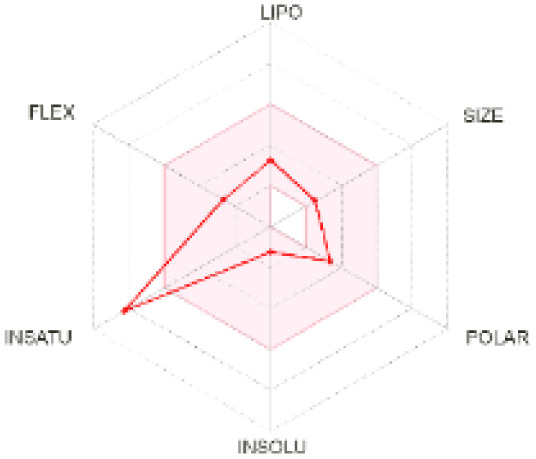
2b	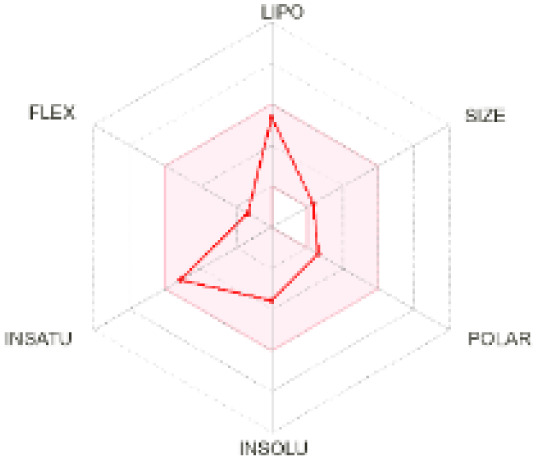	2k	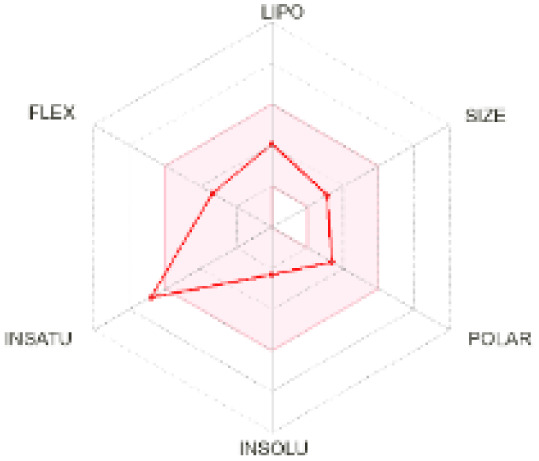
2c	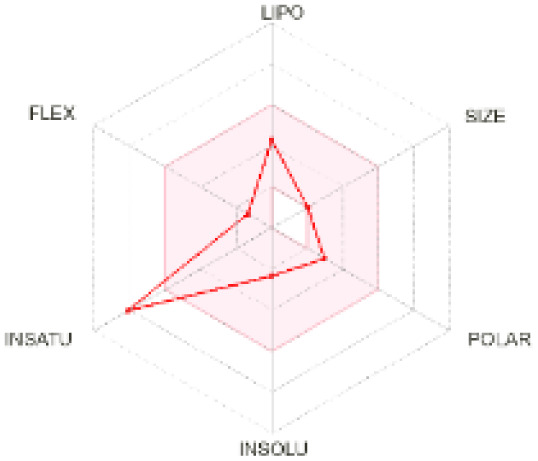	2l	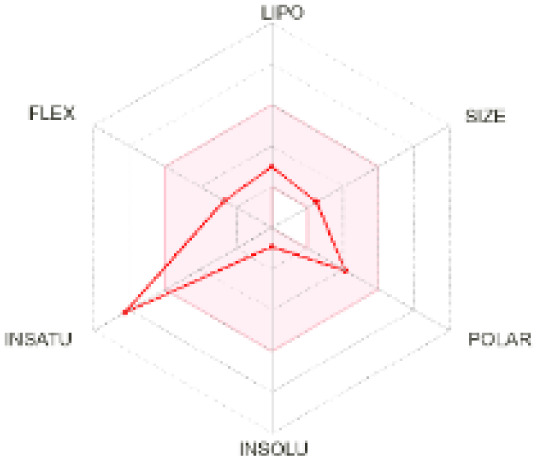
2d	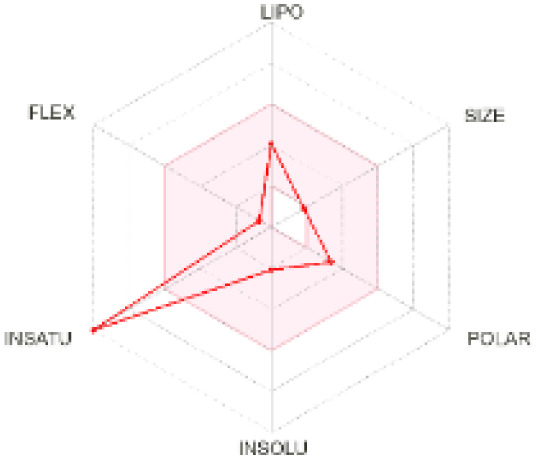	2m	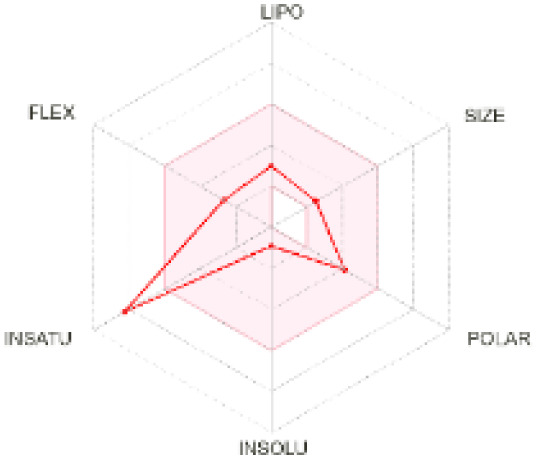
2e	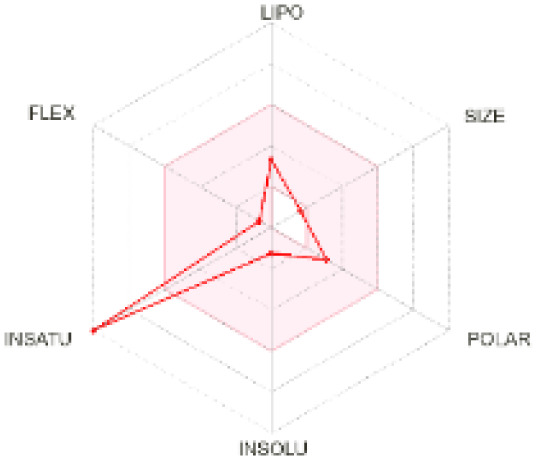	2n	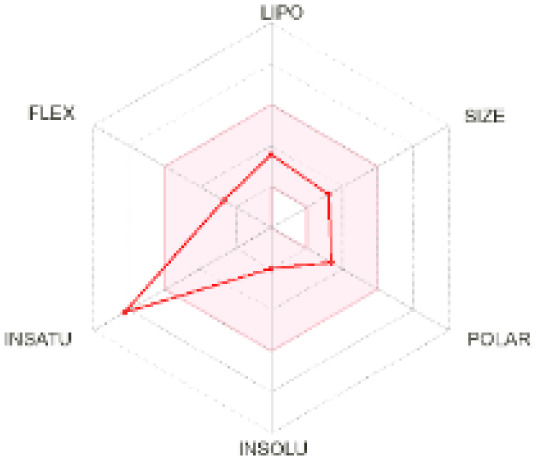
2f	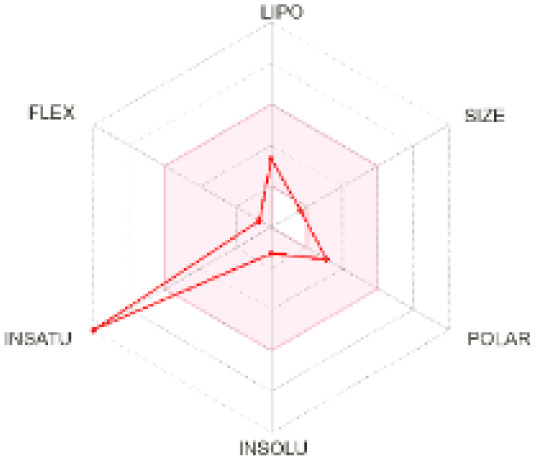	2o	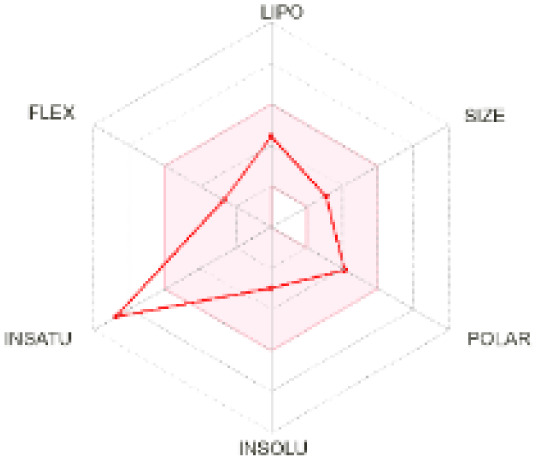
2g	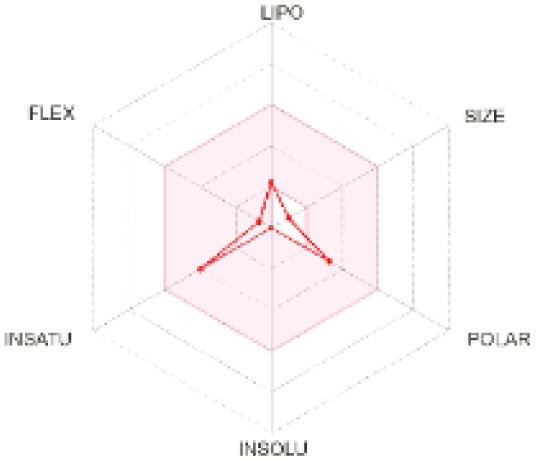	2p	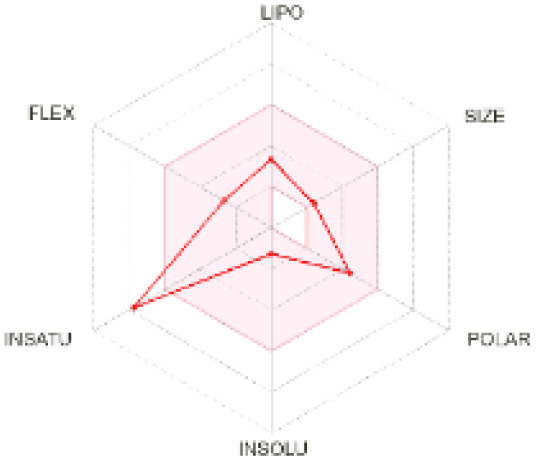
2h	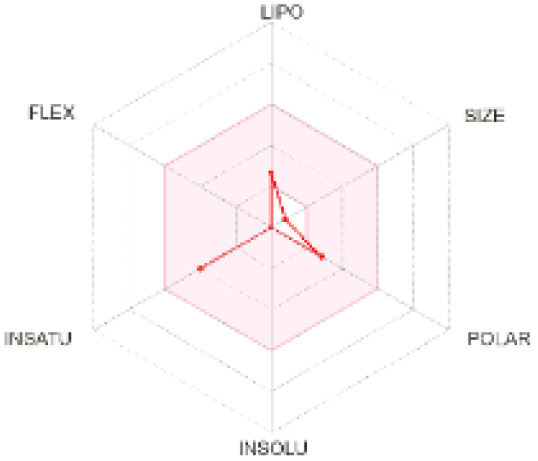	2q	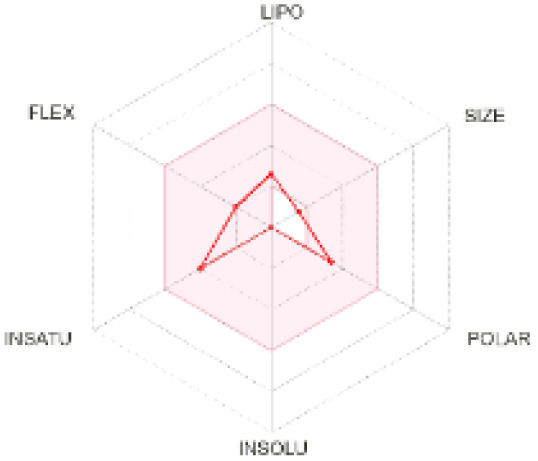
2i	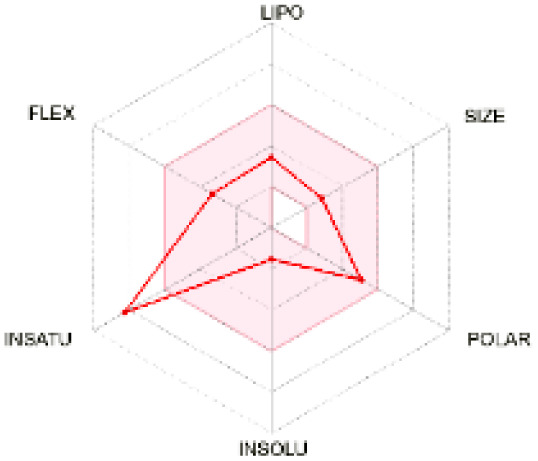		

**Table 5:** Toxicity testing for the synthesized compounds

Product	Mutagenic	Tumorigenic	Reproductive effective	Irritant
2a	None	Low	None	None
2b	None	None	Low	Low
2c	None	None	None	None
2d	High	None	None	None
2e	None	None	None	None
2f	None	None	None	None
2g	High	High	None	None
2h	High	High	High	None
2i	None	None	Low	None
2j	None	None	Low	None
2k	None	None	Low	None
2l	None	None	Low	None
2m	None	None	Low	None
2n	None	None	Low	None
2o	None	None	Low	None
2p	None	None	Low	None
2q	None	None	Low	None

## 4 Conclusions

Acetic acid is a non-toxic, low cost, water-soluble, accessible, eco-friendly, and biodegradable compound with a variety of applications. It was used as a green solvent in the reactions and recrystallization processes. Small amounts of acid have been shown to facilitate the reaction progress. Despite its lower reactivity, it is safer and cheaper than other acetylating agents such as acetyl chloride, acetic anhydride, and ethyl acetate. In this research, a series of hydrazides reacted with acetic acid under reflux. Acetic acid, in addition to its role as a reaction and recrystallization solvent, acted as an acetylating agent and catalyst. It easily *N*-acetylated hydrazides 1i–r without the need for carbodiimides. Several unexpected reactions were observed. The novel method offered for preparing products 2a and 2i–q occurred under safer, greener, and cheaper conditions compared to previous methods. Molecular modeling and ChEMBL database screening showed that several newly synthesized compounds had promising inhibitory activity against SIRT1, TPMT, and tyrosinase. Among them, compound 2o stood out with its strong multi-target affinity. The valuable results obtained from this research encourage us to investigate the interaction of equivalent reagents with acetic acid in the future. In addition, molecular modeling findings suggest that further development of the synthesized compounds, especially 2o, could lead to promising multi-target drug candidates.

## Data availability

The datasets generated during and/or analyzed during the current study are available from the corresponding authors upon reasonable request.

## Author contributions

Hamid Beyzaei: supervision, formal analysis, writing – original draft, writing – review & editing, funding acquisition; Sakineh Sheikh: methodology, investigation; Fereshteh Shiri: supervision, writing – review & editing, conceptualization, software; Reza Aryan: investigation, data curation.

## Conflicts of interest

There are no conflicts to declare.

## Supplementary Material

RA-015-D5RA01286D-s001
