# Supplementary material for: Evaluation of the interactions of hydrazide derivatives with acetic acid and molecular modeling analysis of N-acetylated hydrazides
Source: RSC Adv. 2025 Apr 28;15(17):13618–27. doi: 10.1039/d5ra01286d (PMC12035683; doi:10.1039/d5ra01286d)
Supplement: RA-015-D5RA01286D-s001 [file RA-015-D5RA01286D-s001.pdf]

Supplementary Information (SI) for RSC Advances.
This journal is © The Royal Society of Chemistry 2025

Supporting Information

Evaluation of the interactions of hydrazide derivatives with acetic acid, and molecular modeling analysis of *N*-acetylated hydrazides

Hamid Beyzaei*, Sakineh Sheikh, Fereshteh Shiri*, Reza Aryan

Department of Chemistry, Faculty of Science, University of Zabol, Zabol, Iran

Email: hbeyzaei@uoz.ac.ir, hbeyzaei@yahoo.com; and fereshteh.shiri@gmail.com, fereshteh.shiri@uoz.ac.ir

Table of contents

Entry	Description	Structure	Page
Fig. 1	^{1}H NMR of 4-acetylbenzohydrazide (**2a**)	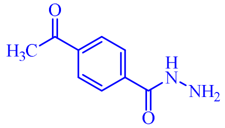	S4
Fig. 2	^{13}C NMR of 4-acetylbenzohydrazide (**2a**)		S5
Fig. 3	FT-IR of 4-acetylbenzohydrazide (**2a**)		S6
Fig. 4	^{1}H NMR of 4-(*tert*-butyl)benzoic acid (**2b**)	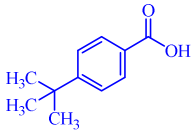	S7
Fig. 5	^{13}C NMR of 4-(*tert*-butyl)benzoic acid (**2b**)		S8
Fig. 6	FT-IR of 4-(*tert*-butyl)benzoic acid (**2b**)		S9
Fig. 7	^{1}H NMR of 3-methoxybenzoic acid (**2c**)	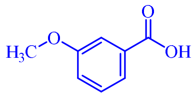	S10
Fig. 8	^{13}C NMR of 3-methoxybenzoic acid (**2c**)		S11
Fig. 9	FT-IR of 3-methoxybenzoic acid (**2c**)		S12
Fig. 10	^{1}H NMR of 4-hydroxybenzoic acid (**2d**)	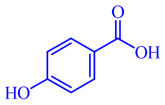	S13
Fig. 11	^{13}C NMR of 4-hydroxybenzoic acid (**2d**)		S14
Fig. 12	FT-IR of 4-hydroxybenzoic acid (**2d**)		S15
Fig. 13	^{1}H NMR of isonicotinic acid (**2e**)	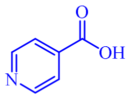	S16
Fig. 14	^{13}C NMR of isonicotinic acid (**2e**)		S17
Fig. 15	FT-IR of isonicotinic acid (**2e**)		S18
Fig. 16	^{1}H NMR of nicotinic acid (**2f**)	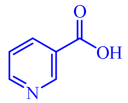	S19
Fig. 17	^{13}C NMR of nicotinic acid (**2f**)		S20
Fig. 18	FT-IR of nicotinic acid (**2f**)		S21
Fig. 19	^{1}H NMR of acetohydrazide (**2g**)	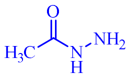	S22
Fig. 20	^{13}C NMR of acetohydrazide (**2g**)		S23
Fig. 21	FT-IR of acetohydrazide (**2g**)		S24
Fig. 22	^{1}H NMR of acetamide (**2h**)	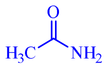	S25
Fig. 23	^{13}C NMR of acetamide (**2h**)		S26
Fig. 24	FT-IR of acetamide (**2h**)		S27

Fig. 25	^{1}H NMR of *N*'-acetyl-4-nitrobenzohydrazide (**2i**)	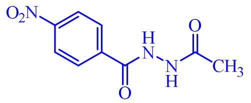	S28
Fig. 26	^{13}C NMR of *N*'-acetyl-4-nitrobenzohydrazide (**2i**)		S29
Fig. 27	FT-IR of *N*'-acetyl-4-nitrobenzohydrazide (**2i**)		S30
Fig. 28	^{1}H NMR of *N*'-acetyl-4-fluorobenzohydrazide (**2j**)	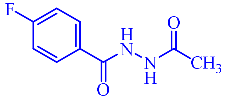	S31
Fig. 29	^{13}C NMR of *N*'-acetyl-4-fluorobenzohydrazide (**2j**)		S32
Fig. 30	FT-IR of *N*'-acetyl-4-fluorobenzohydrazide (**2j**)		S33
Fig. 31	^{1}H NMR of *N*'-acetyl-4-(trifluoromethyl)benzohydrazide (**2k**)	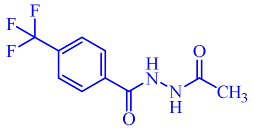	S34
Fig. 32	^{13}C NMR of *N*'-acetyl-4-(trifluoromethyl)benzohydrazide (**2k**)		S35
Fig. 33	FT-IR of *N*'-acetyl-4-(trifluoromethyl)benzohydrazide (**2k**)		S36
Fig. 34	^{1}H NMR of *N*'-acetyl-4-hydroxybenzohydrazide (**2l**)	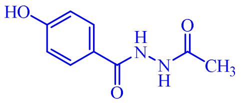	S37
Fig. 35	^{13}C NMR of *N*'-acetyl-4-hydroxybenzohydrazide (**2l**)		S38
Fig. 36	FT-IR of *N*'-acetyl-4-hydroxybenzohydrazide (**2l**)		S39
Fig. 37	^{1}H NMR of *N*'-acetyl-3-hydroxybenzohydrazide (**2m**)	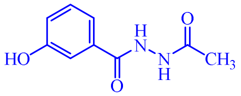	S40
Fig. 38	^{13}C NMR of *N*'-acetyl-3-hydroxybenzohydrazide (**2m**)		S41
Fig. 39	FT-IR of *N*'-acetyl-3-hydroxybenzohydrazide (**2m**)		S42
Fig. 40	^{1}H NMR of *N*'-acetyl-3-bromobenzohydrazide (**2n**)	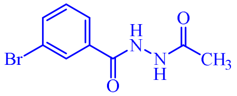	S43
Fig. 41	^{13}C NMR of *N*'-acetyl-3-bromobenzohydrazide (**2n**)		S44
Fig. 42	FT-IR of *N*'-acetyl-3-bromobenzohydrazide (**2n**)		S45
Fig. 43	^{1}H NMR of *N*'-acetyl-3-hydroxy-2-naphthohydrazide (**2o**)	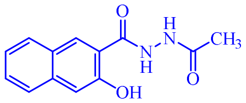	S46
Fig. 44	^{13}C NMR of *N*'-acetyl-3-hydroxy-2-naphthohydrazide (**2o**)		S47
Fig. 45	FT-IR of *N*'-acetyl-3-hydroxy-2-naphthohydrazide (**2o**)		S48
Fig. 46	^{1}H NMR of *N*'-acetylthiophene-2-carbohydrazide (**2p**)	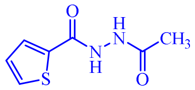	S49
Fig. 47	^{13}C NMR of *N*'-acetylthiophene-2-carbohydrazide (**2p**)		S50
Fig. 48	FT-IR of *N*'-acetylthiophene-2-carbohydrazide (**2p**)		S51
Fig. 49	^{1}H NMR of *N*'-acetylacetohydrazide (**2q**)	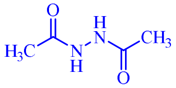	S52
Fig. 50	^{13}C NMR of *N*'-acetylacetohydrazide (**2q**)		S53
Fig. 51	FT-IR of *N*'-acetylacetohydrazide (**2q**)		S54

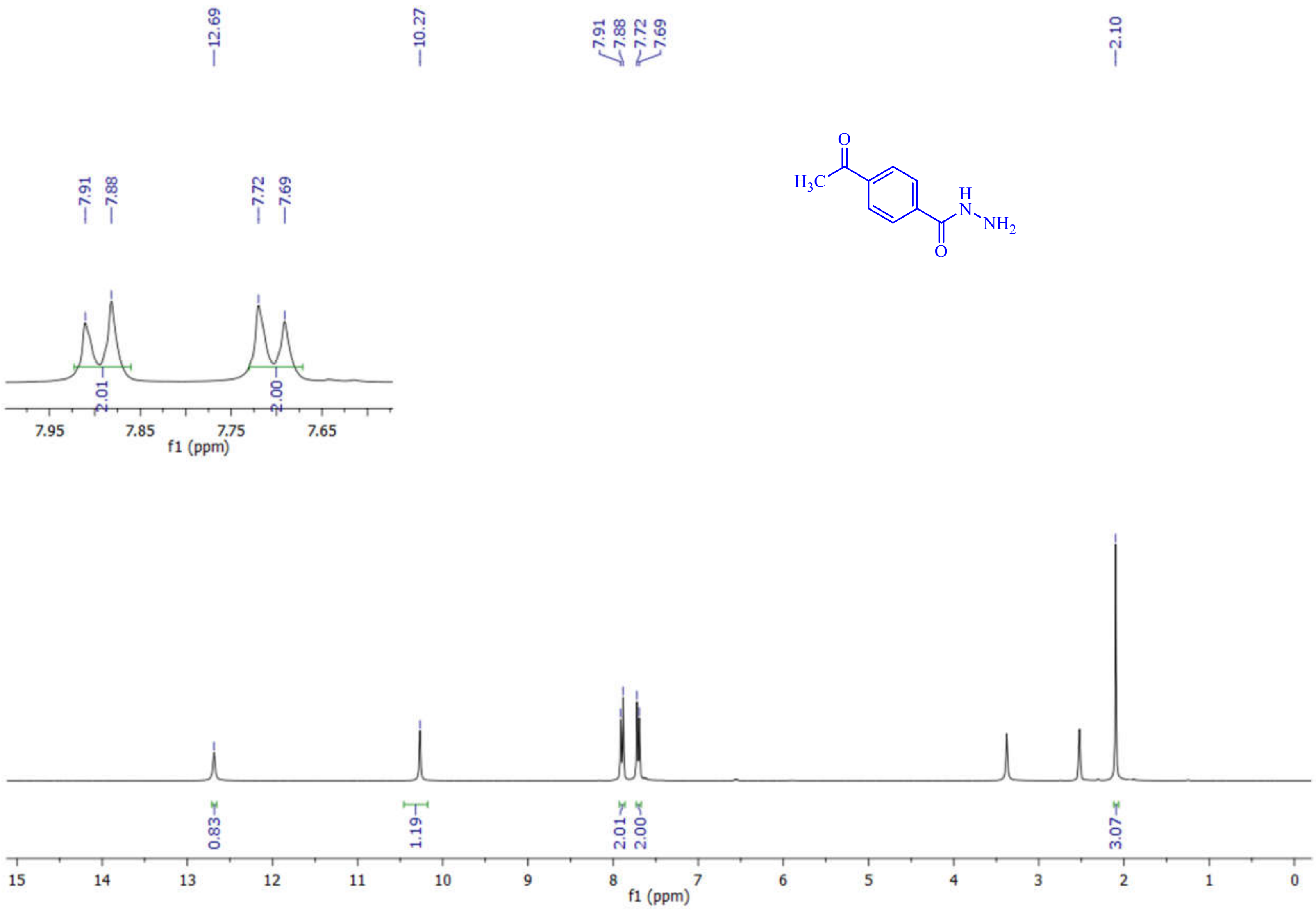

Fig. 1 ^{1}H NMR of 4-acetylbenzohydrazide (**2a**)

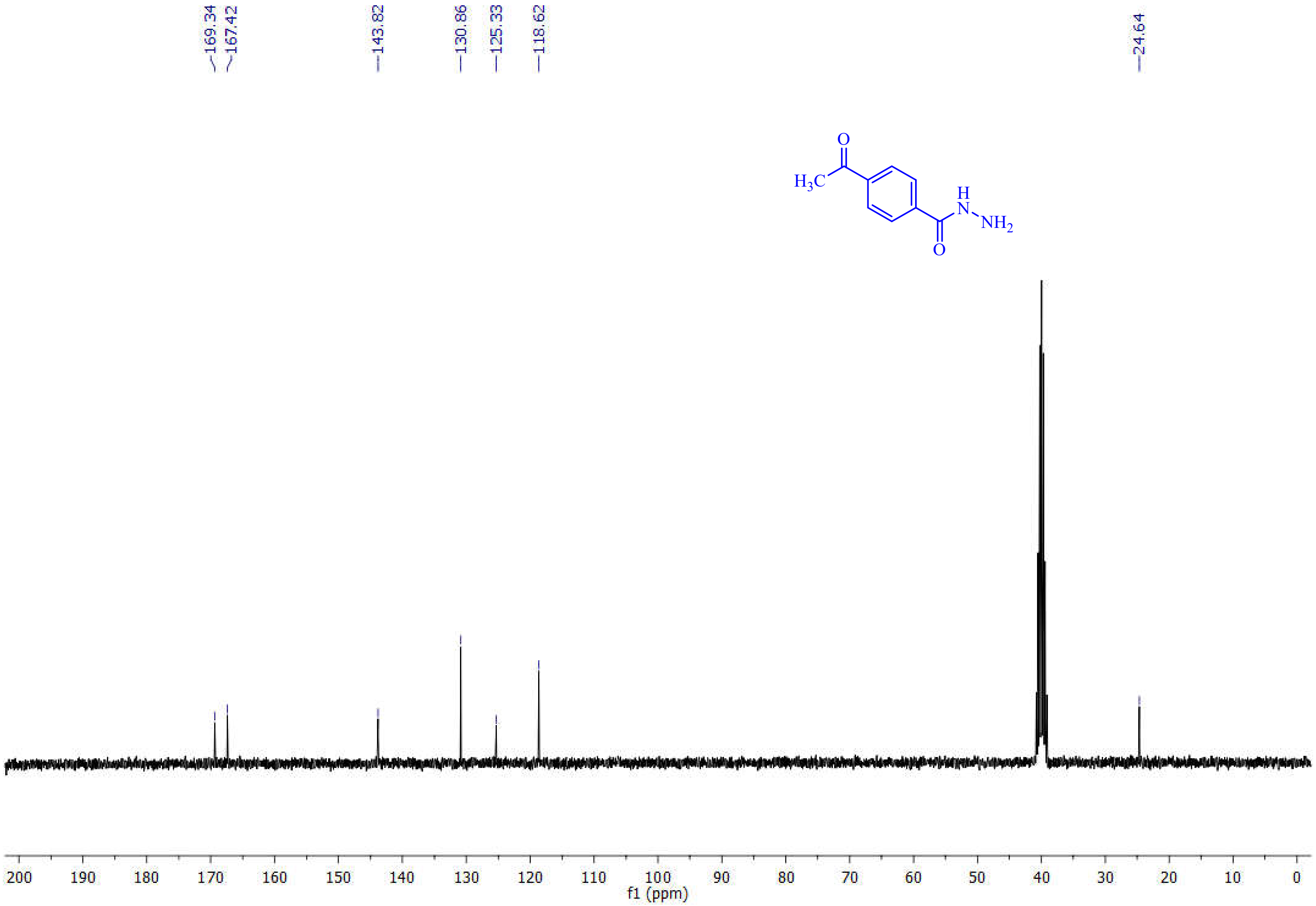

Fig. 2 ^{13}C NMR of 4-acetylbenzohydrazide (**2a**)

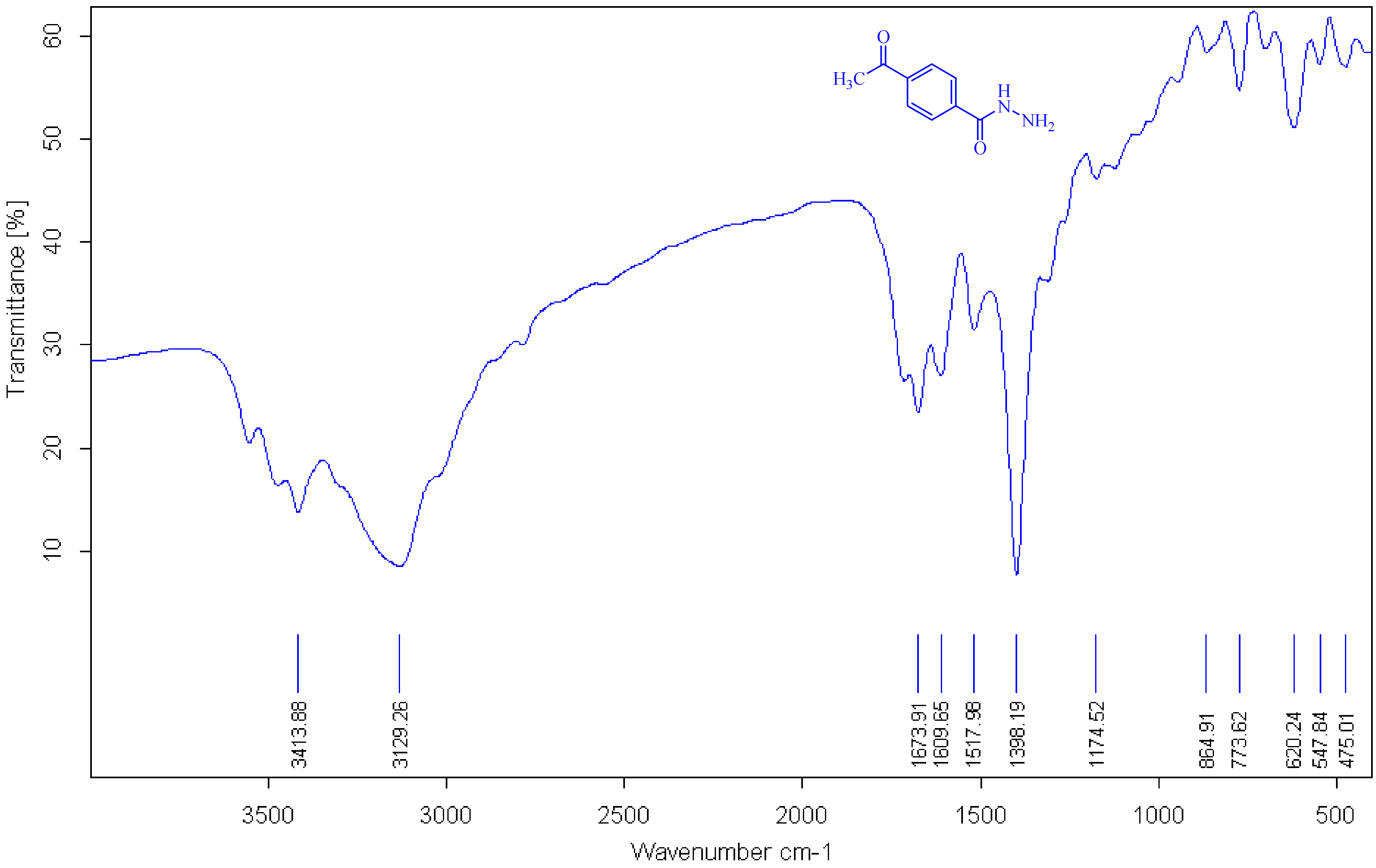

Fig. 3 FT-IR of 4-acetylbenzohydrazide (**2a**)

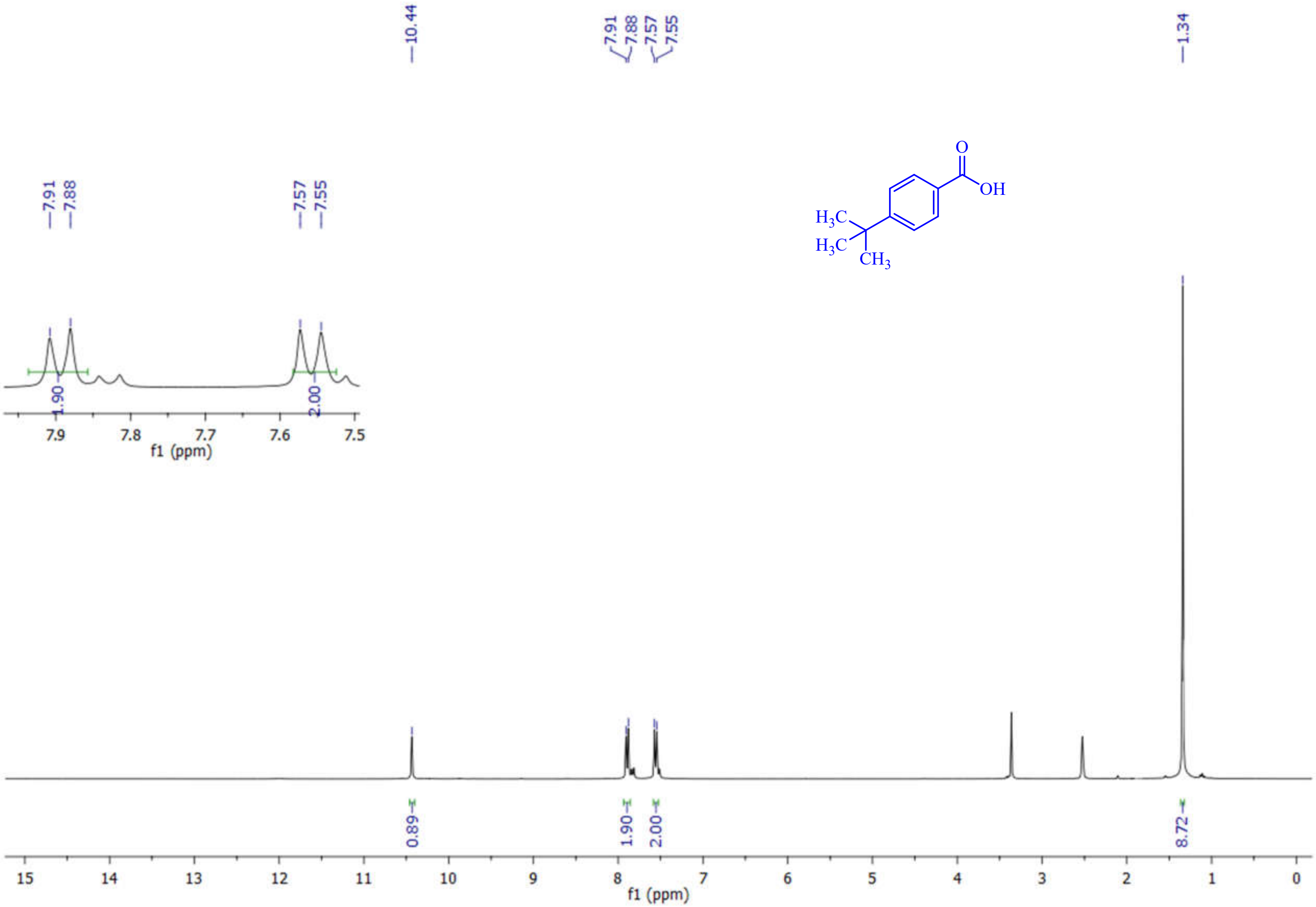

Fig. 4 ^{1}H NMR of 4-(*tert*-butyl)benzoic acid (**2b**)

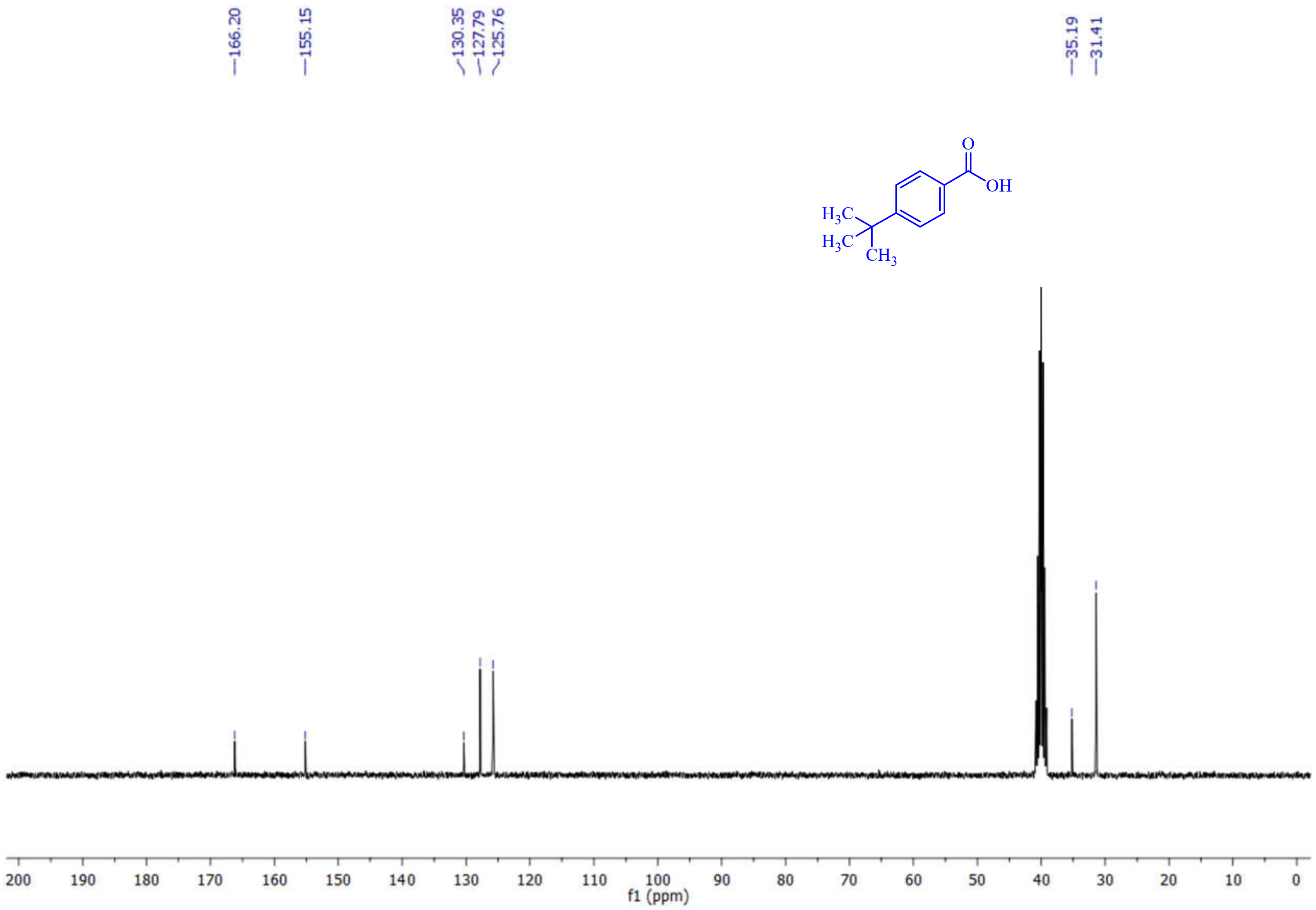

Fig. 5 ^{13}C NMR of 4-(*tert*-butyl)benzoic acid (**2b**)

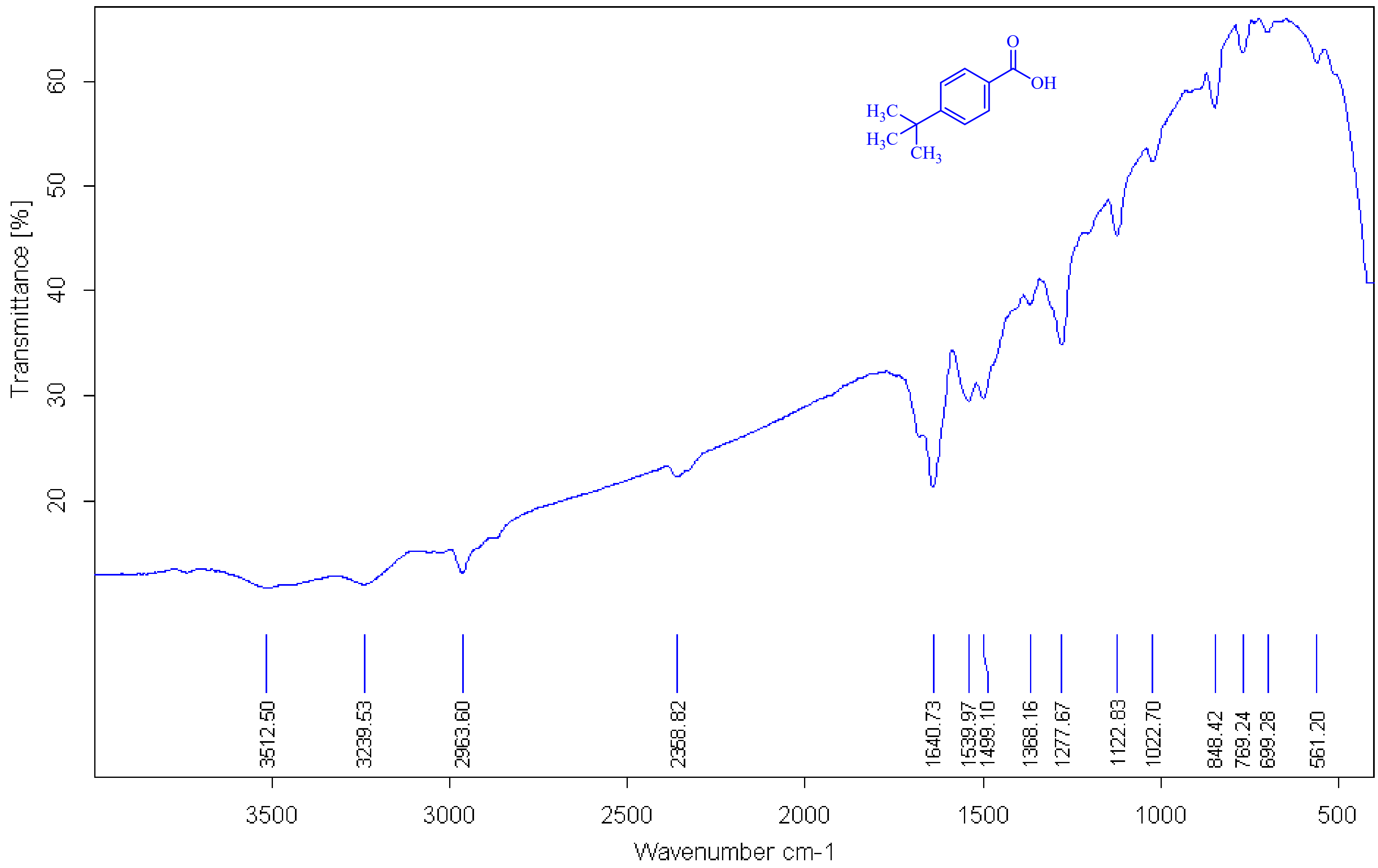

Fig. 6 FT-IR of 4-(*tert*-butyl)benzoic acid (**2b**)

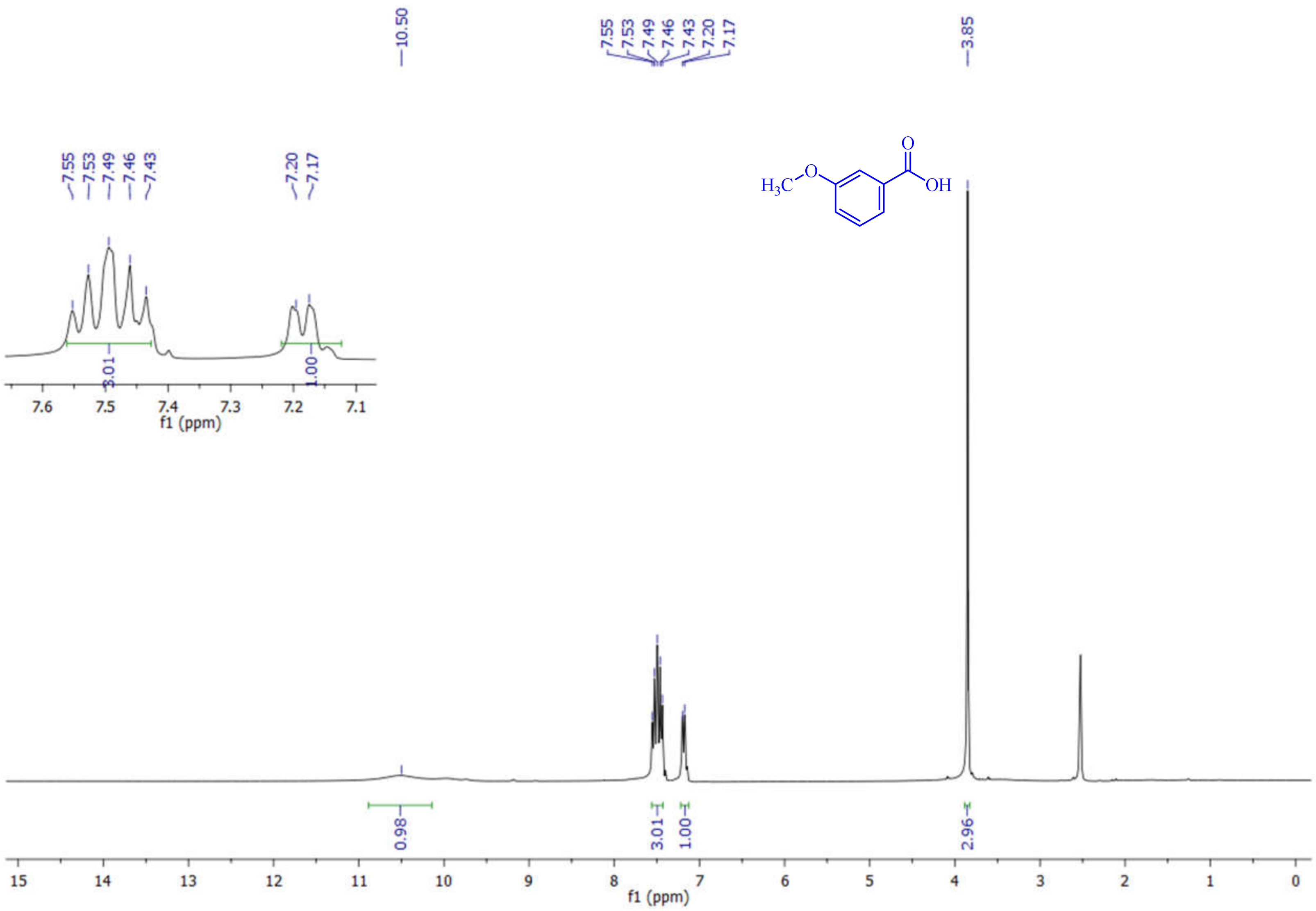

Fig. 7 ^{1}H NMR of 3-methoxybenzoic acid (**2c**)

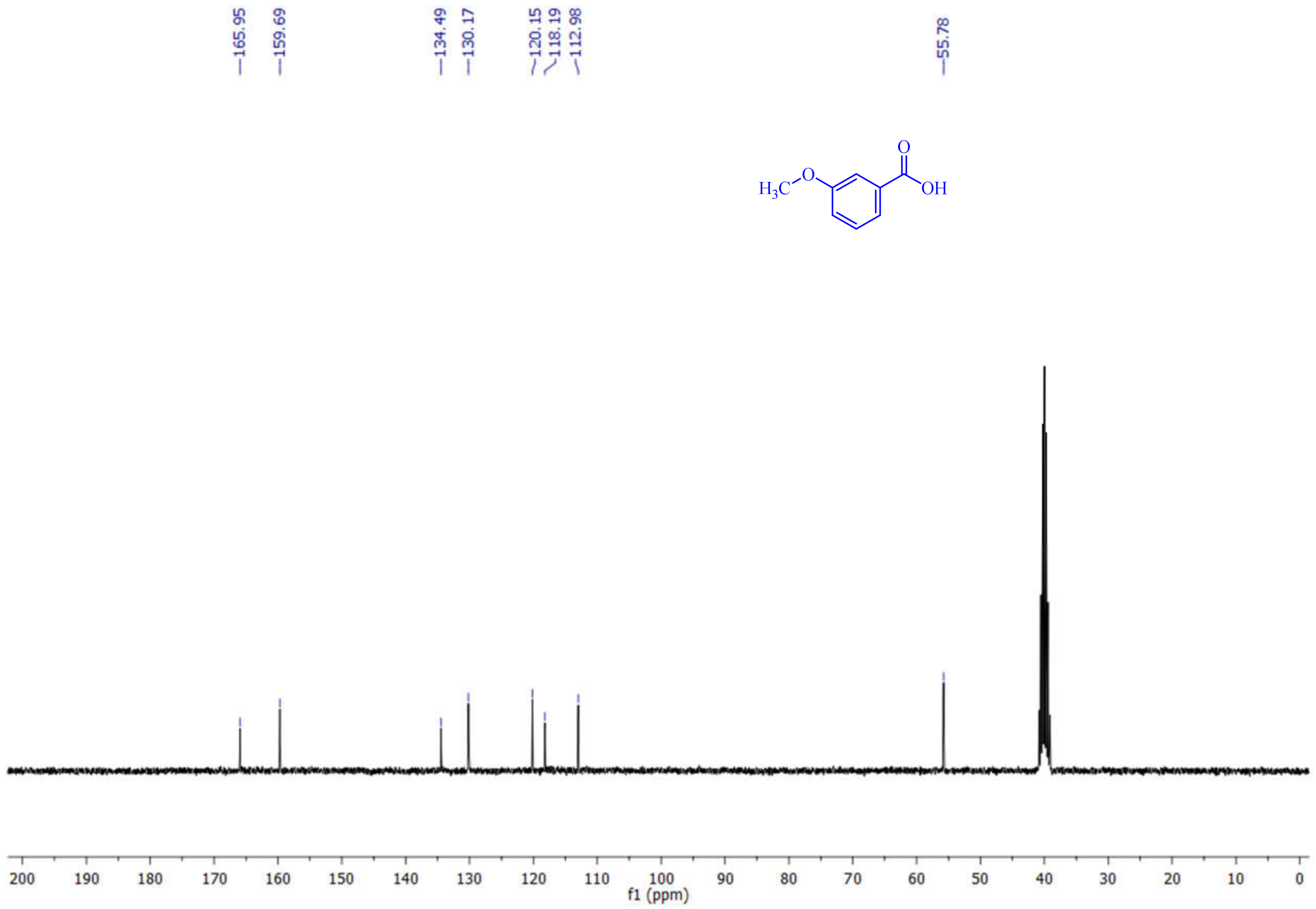

Fig. 8 ^{13}C NMR of 3-methoxybenzoic acid (**2c**)

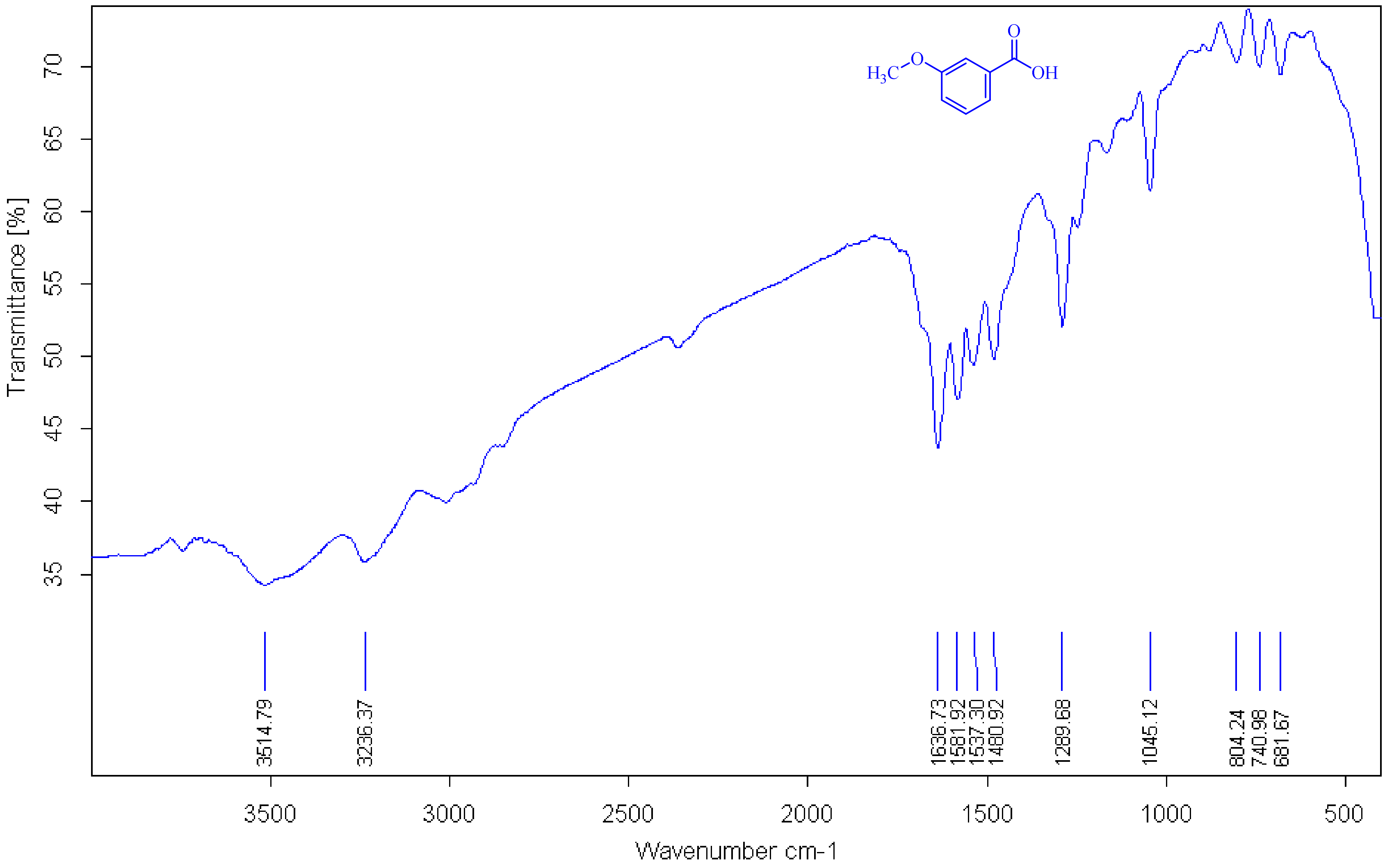

Fig. 9 FT-IR of 3-methoxybenzoic acid (**2c**)

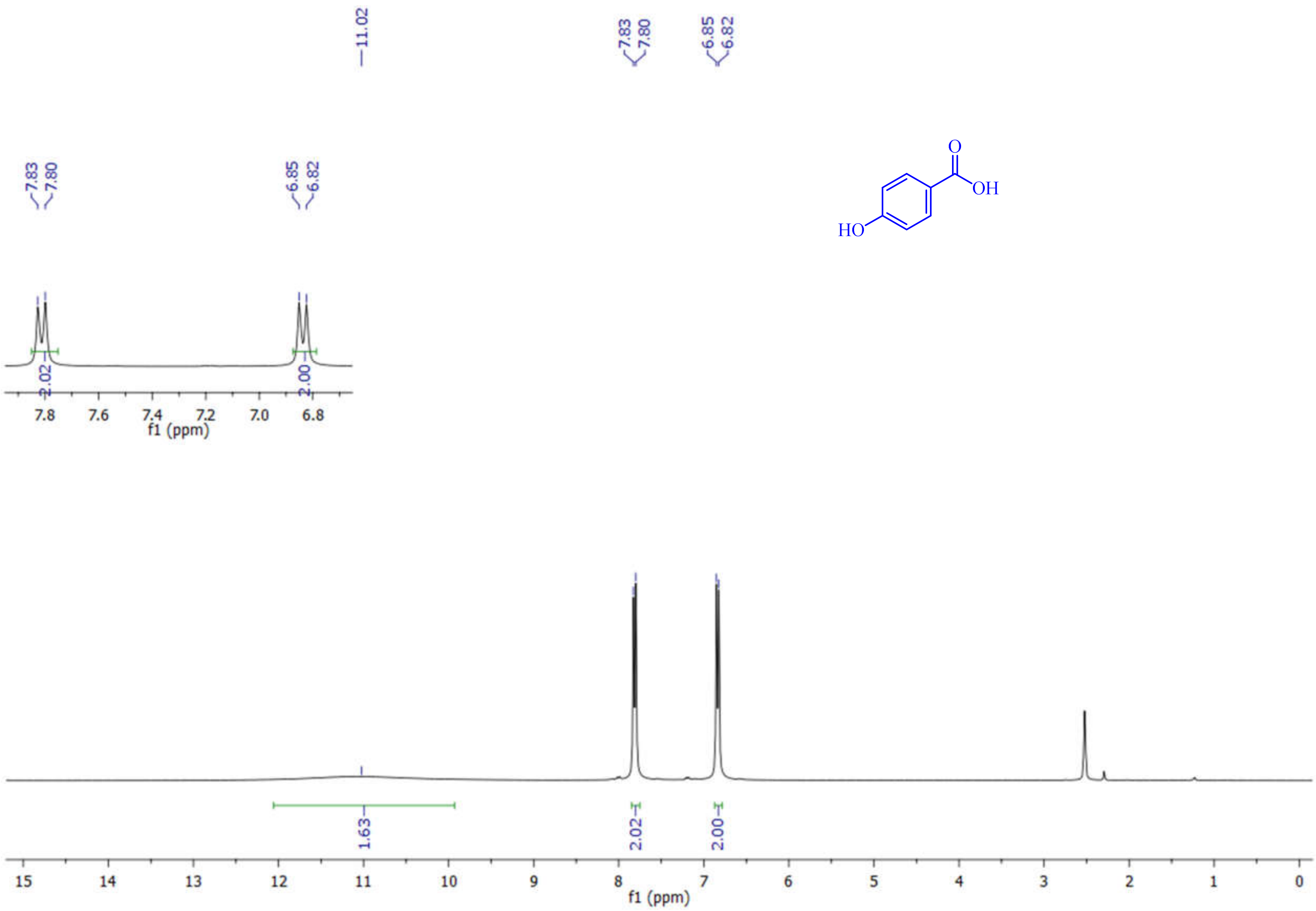

Fig. 10 ^{1}H NMR of 4-hydroxybenzoic acid (**2d**)

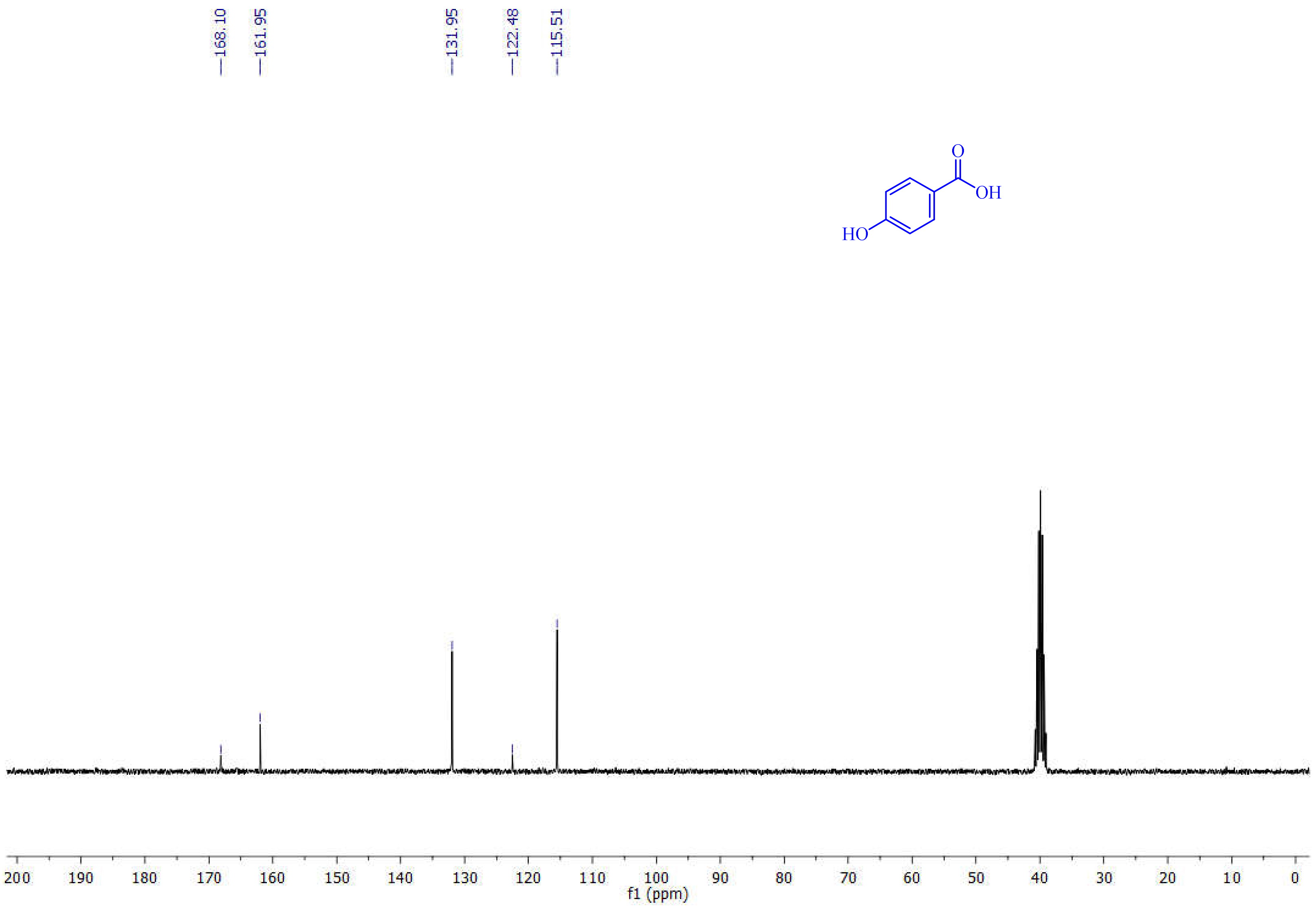

Fig. 11 ^{13}C NMR of 4-hydroxybenzoic acid (**2d**)

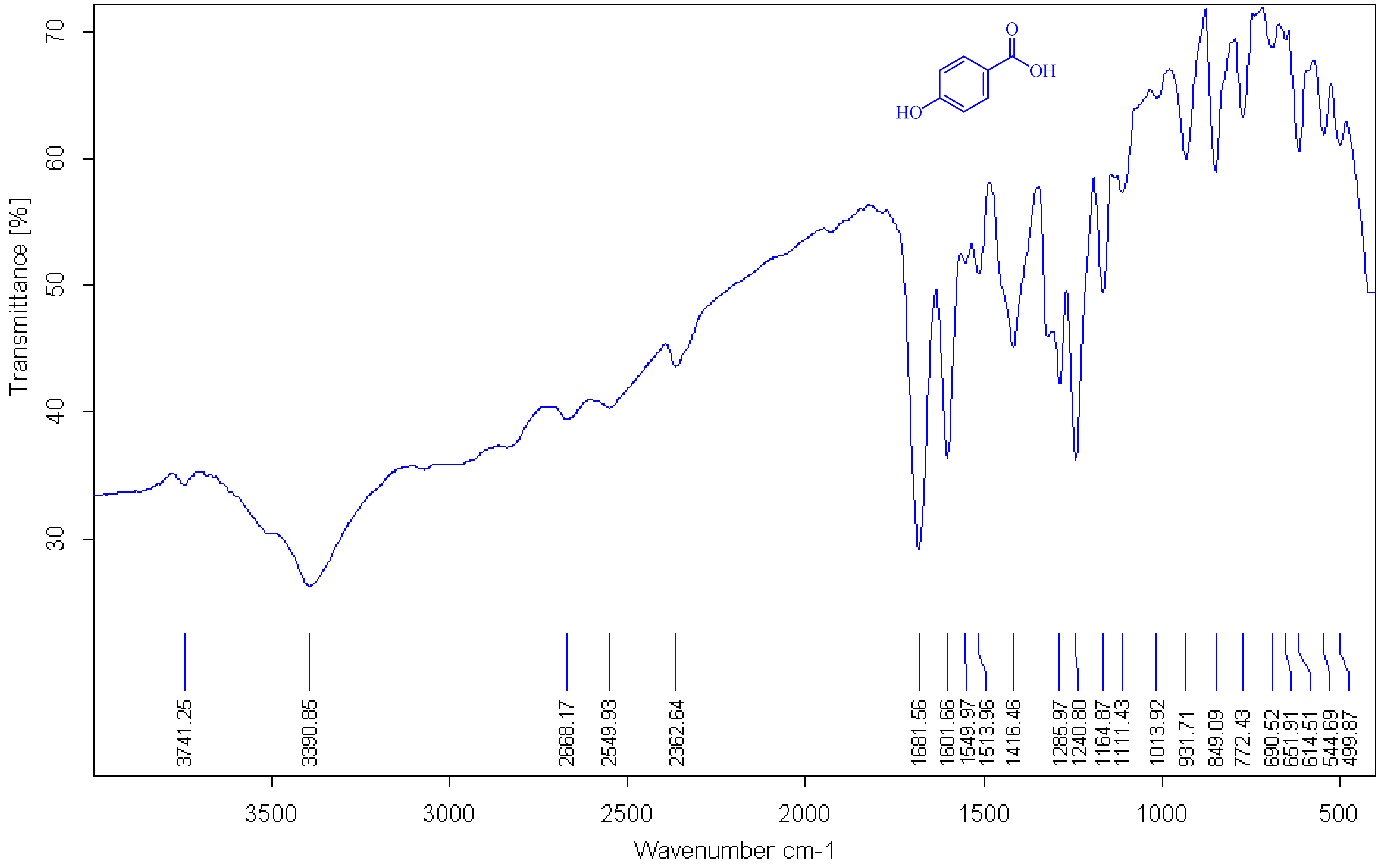

Fig. 12 FT-IR of 4-hydroxybenzoic acid (**2d**)

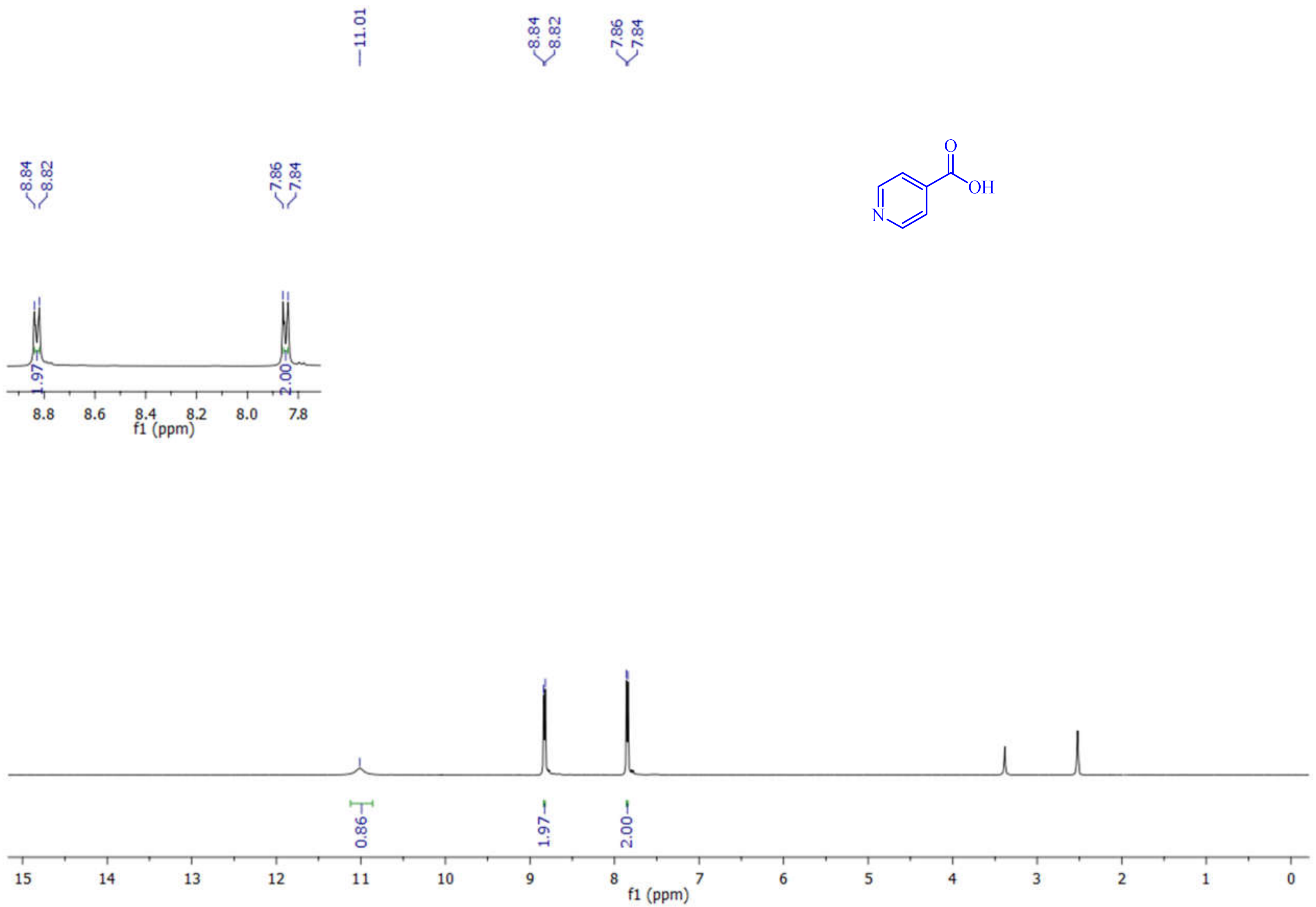

Fig. 13 ^{1}H NMR of isonicotinic acid (**2e**)

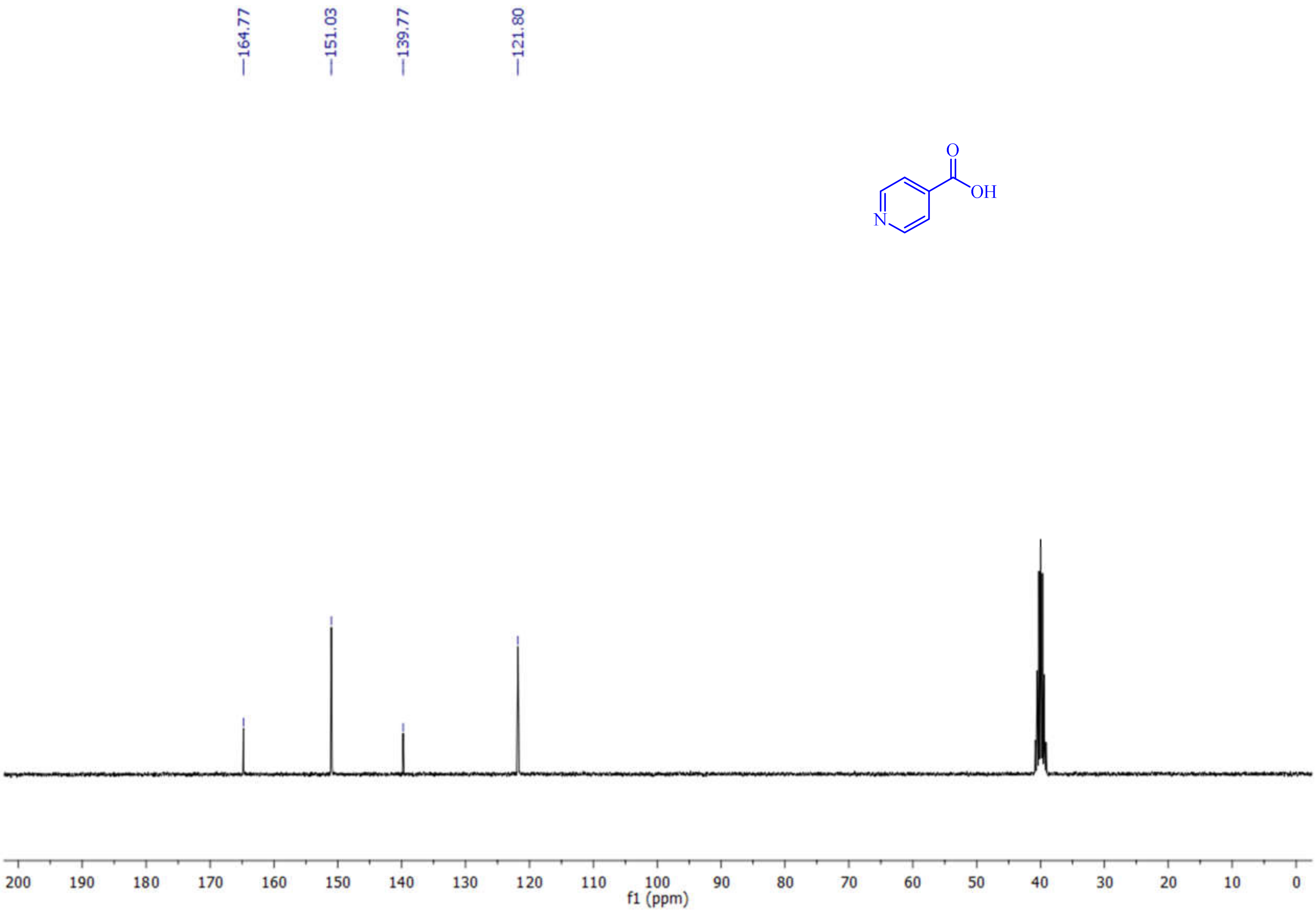

Fig. 14 ^{13}C NMR of isonicotinic acid (**2e**)

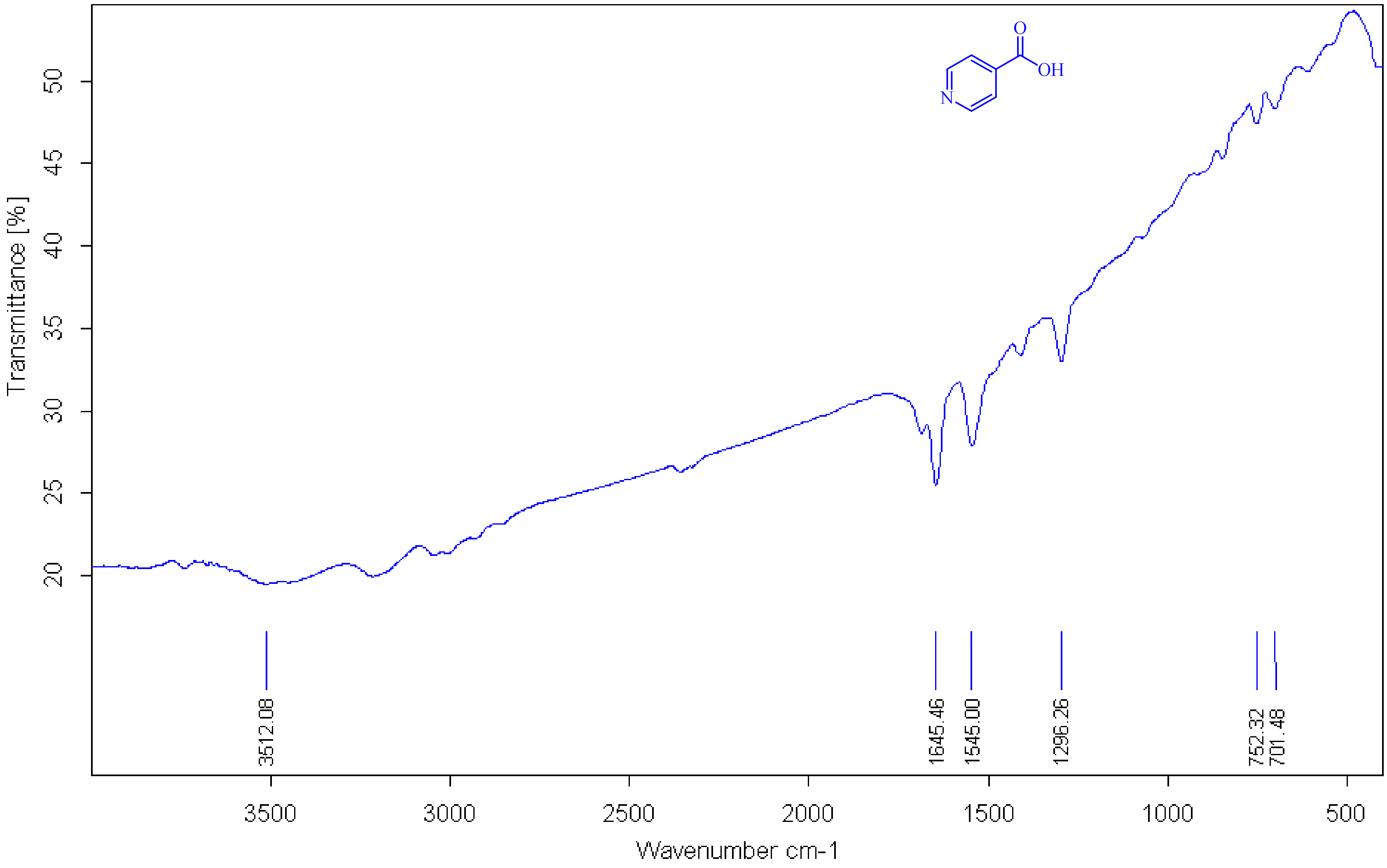

Fig. 15 FT-IR of isonicotinic acid (**2e**)

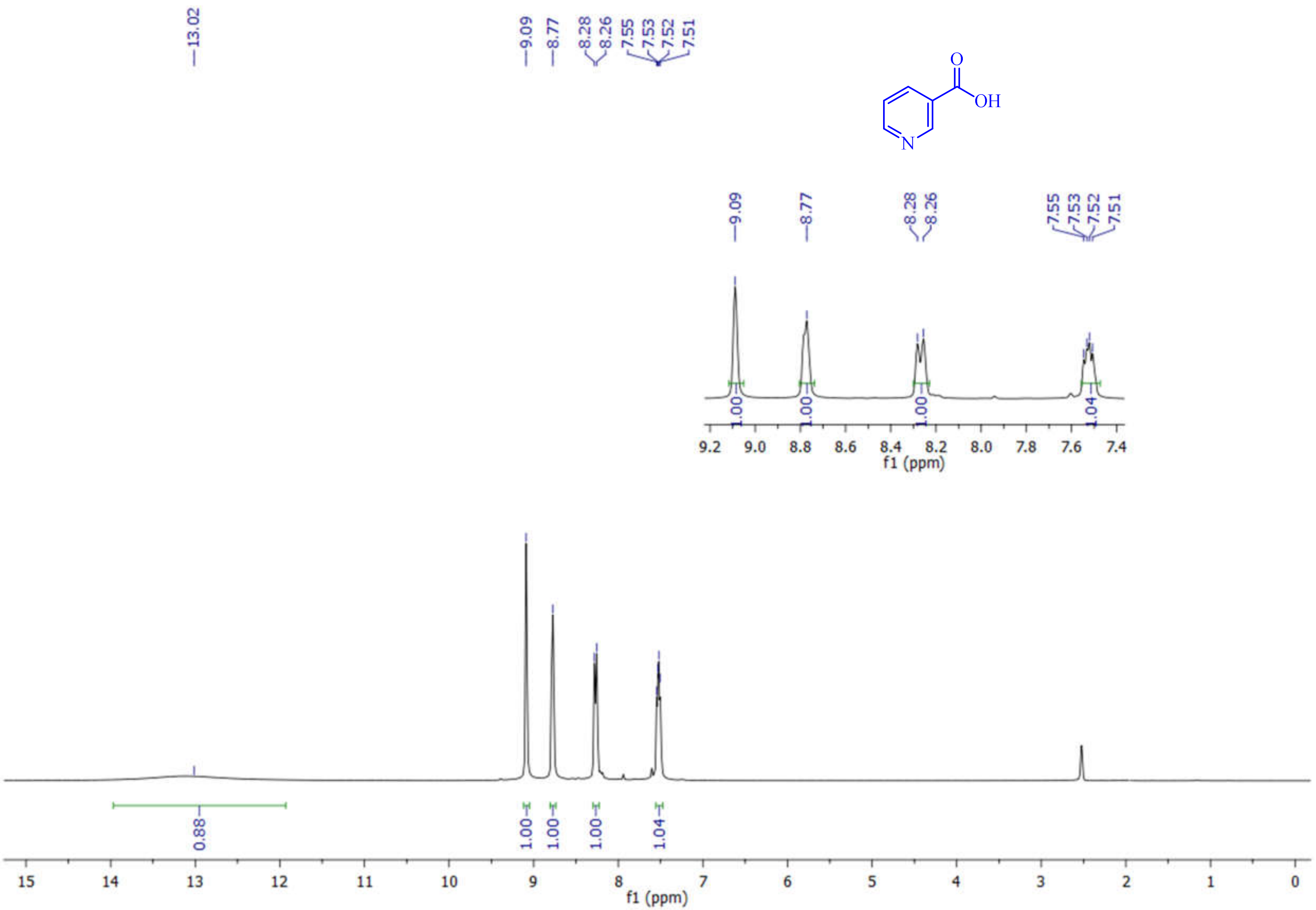

Fig. 16 ^{1}H NMR of nicotinic acid (**2f**)

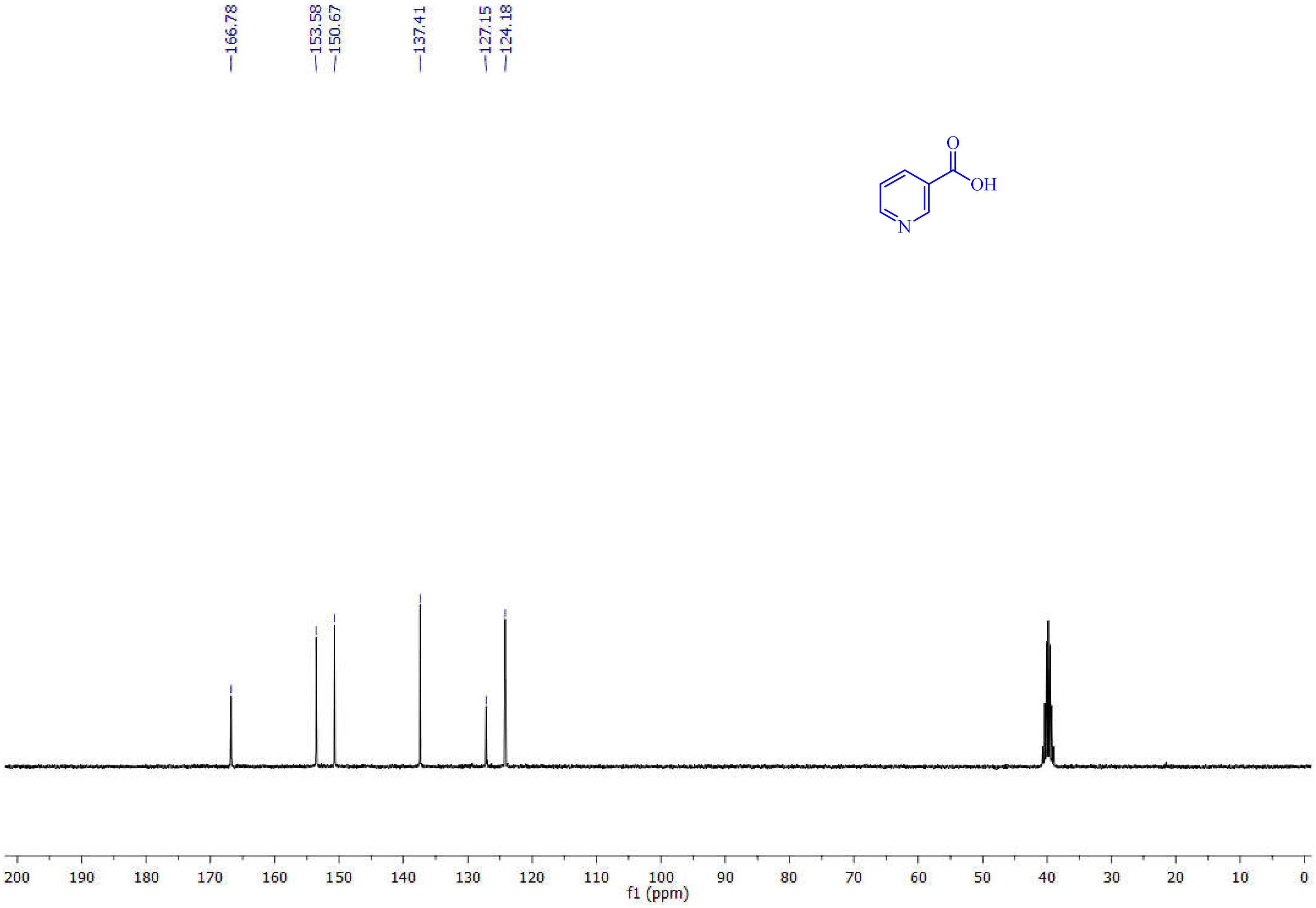

Fig. 17 ^{13}C NMR of nicotinic acid (**2f**)

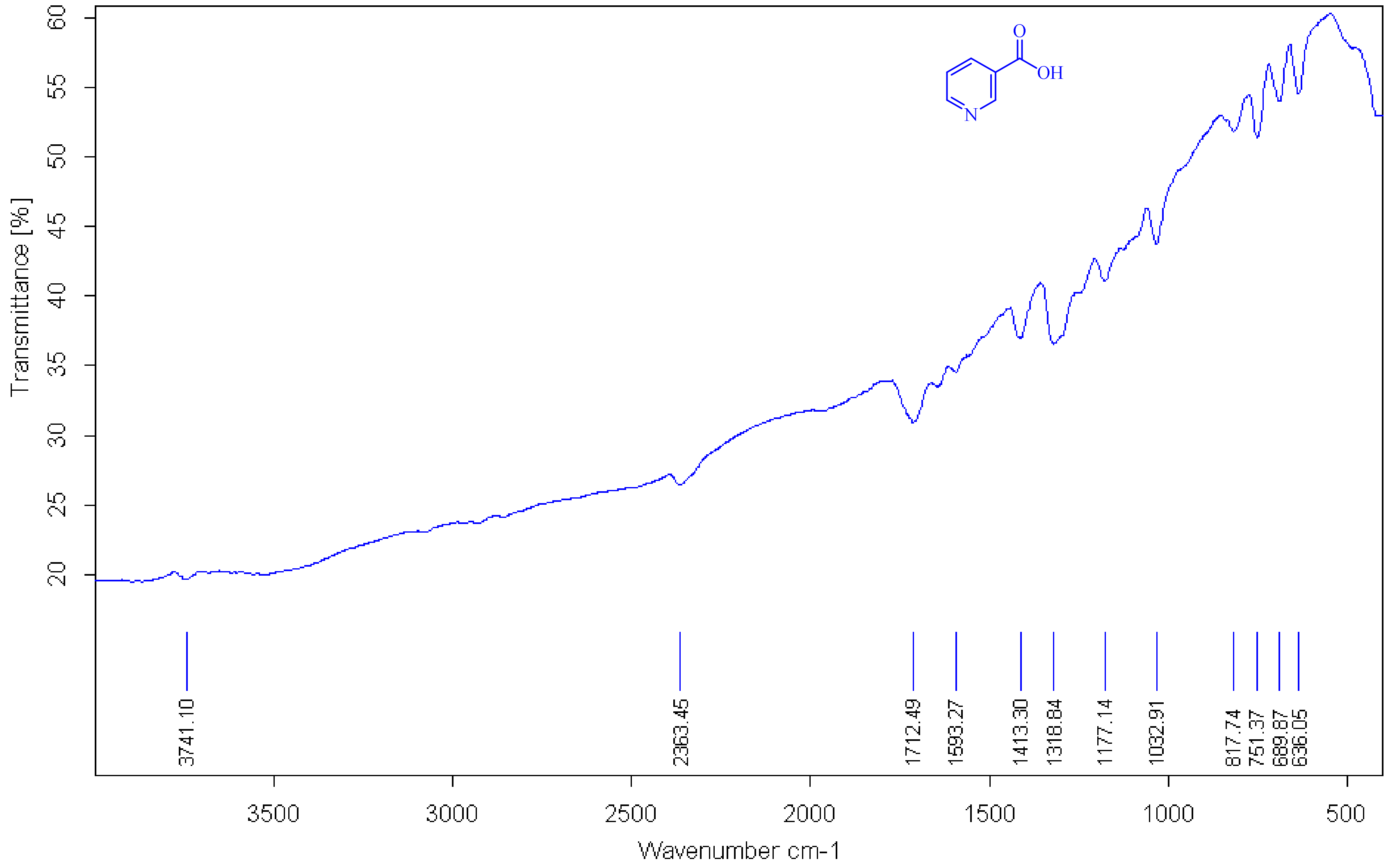

Fig. 18 FT-IR of nicotinic acid (**2f**)

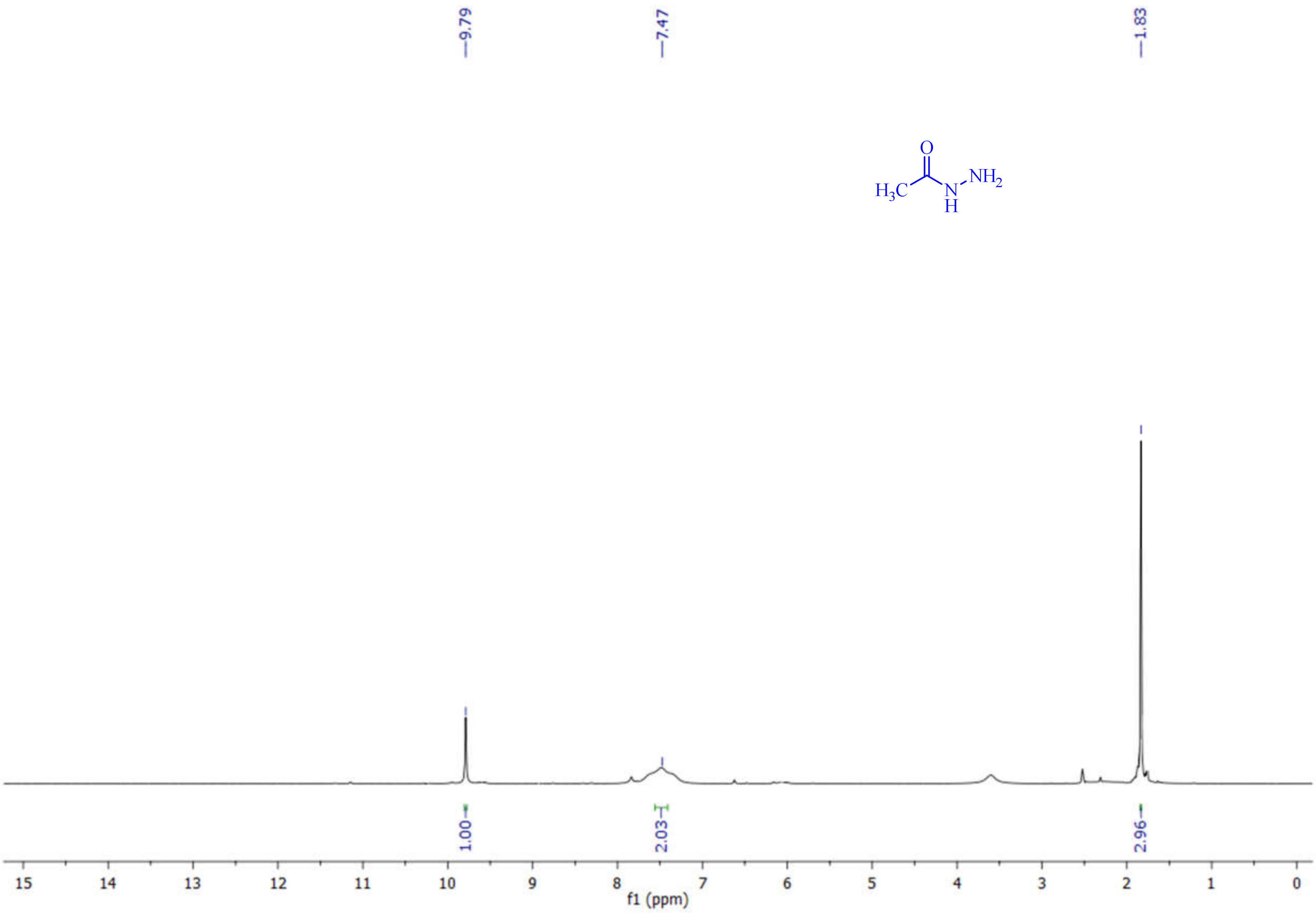

Fig. 19 ^{1}H NMR of acetohydrazide (**2g**)

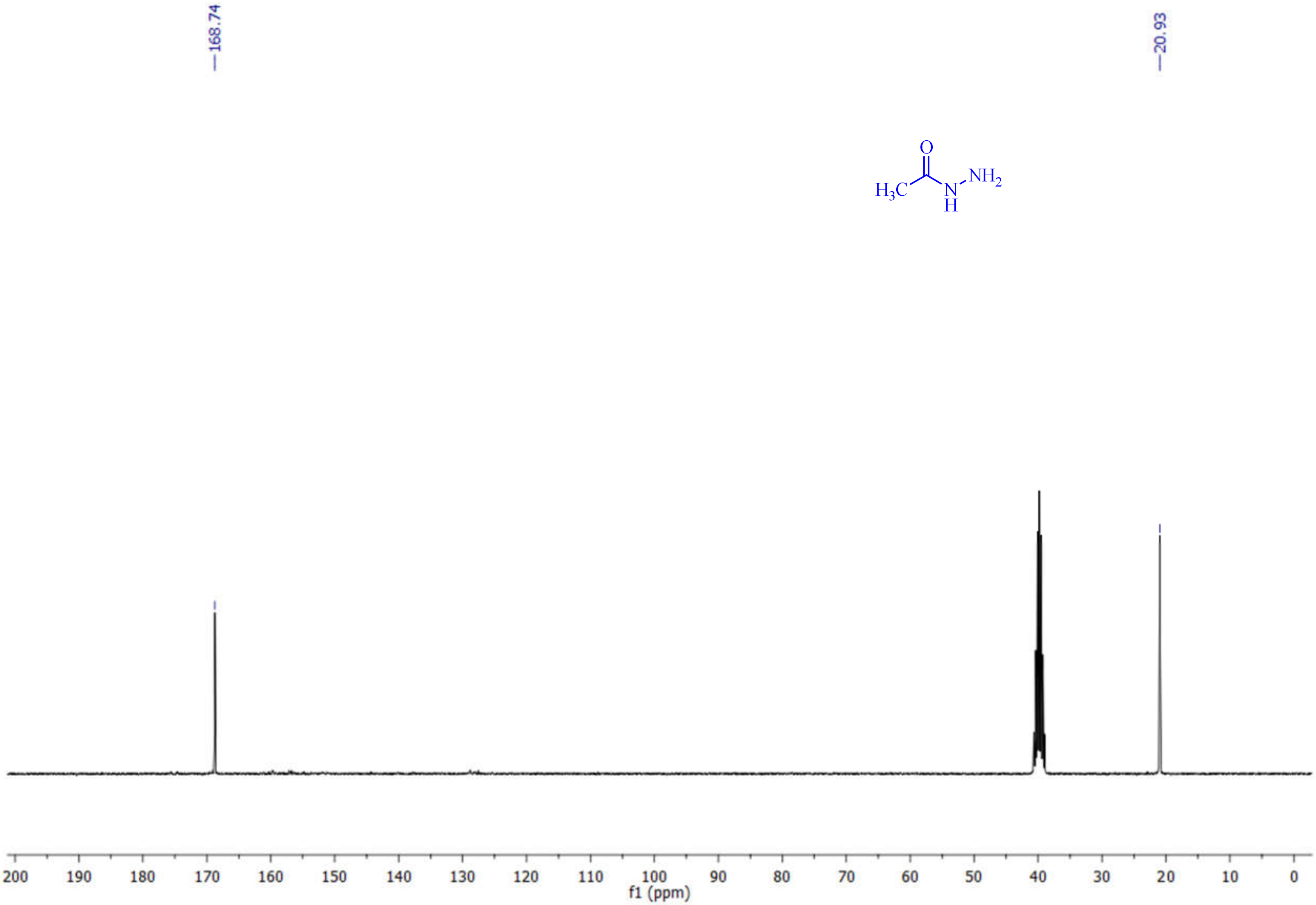

Fig. 20 ^{13}C NMR of acetohydrazide (**2g**)

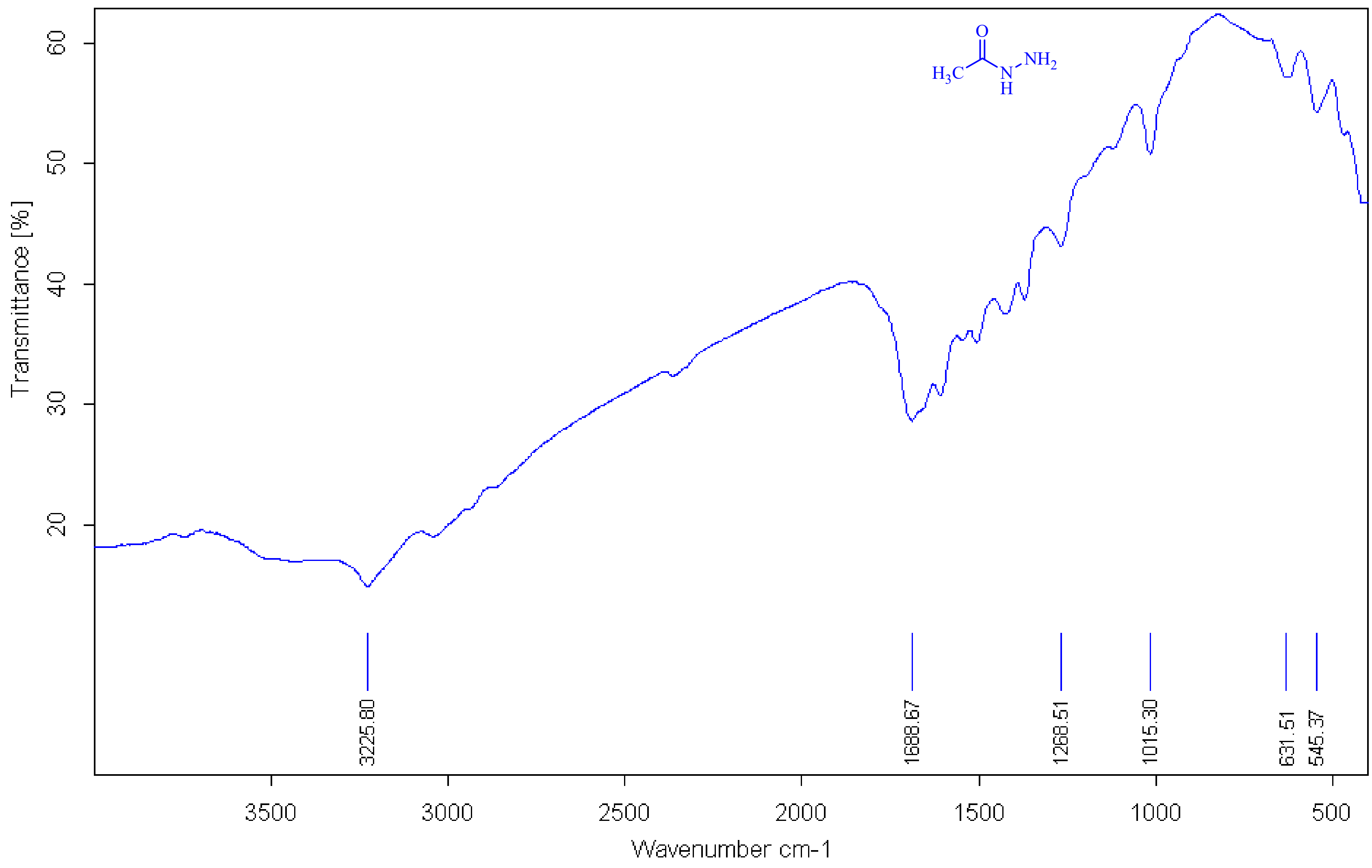

Fig. 21 FT-IR of acetohydrazide (**2g**)

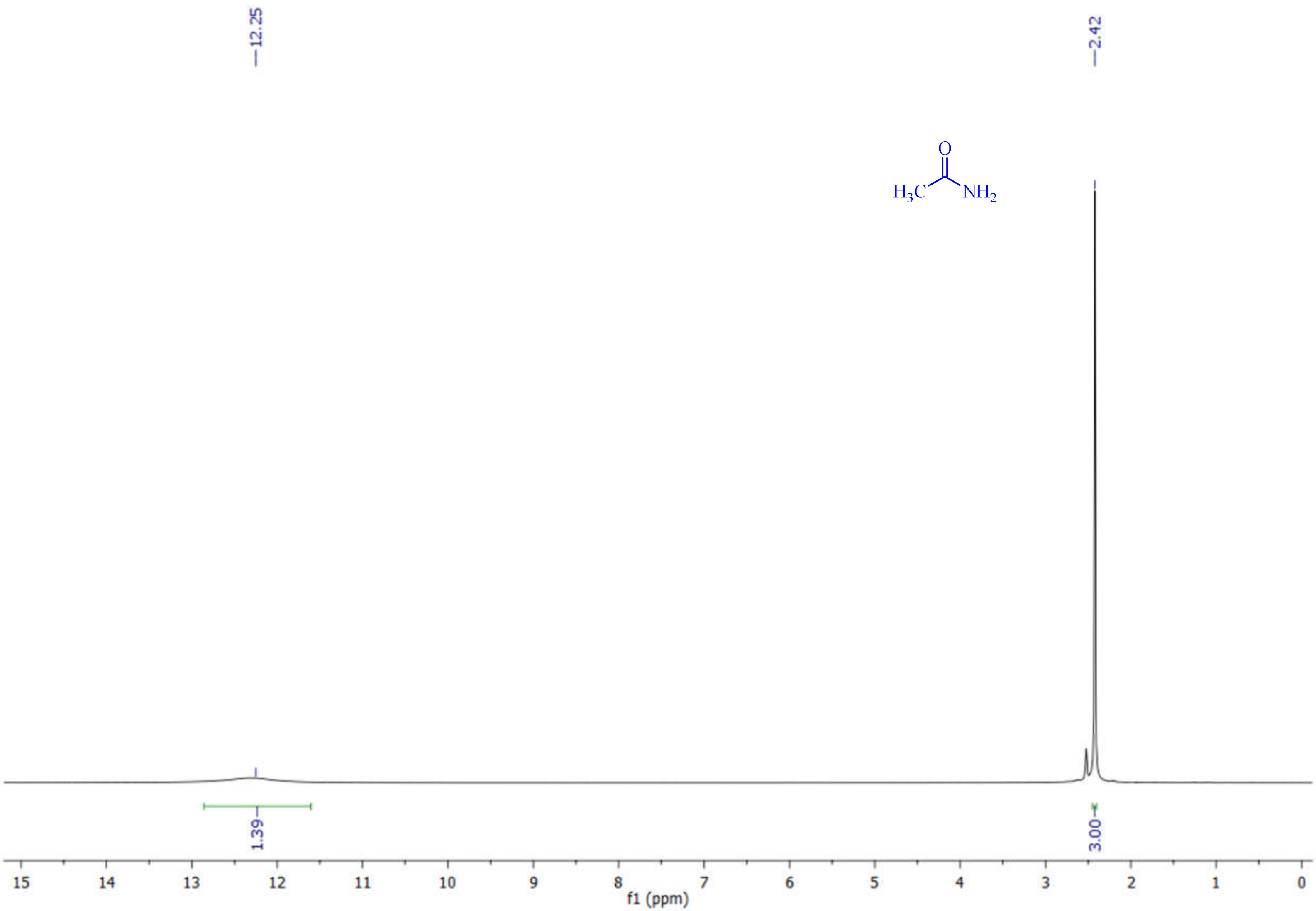

Fig. 22 ^{1}H NMR of acetamide (**2h**)

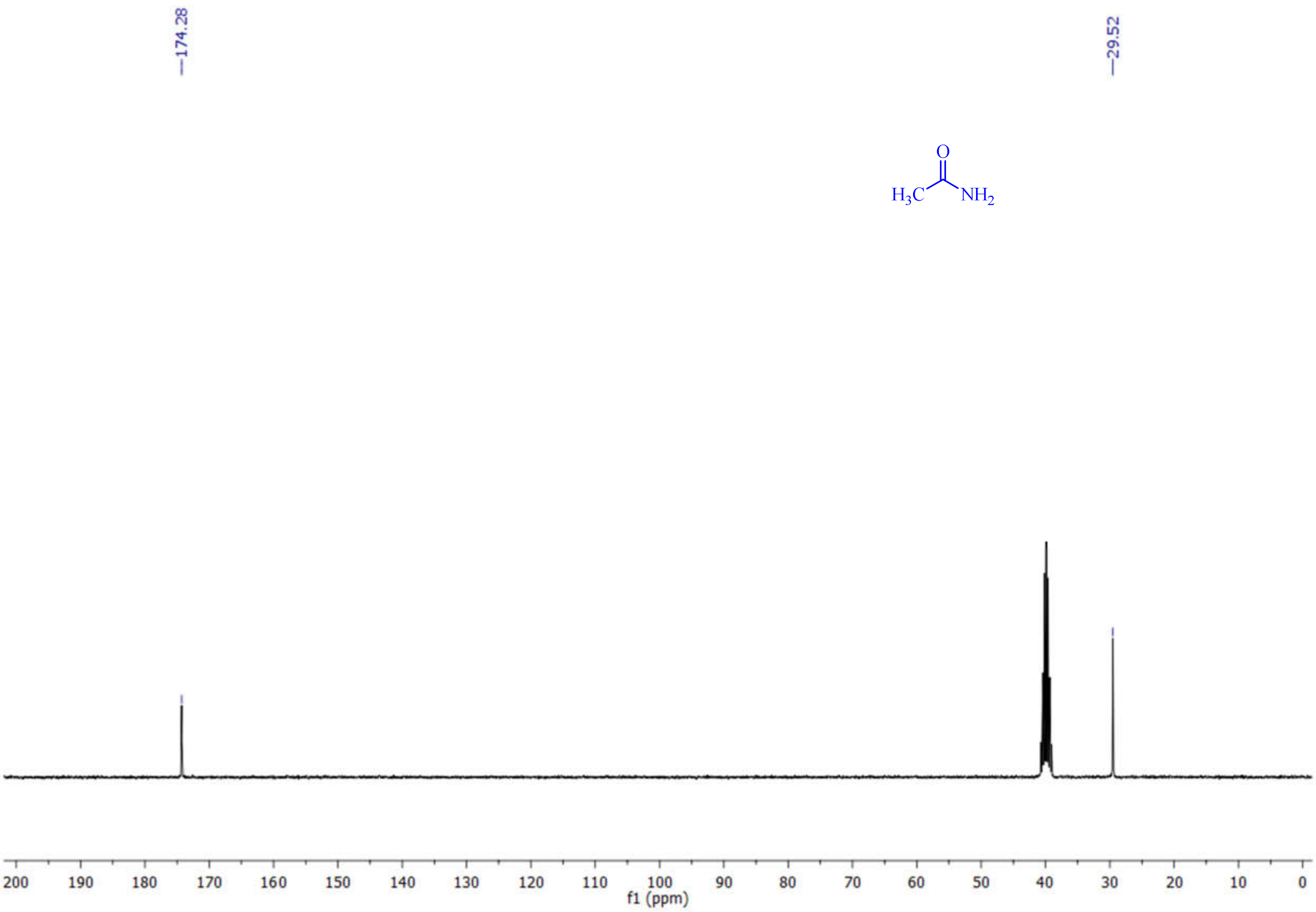

Fig. 23 ^{13}C NMR of acetamide (**2h**)

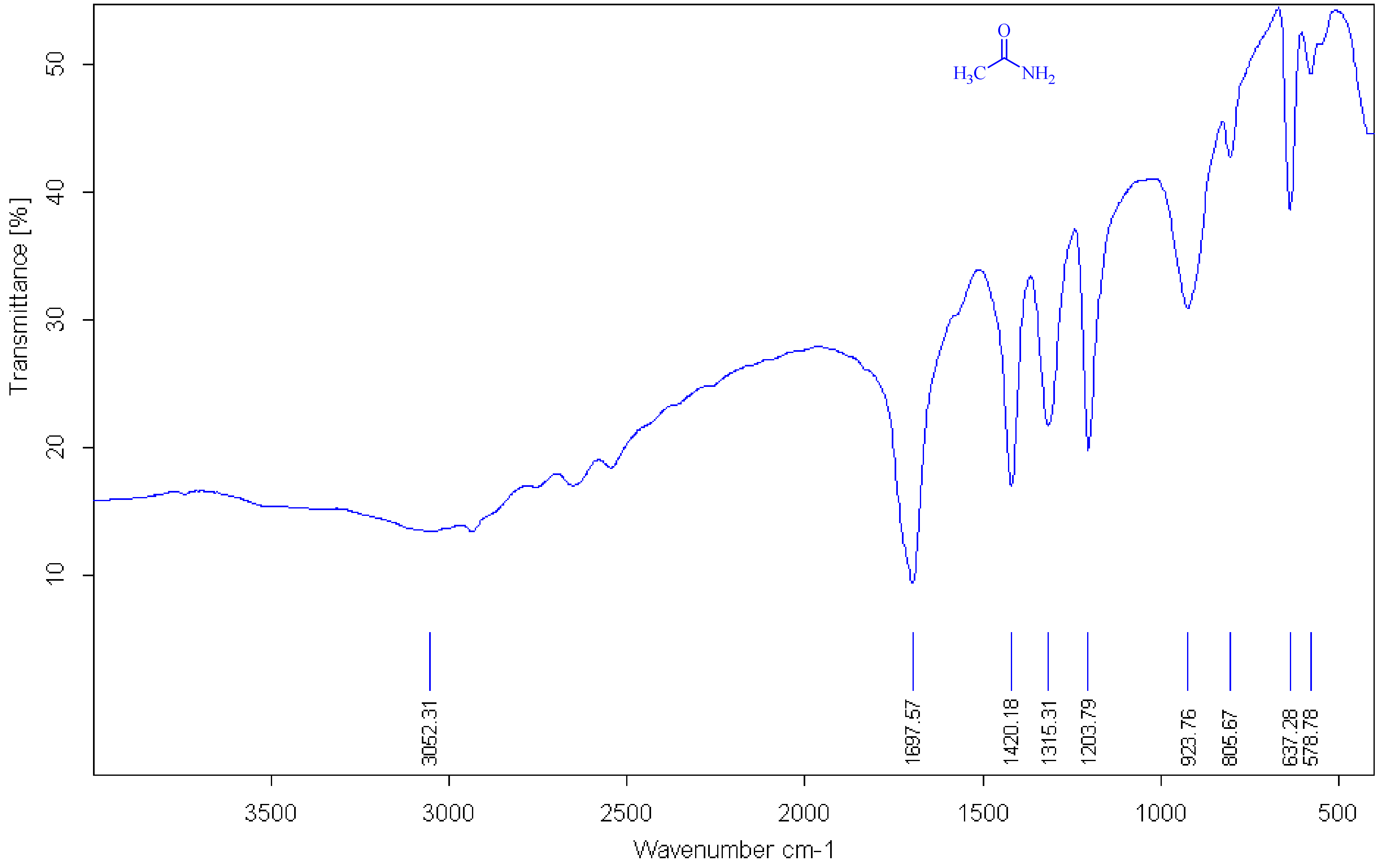

Fig. 24 FT-IR of acetamide (**2h**)

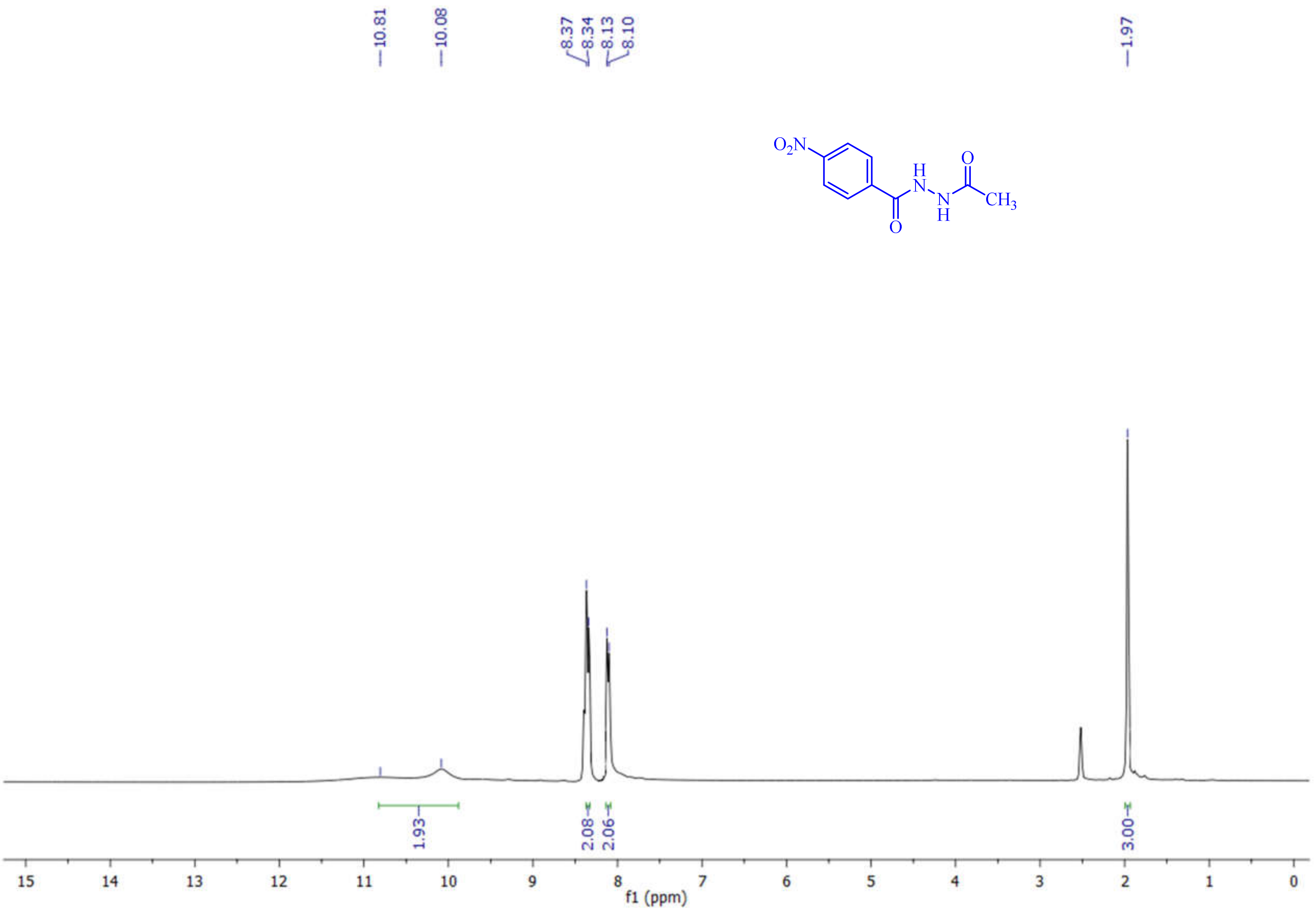

Fig. 25 ^{1}H NMR of *N*'-acetyl-4-nitrobenzohydrazide (**2i**)

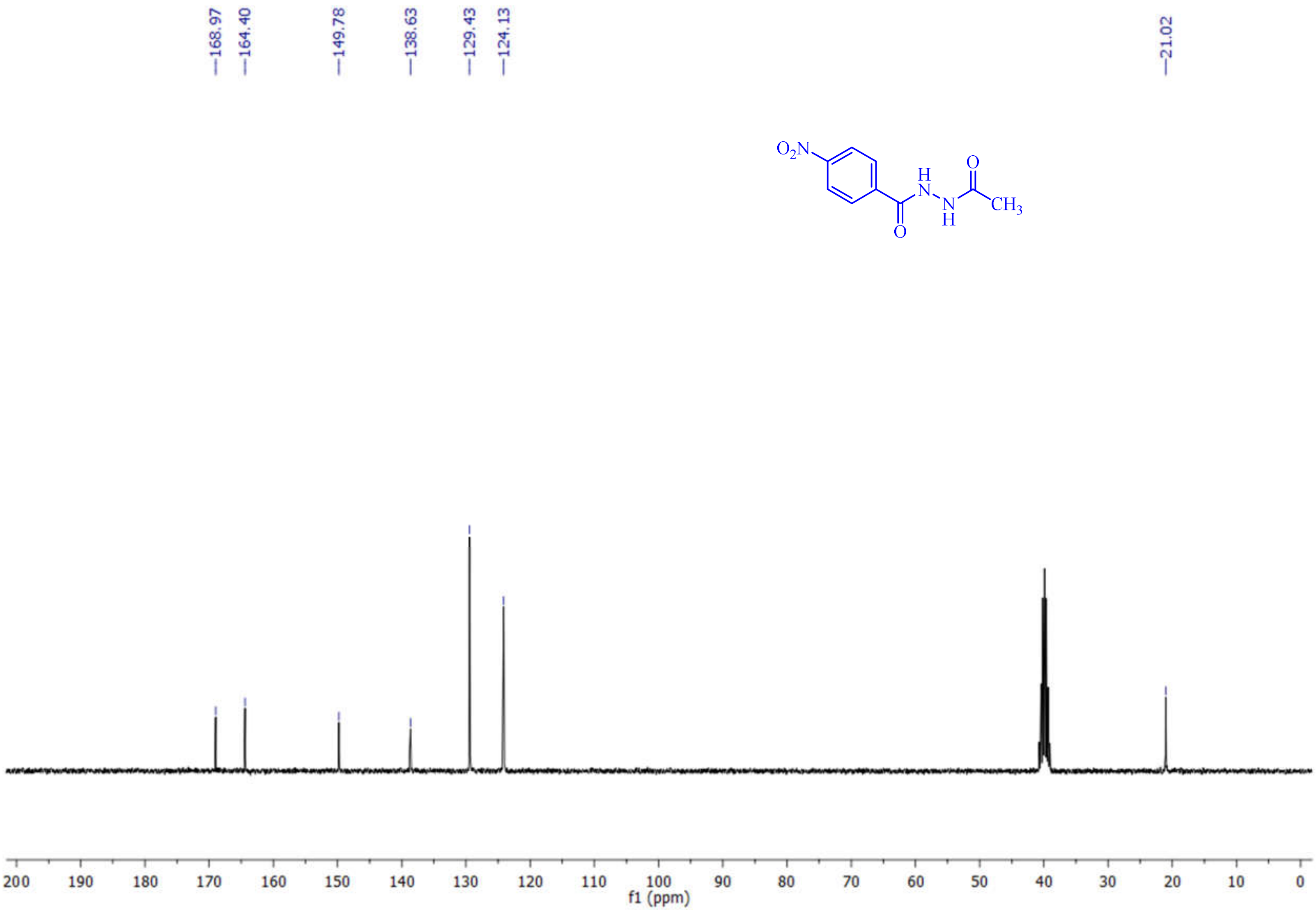

Fig. 26 ^{13}C NMR of *N*'-acetyl-4-nitrobenzohydrazide (**2i**)

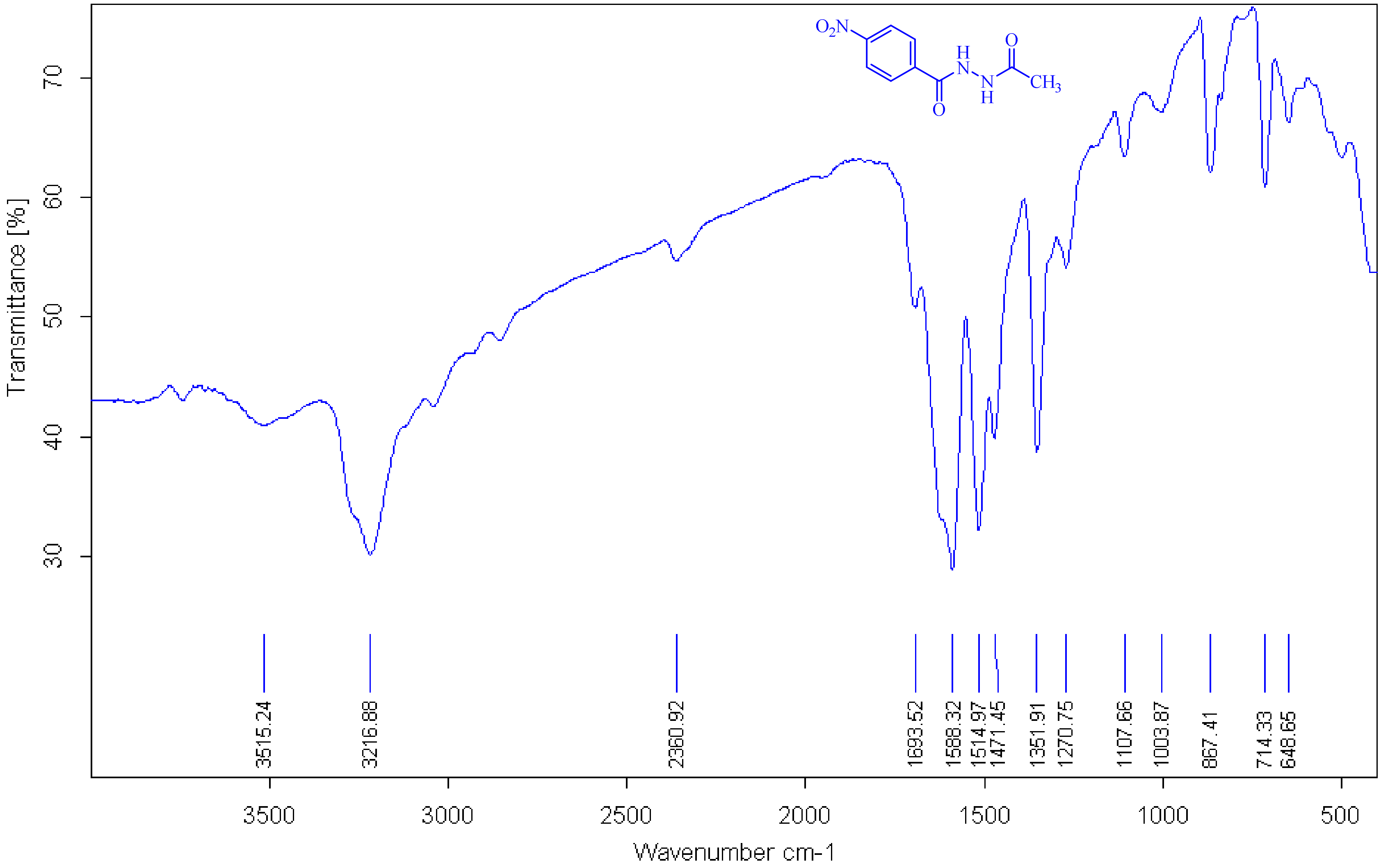

Fig. 27 FT-IR of *N*'-acetyl-4-nitrobenzohydrazide (**2i**)

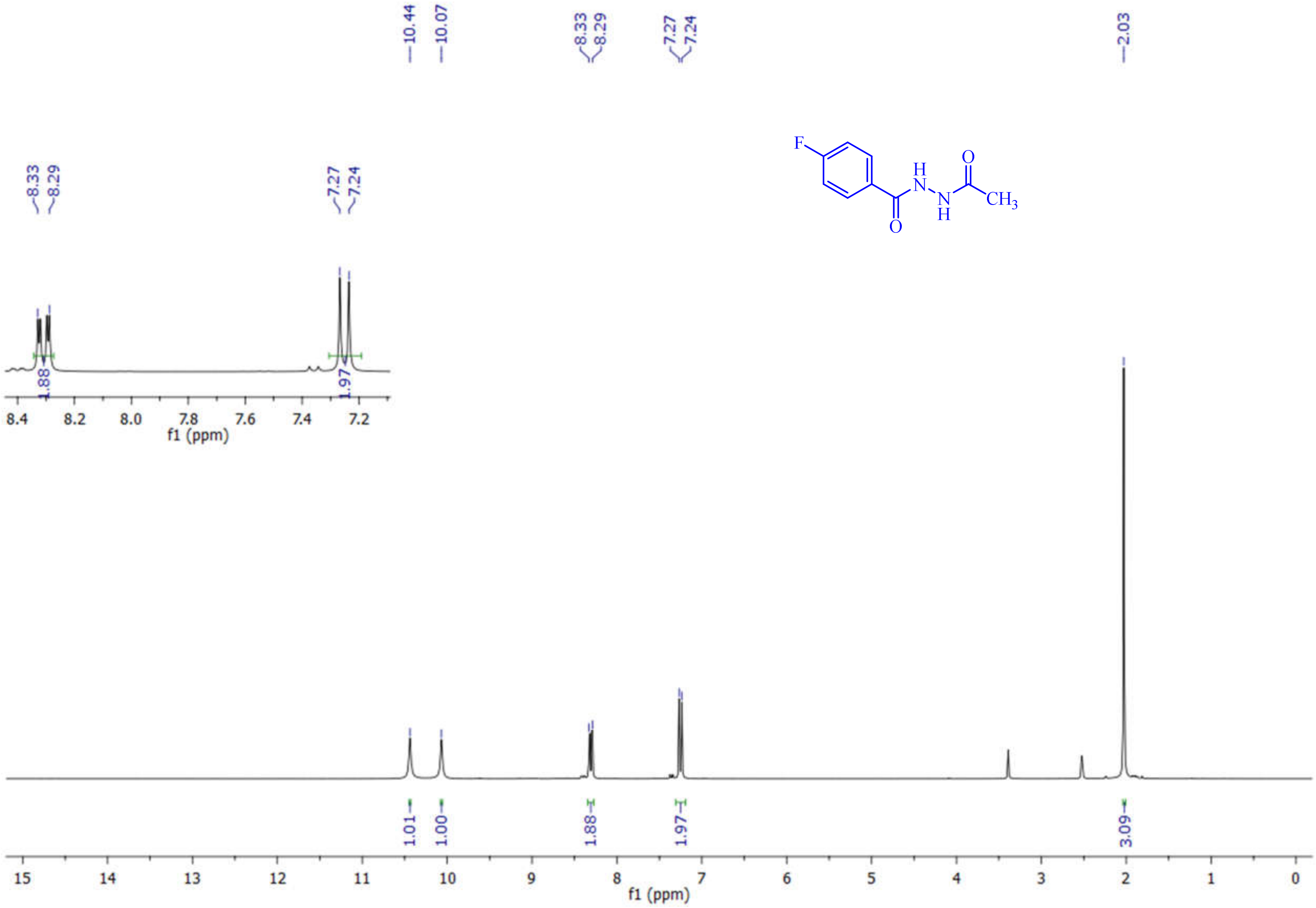

Fig. 28 ^{1}H NMR of *N*'-acetyl-4-fluorobenzohydrazide (**2j**)

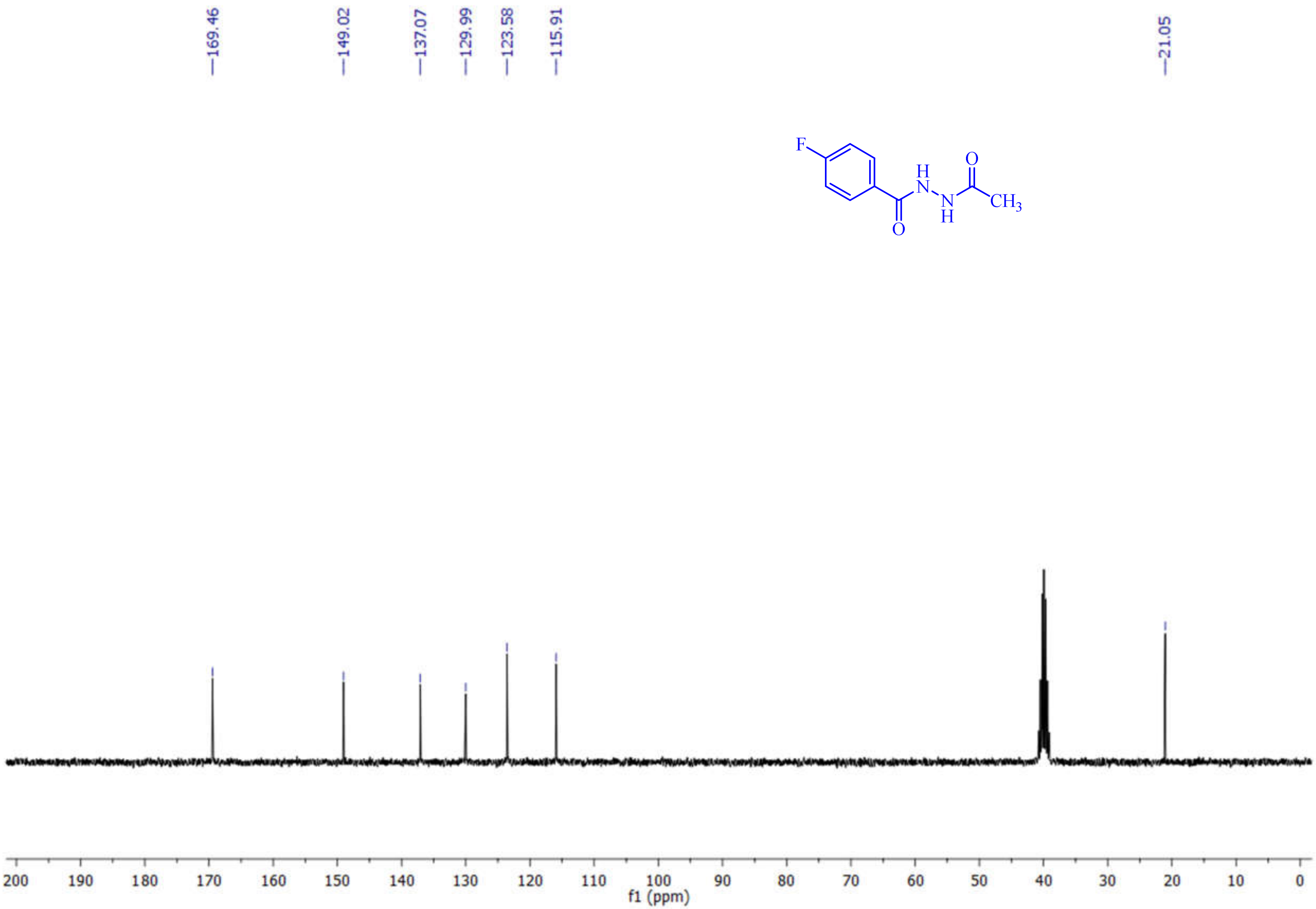

Fig. 29 ^{13}C NMR of *N'*-acetyl-4-fluorobenzohydrazide (**2j**)

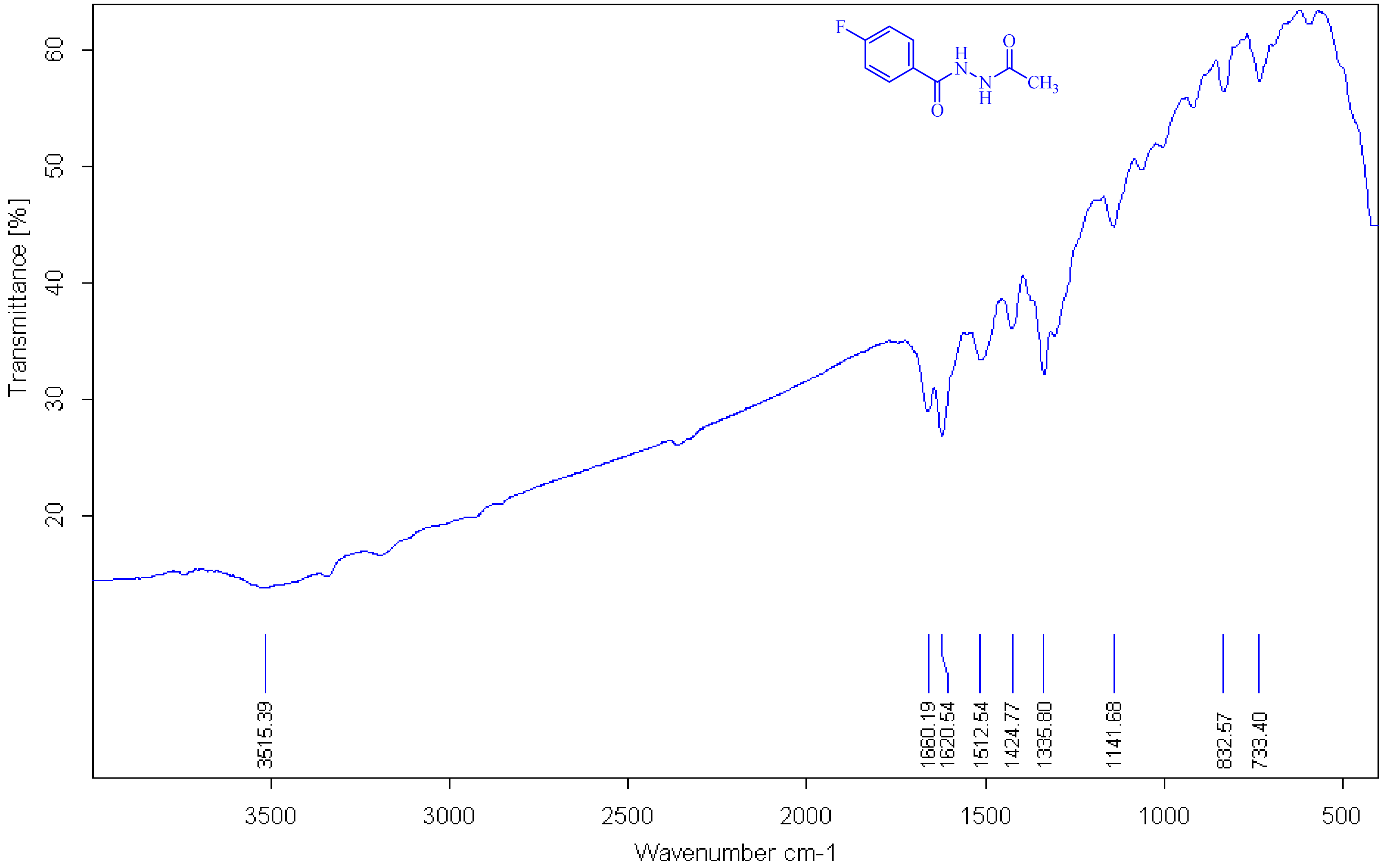

Fig. 30 FT-IR of *N'*-acetyl-4-fluorobenzohydrazide (**2j**)

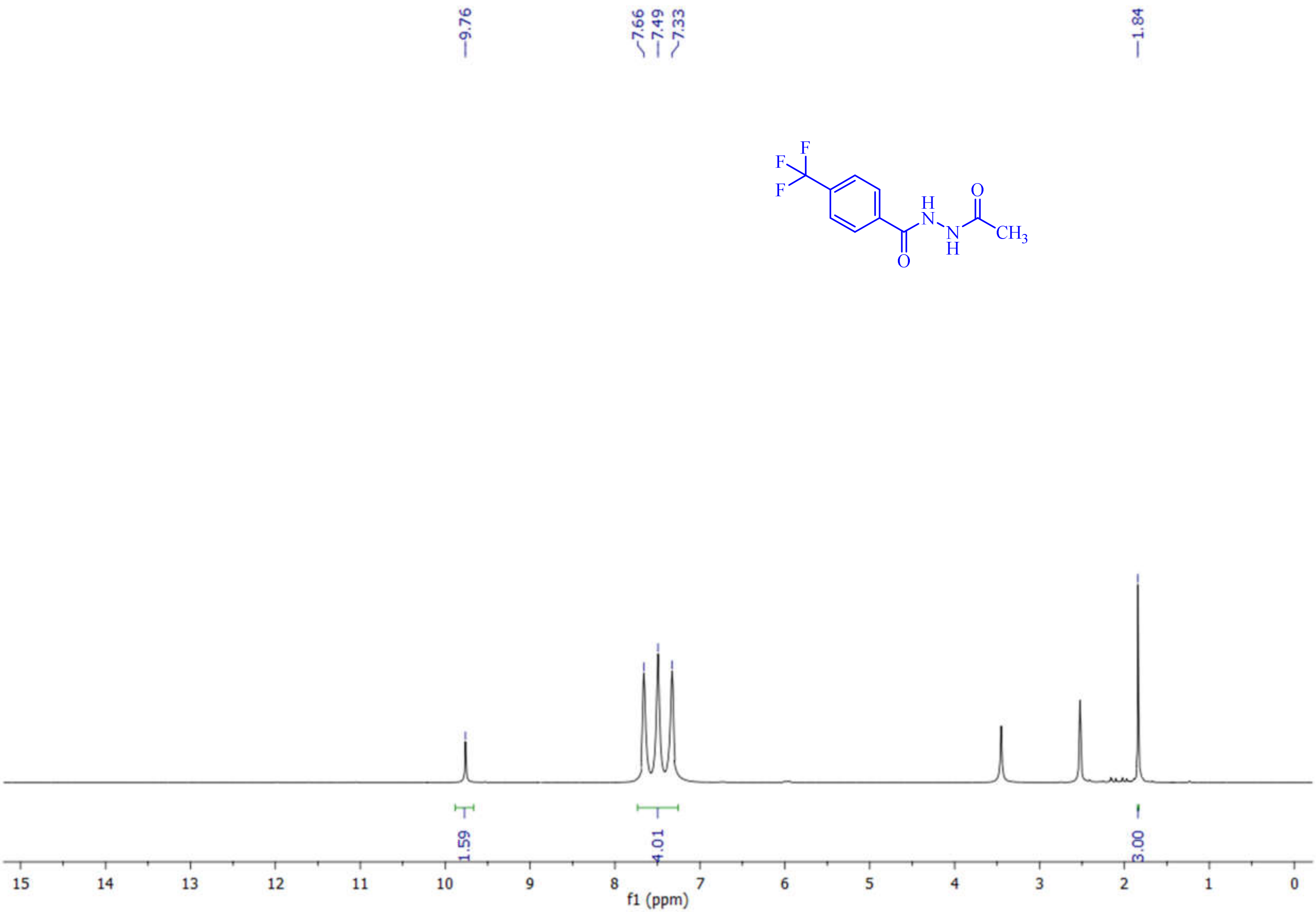

Fig. 31 ^{1}H NMR of *N'*-acetyl-4-(trifluoromethyl)benzohydrazide (**2k**)

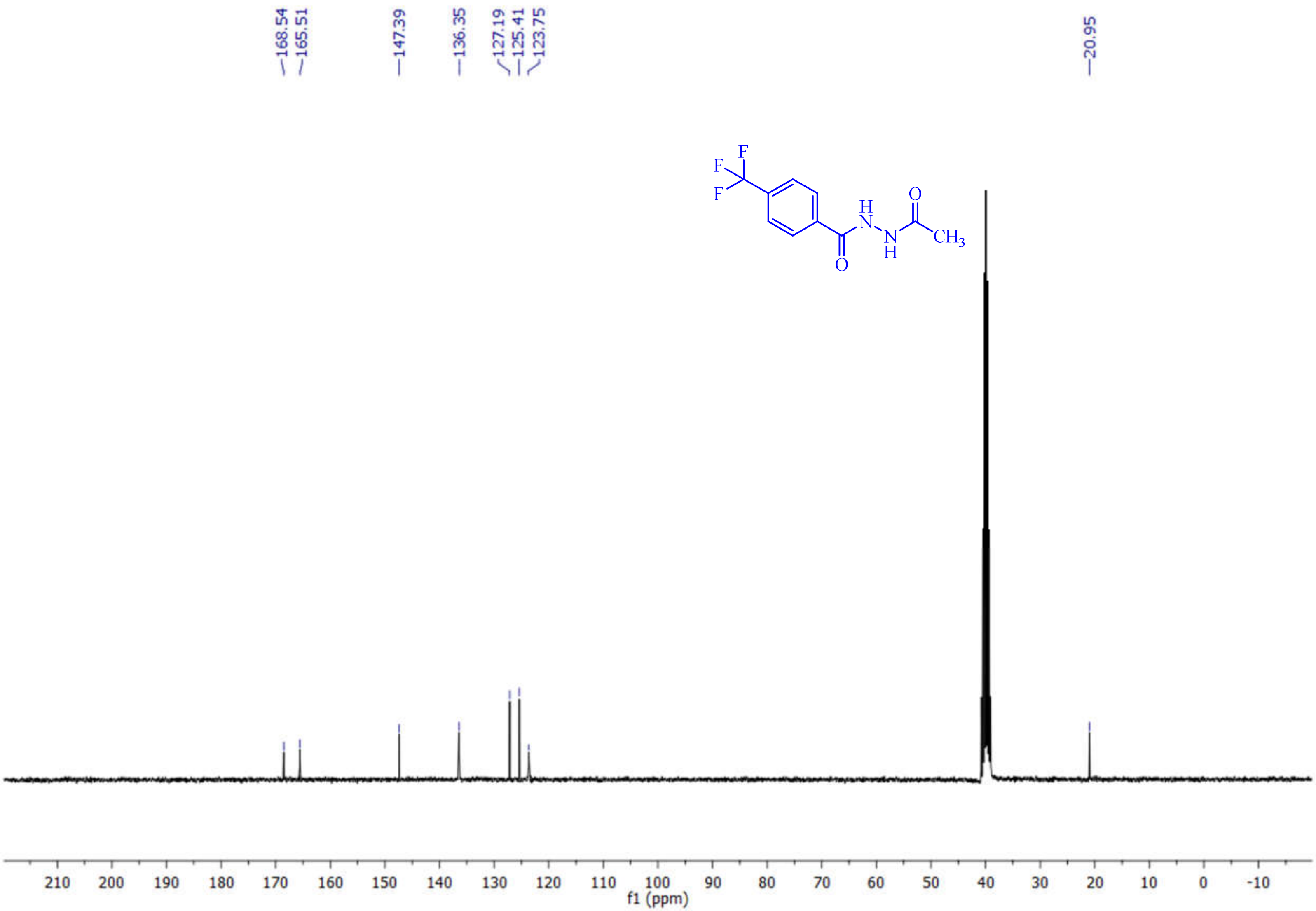

Fig. 32 ^{13}C NMR of *N*'-acetyl-4-(trifluoromethyl)benzohydrazide (**2k**)

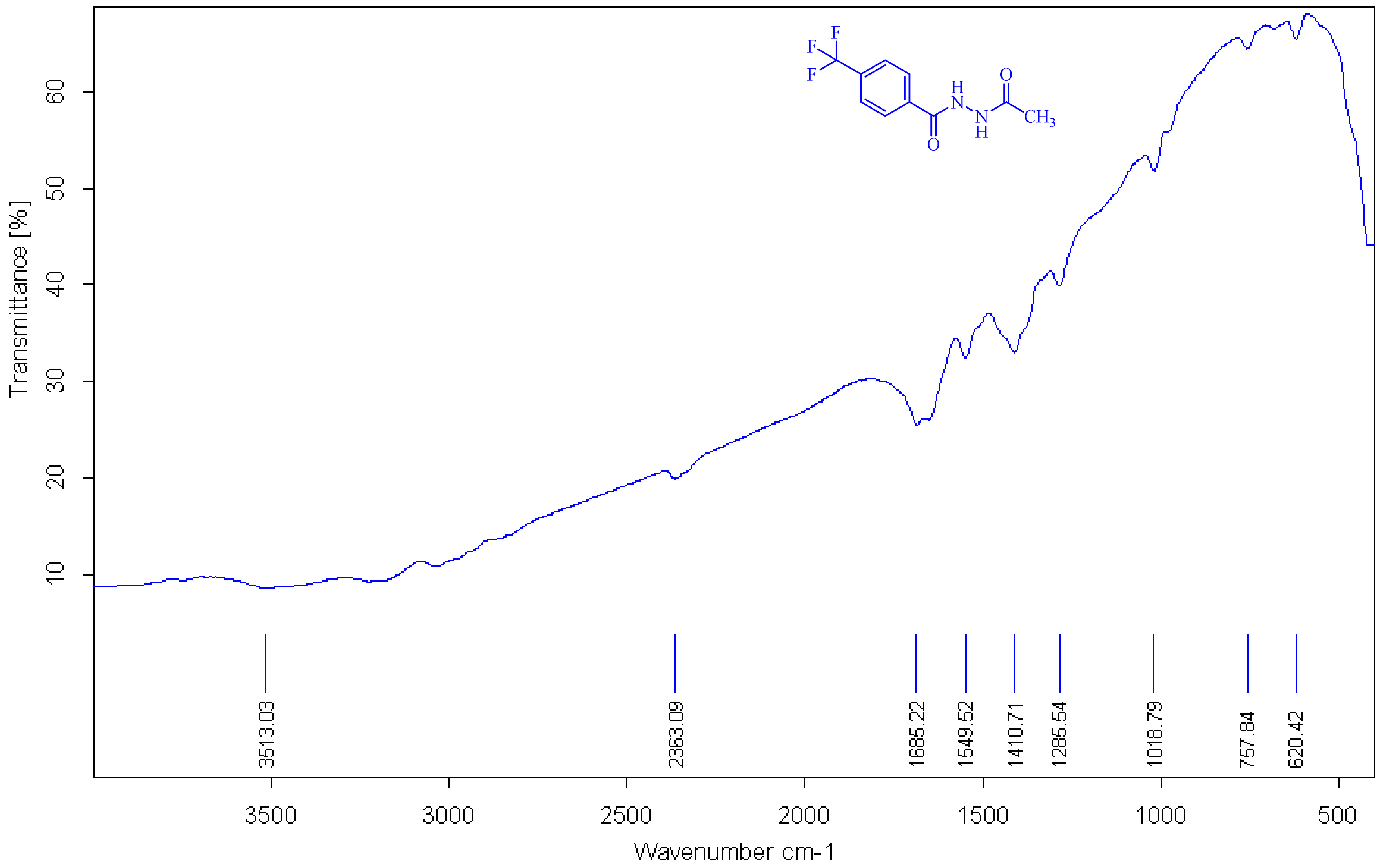

Fig. 33 FT-IR of *N'*-acetyl-4-(trifluoromethyl)benzohydrazide (**2k**)

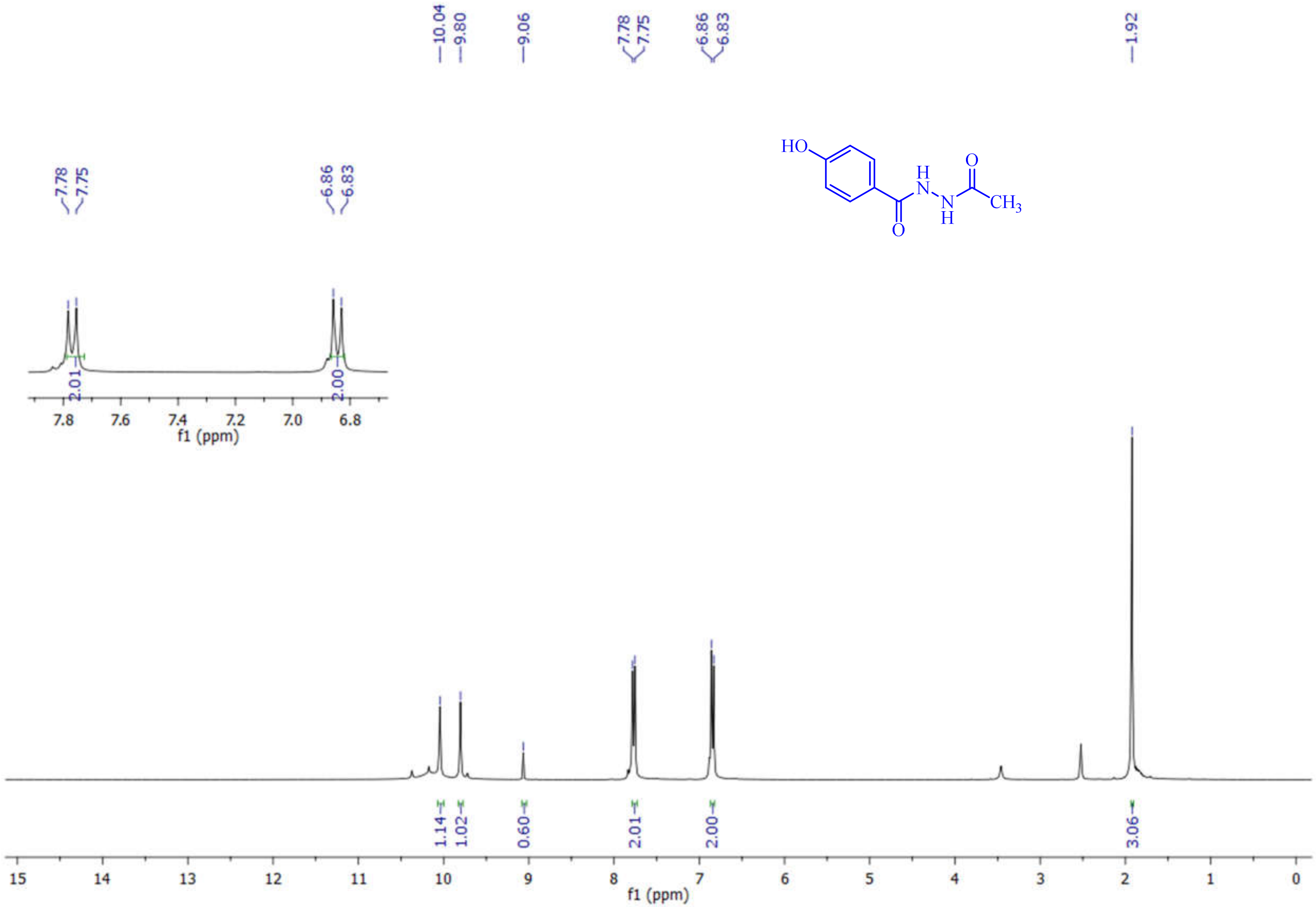

Fig. 34 ^{1}H NMR of *N'*-acetyl-4-hydroxybenzohydrazide (**2l**)

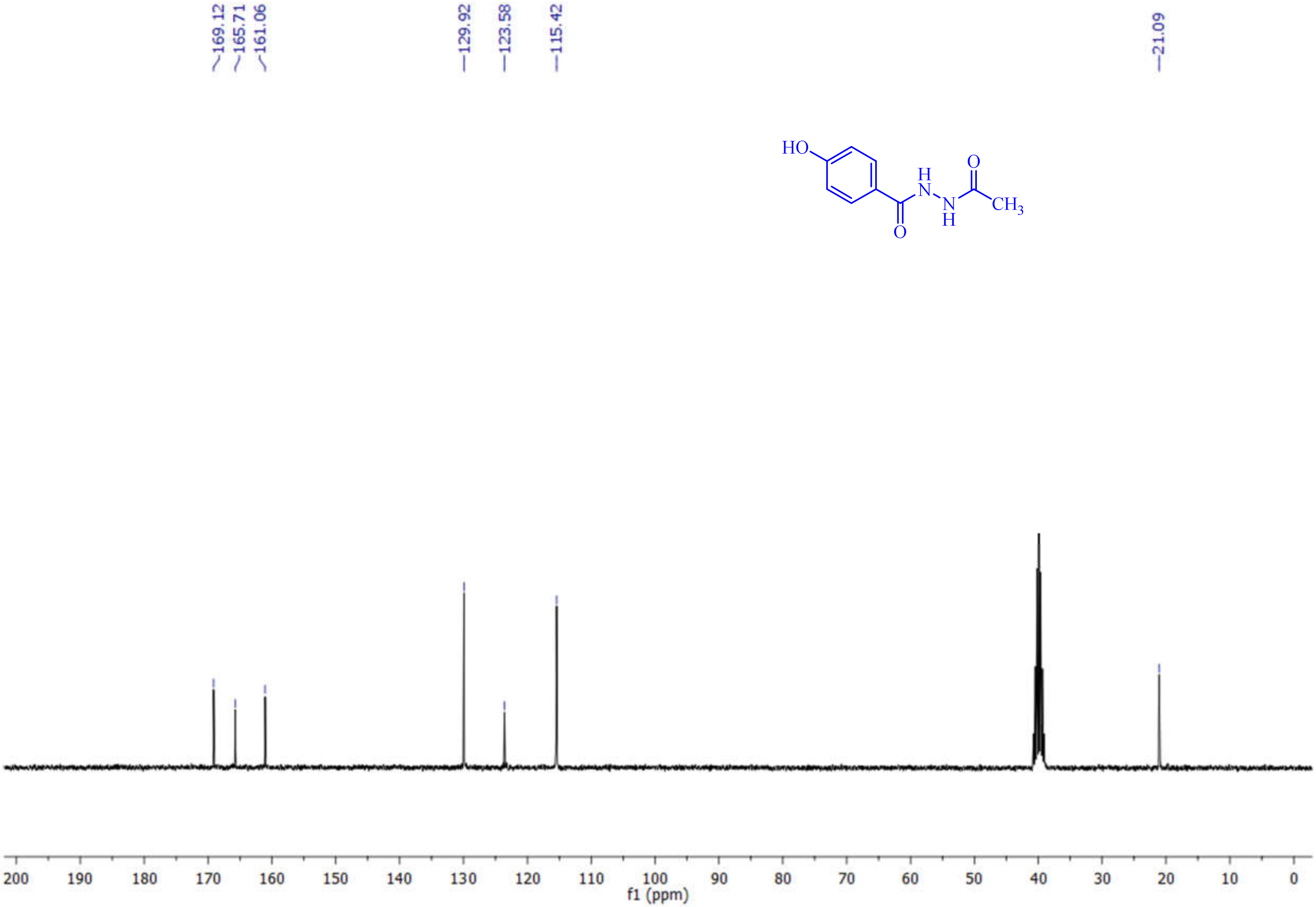

Fig. 35 ^{13}C NMR of *N'*-acetyl-4-hydroxybenzohydrazide (**2l**)

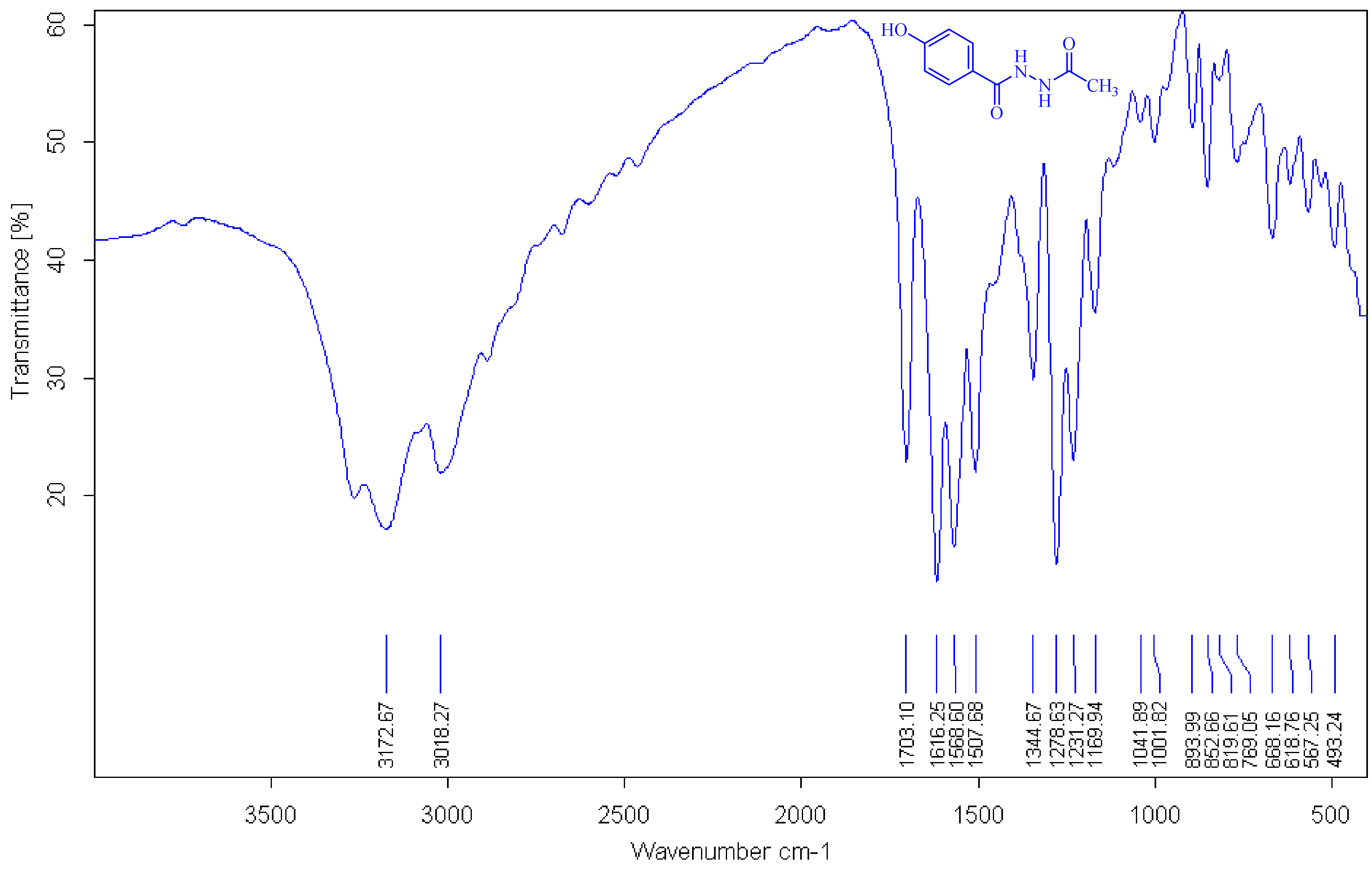

Fig. 36 FT-IR of *N'*-acetyl-4-hydroxybenzohydrazide (**2l**)

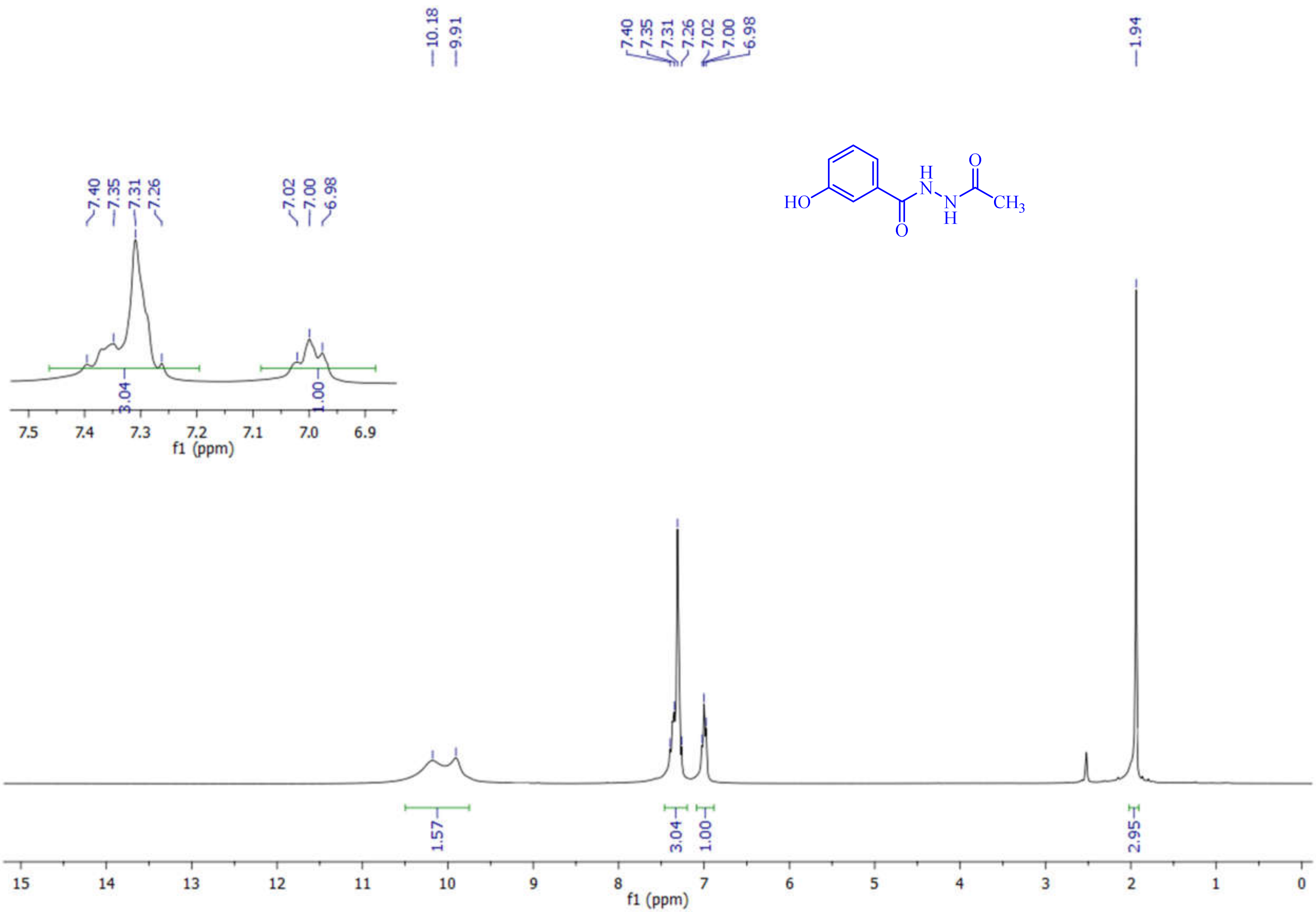

Fig. 37 ^{1}H NMR of *N'*-acetyl-3-hydroxybenzohydrazide (**2m**)

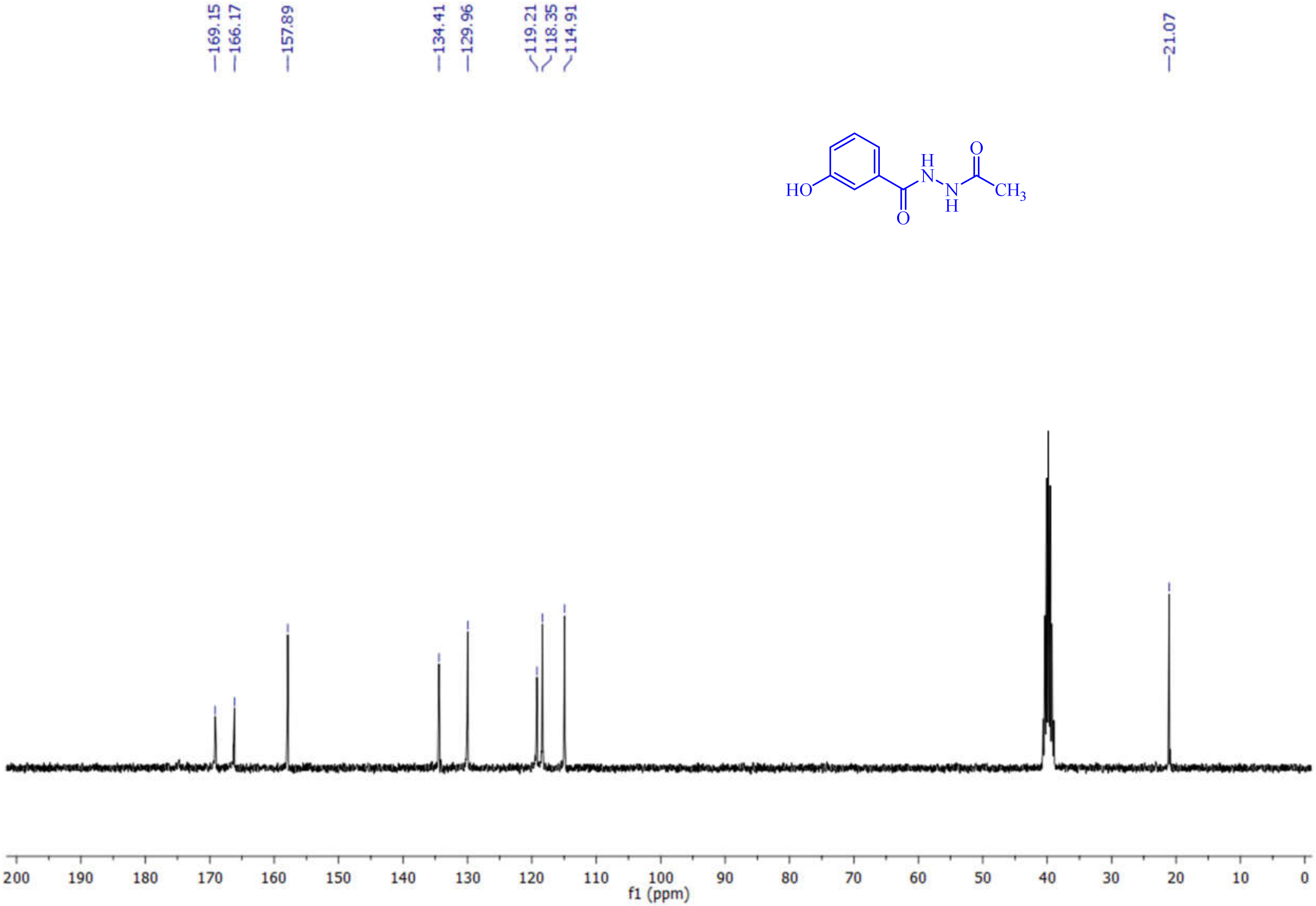

Fig. 38 ^{13}C NMR of *N*'-acetyl-3-hydroxybenzohydrazide (**2m**)

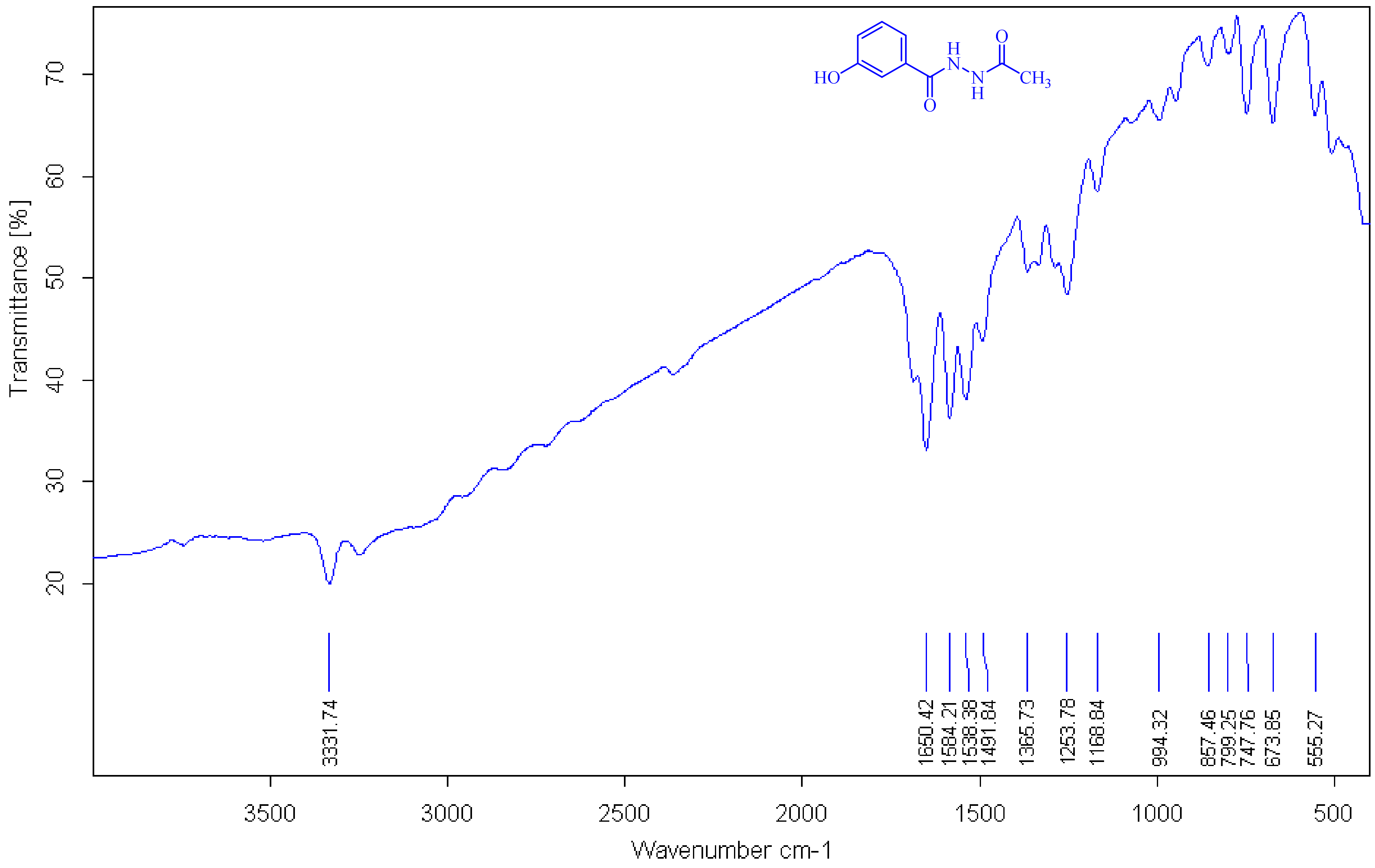

Fig. 39 FT-IR of *N'*-acetyl-3-hydroxybenzohydrazide (**2m**)

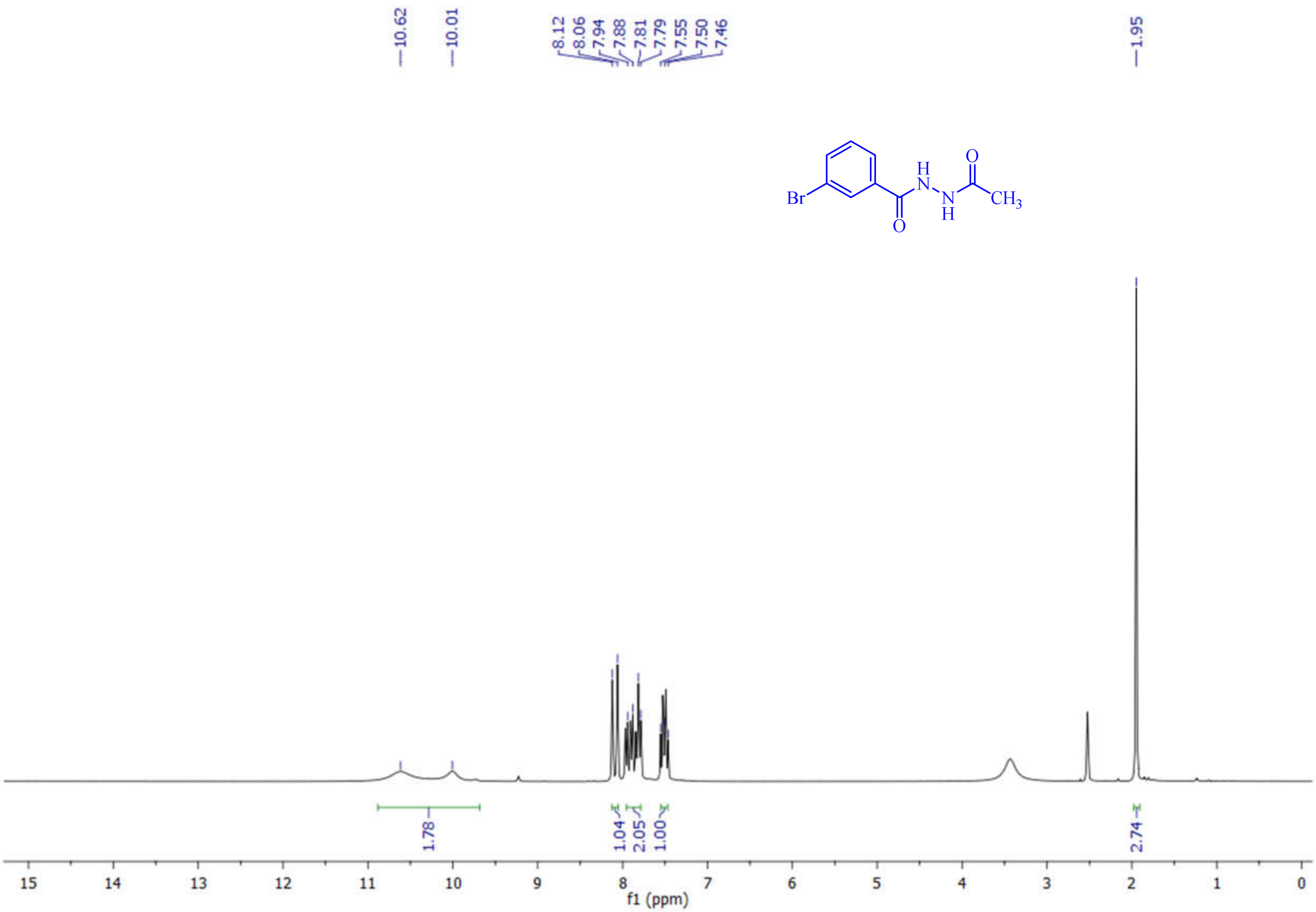

Fig. 40 ^{1}H NMR of *N'*-acetyl-3-bromobenzohydrazide (**2n**)

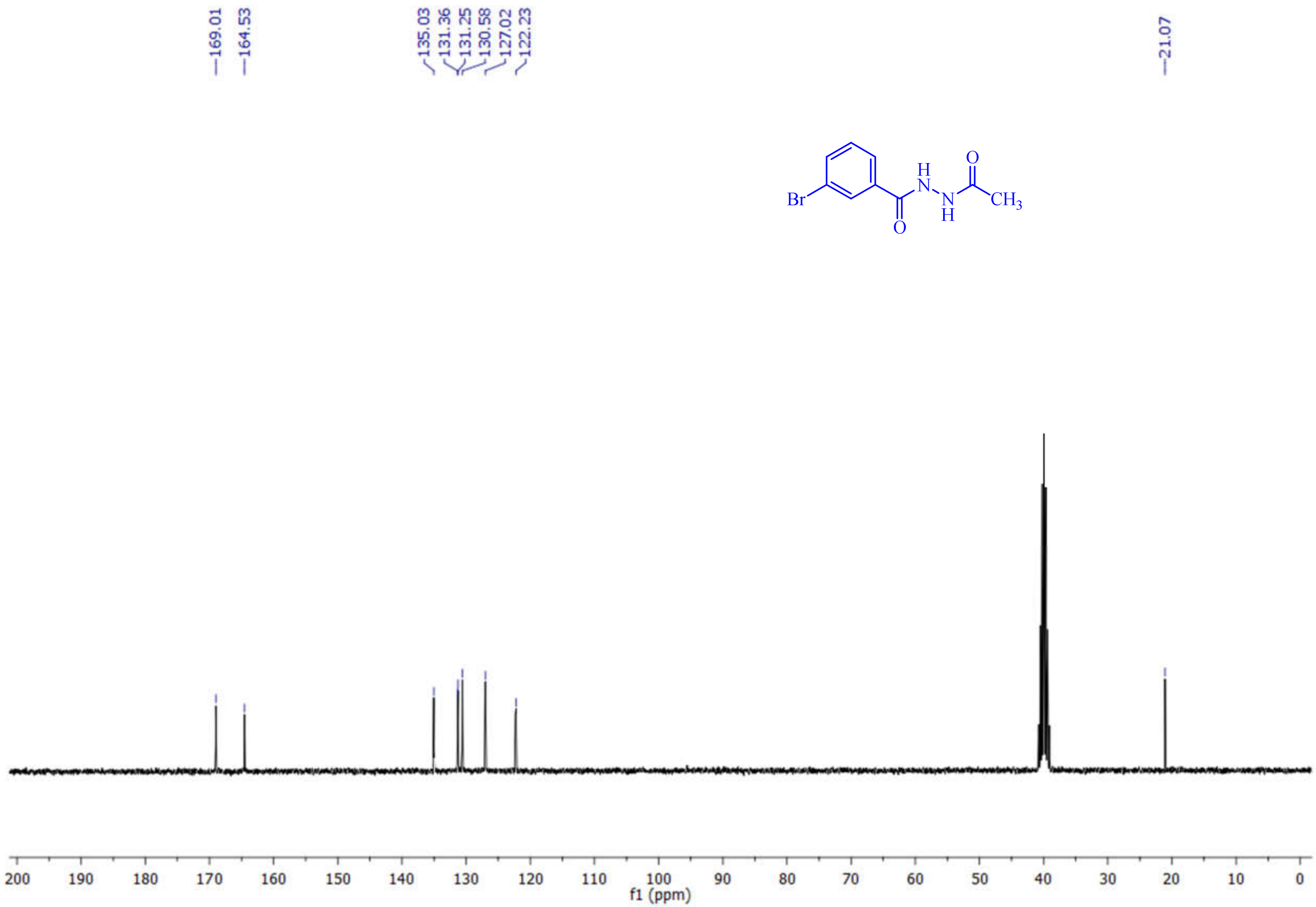

Fig. 41 ^{13}C NMR of *N'*-acetyl-3-bromobenzohydrazide (**2n**)

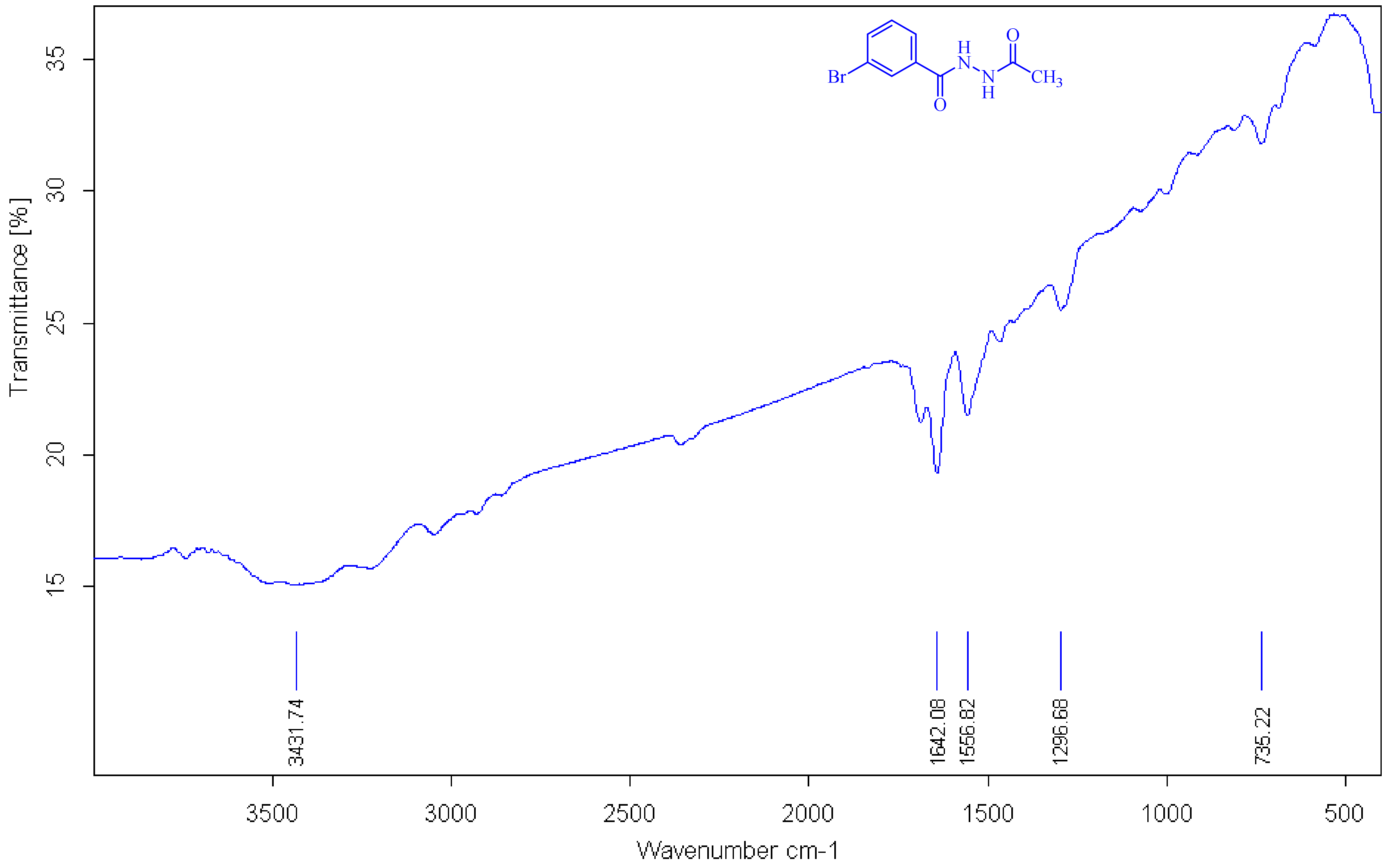

Fig. 42 FT-IR of *N*'-acetyl-3-bromobenzohydrazide (**2n**)

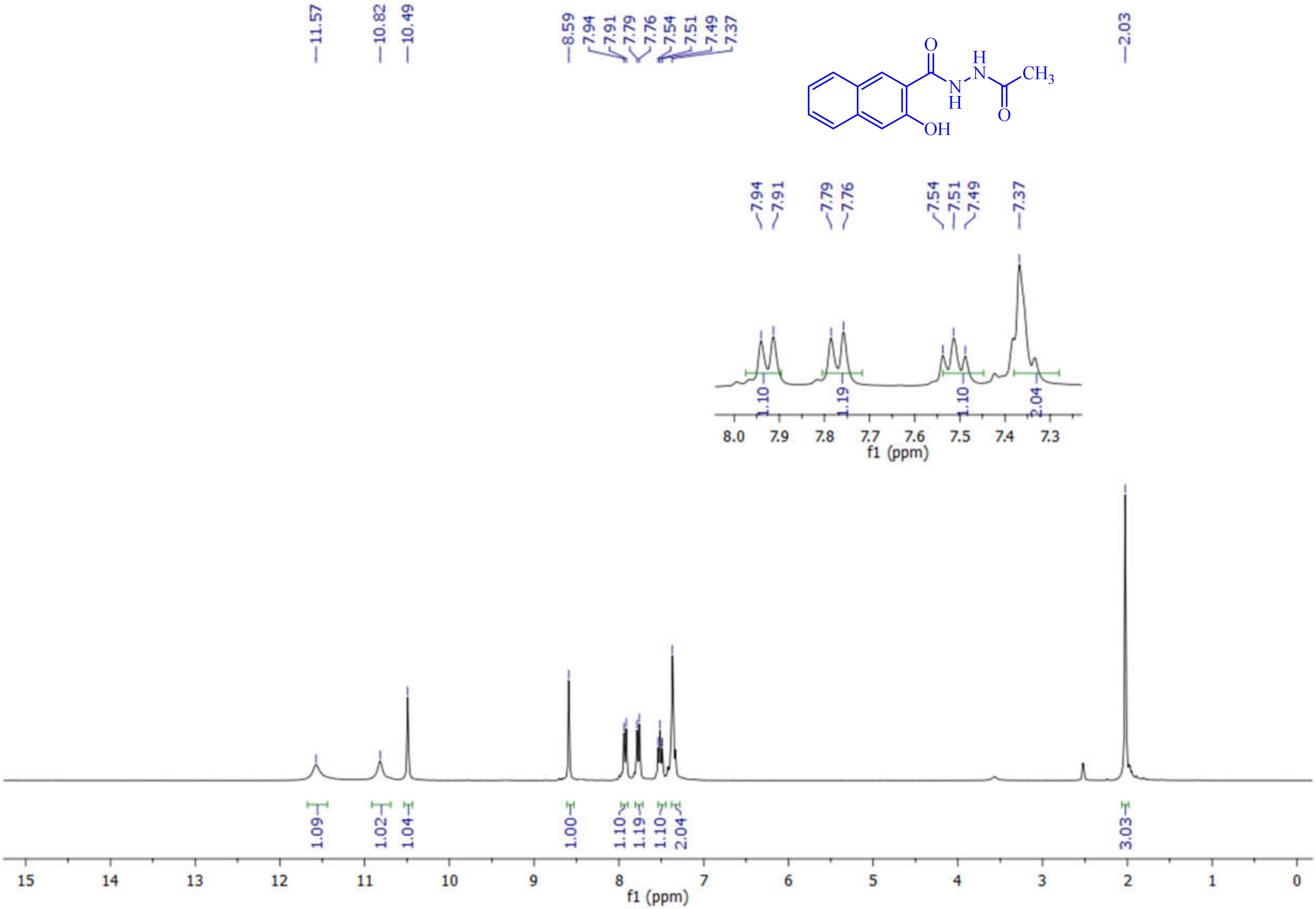

Fig. 43 ^{1}H NMR of *N'*-acetyl-3-hydroxy-2-naphthohydrazide (**2o**)

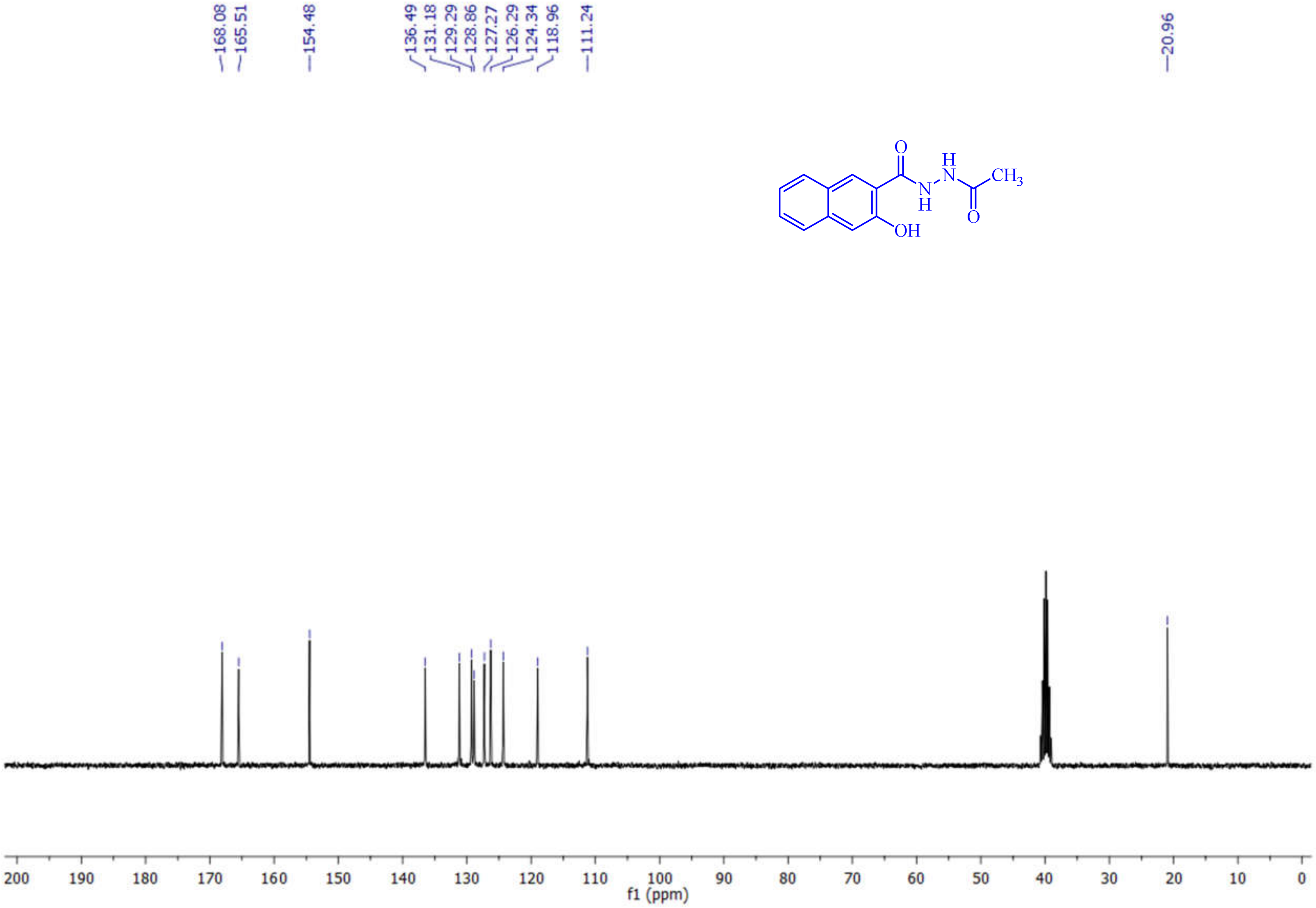

Fig. 44 ^{13}C NMR of *N*'-acetyl-3-hydroxy-2-naphthohydrazide (**2o**)

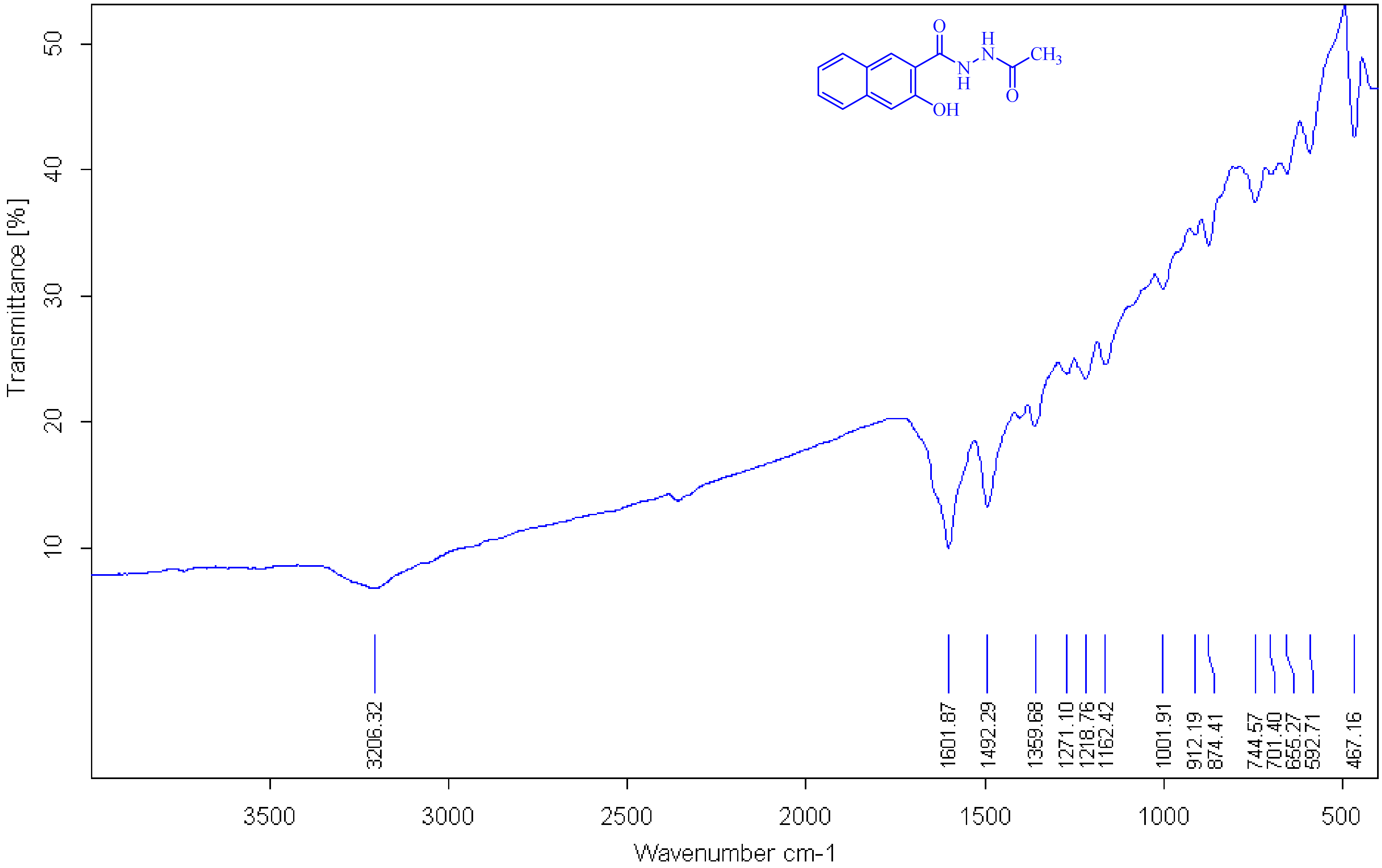

Fig. 45 FT-IR of *N'*-acetyl-3-hydroxy-2-naphthohydrazide (**2o**)

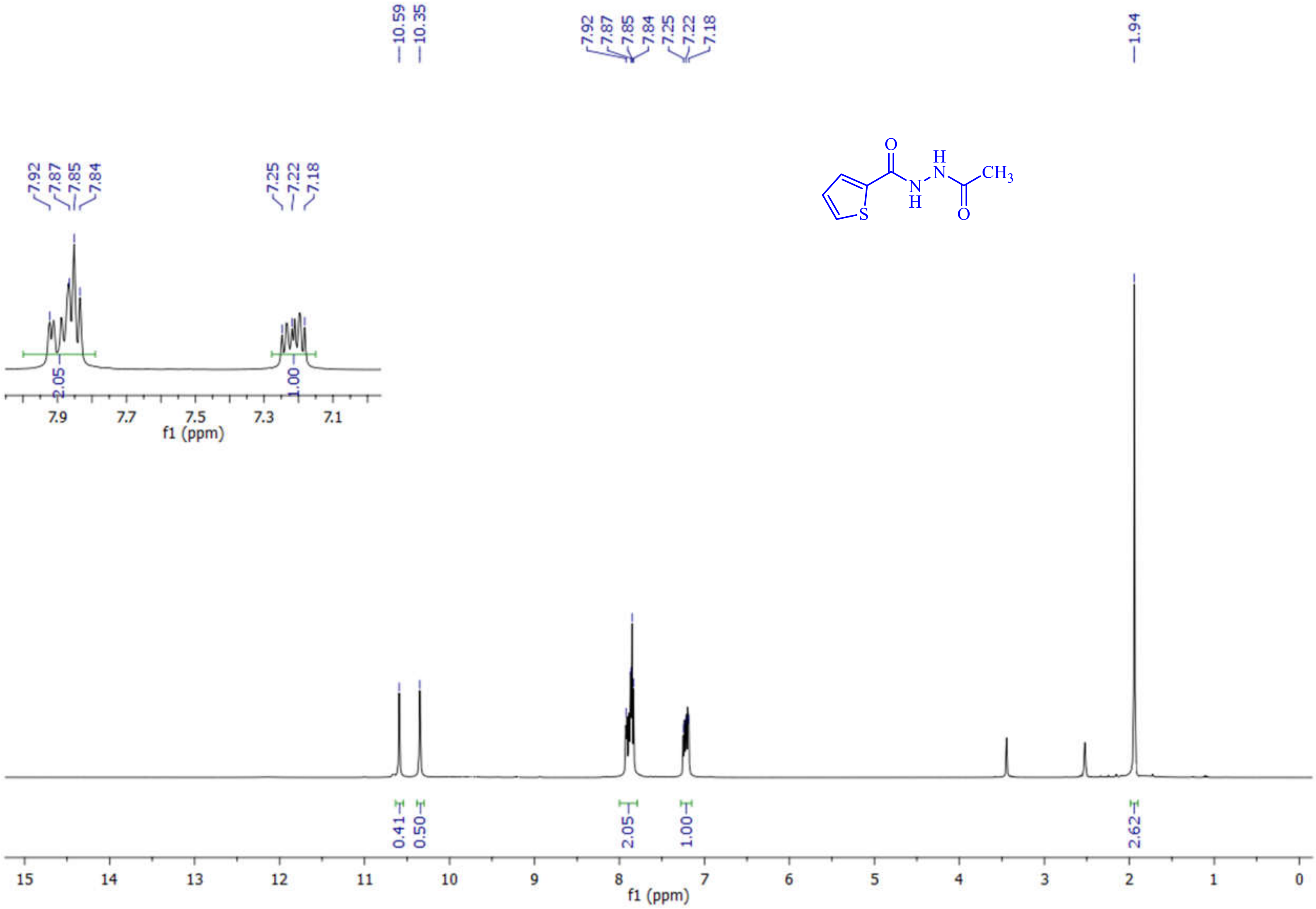

Fig. 46 ^{1}H NMR of *N*'-acetylthiophene-2-carbohydrazide (**2p**)

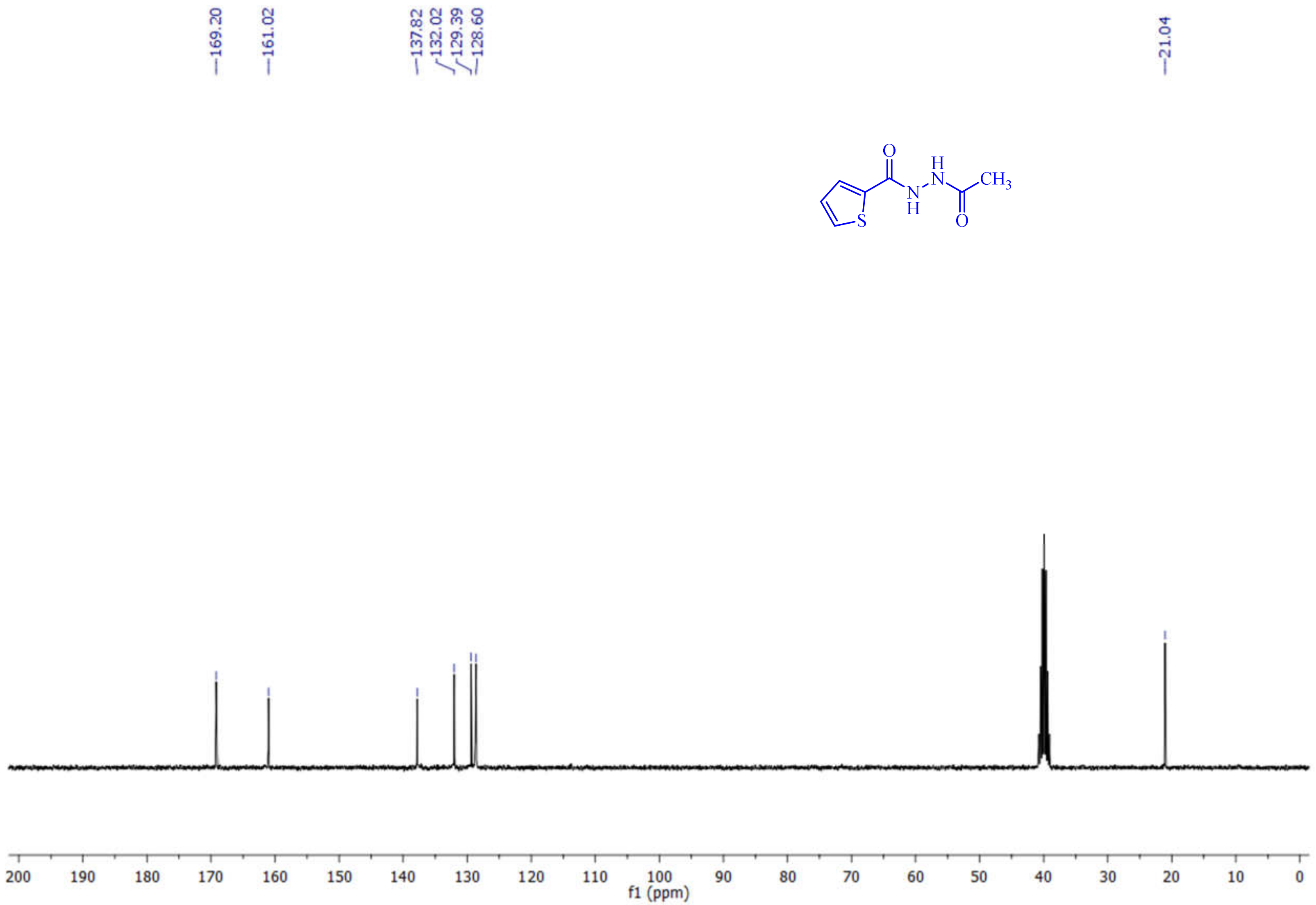

Fig. 47 ^{13}C NMR of *N'*-acetylthiophene-2-carbohydrazide (**2p**)

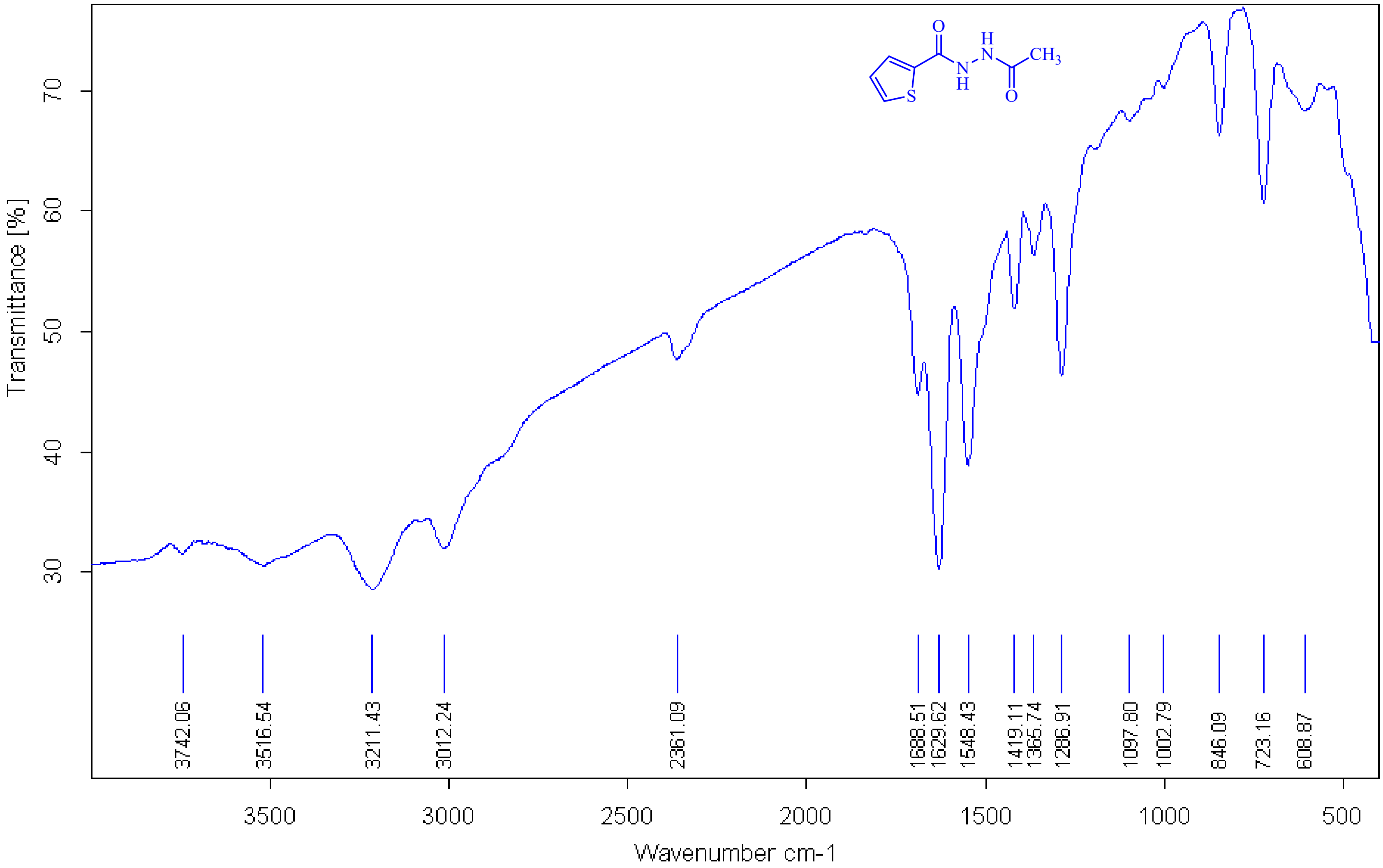

Fig. 48 FT-IR of *N'*-acetylthiophene-2-carbohydrazide (**2p**)

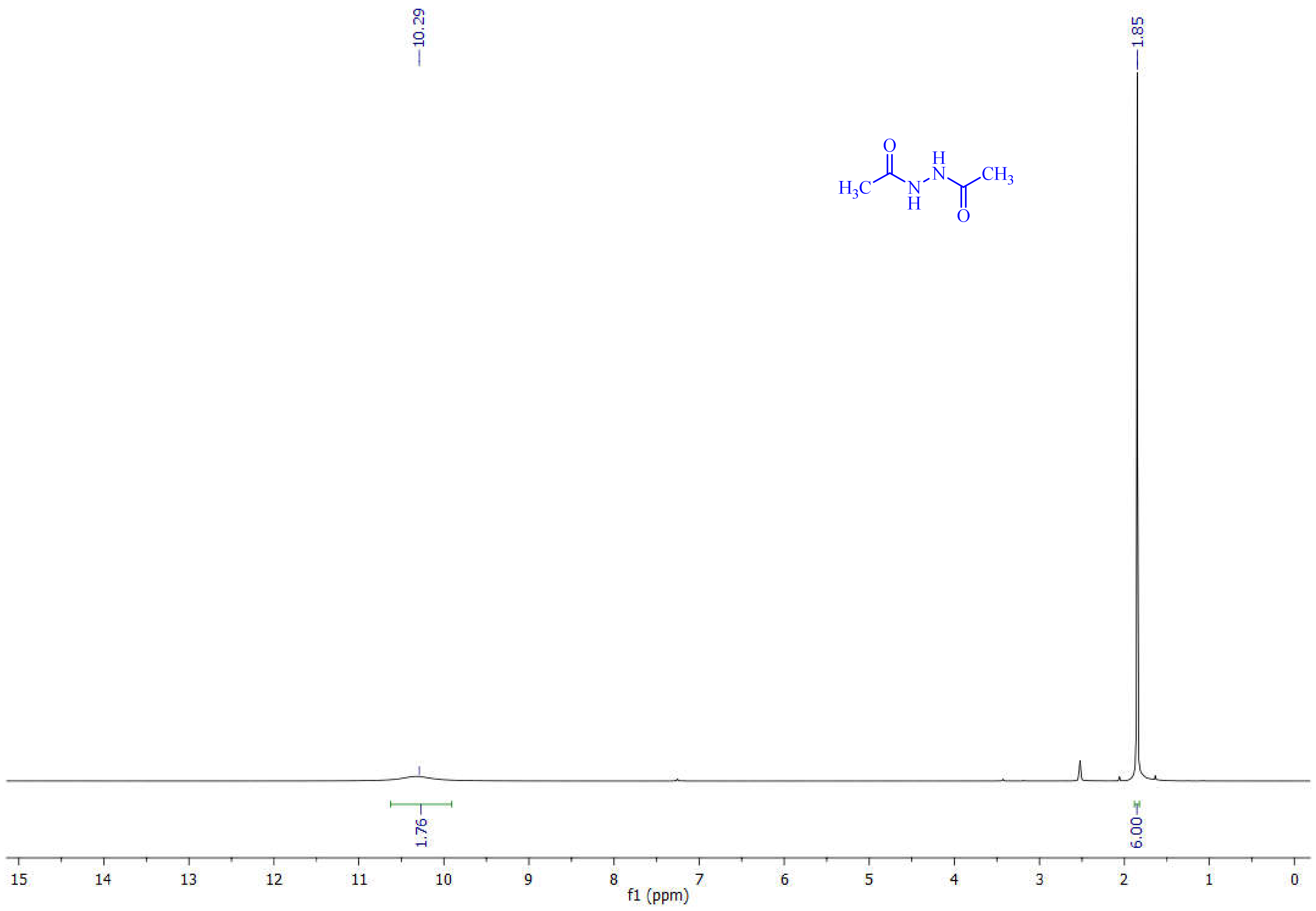

Fig. 49 ^{1}H NMR of *N'*-acetylacetohydrazide (**2q**)

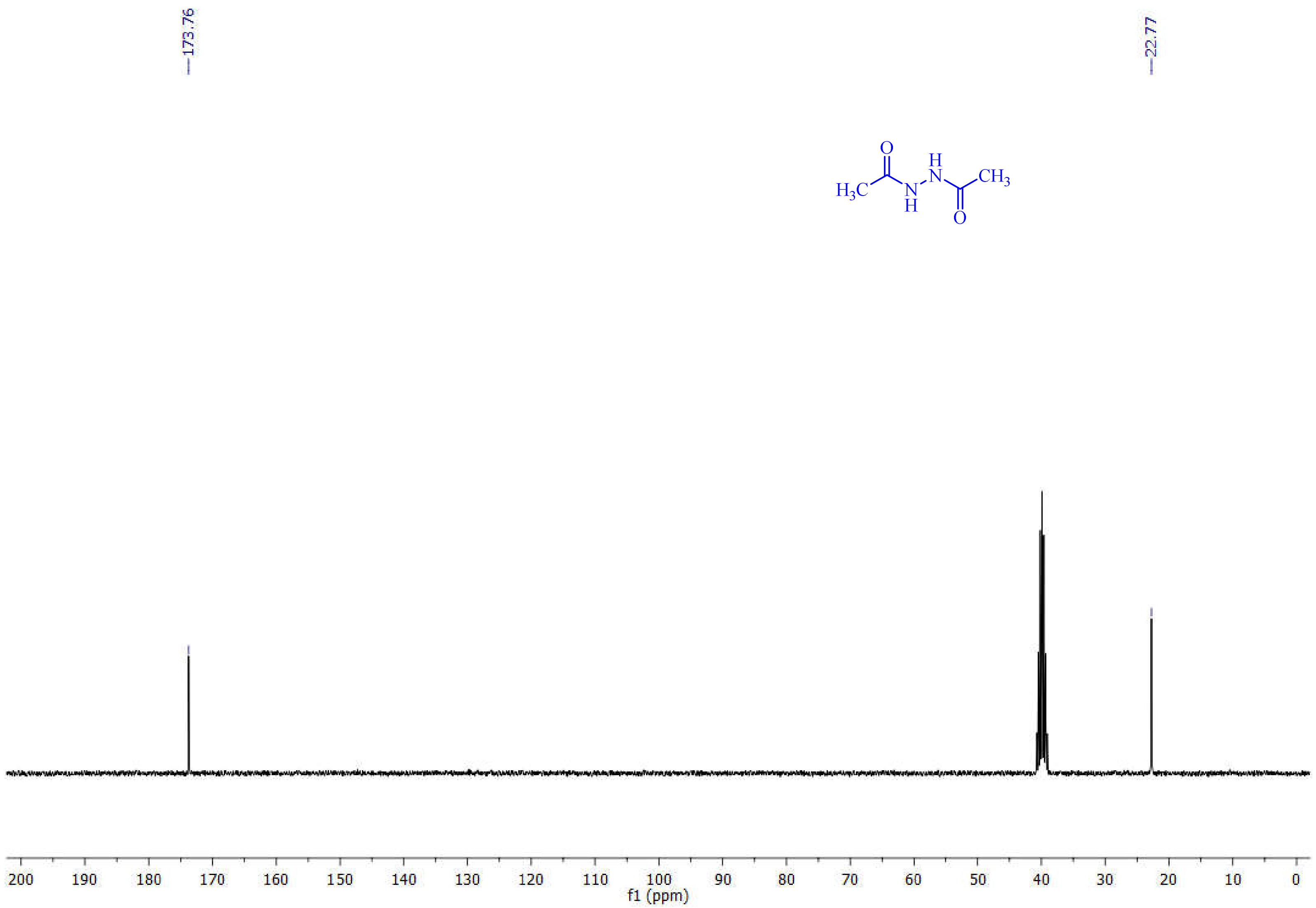

Fig. 50 ^{13}C NMR of *N*'-acetylacetohydrazide (**2q**)

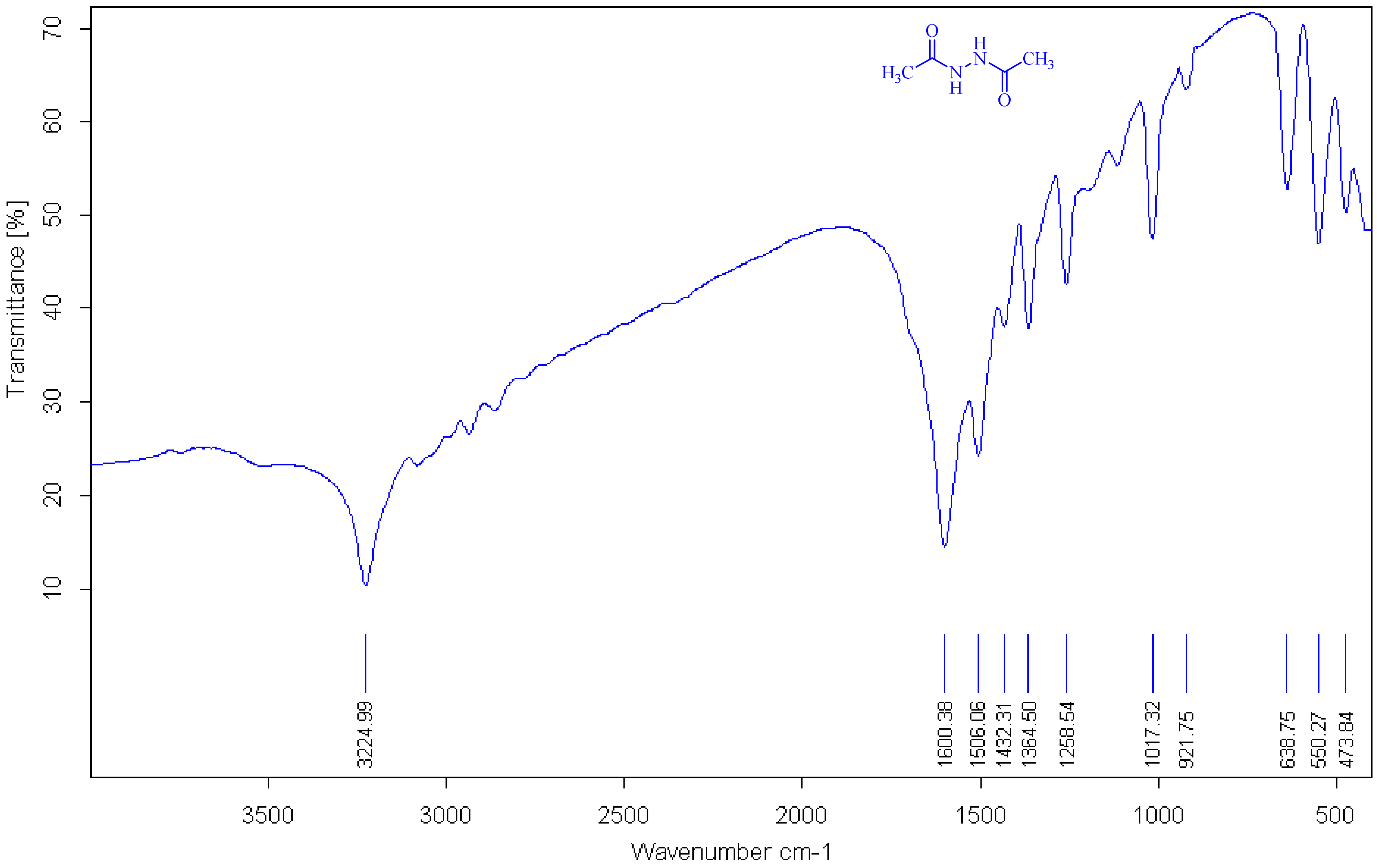

Fig. 51 FT-IR of *N'*-acetylacetohydrazide (**2q**)
